# Preparation and Application of an Inexpensive α-Formylglycine Building Block Compatible with Fmoc Solid-Phase Peptide Synthesis

**DOI:** 10.1021/acs.orglett.2c04059

**Published:** 2023-01-20

**Authors:** Nicholas D. J. Yates, Matthew E. Warnes, Reuben Breetveld, Christopher D. Spicer, Nathalie Signoret, Martin Fascione

**Affiliations:** † Department of Chemistry, University of York, York YO10 5DD, U.K.; ‡ Hull York Medical School, University of York, York YO10 5DD, U.K.

## Abstract

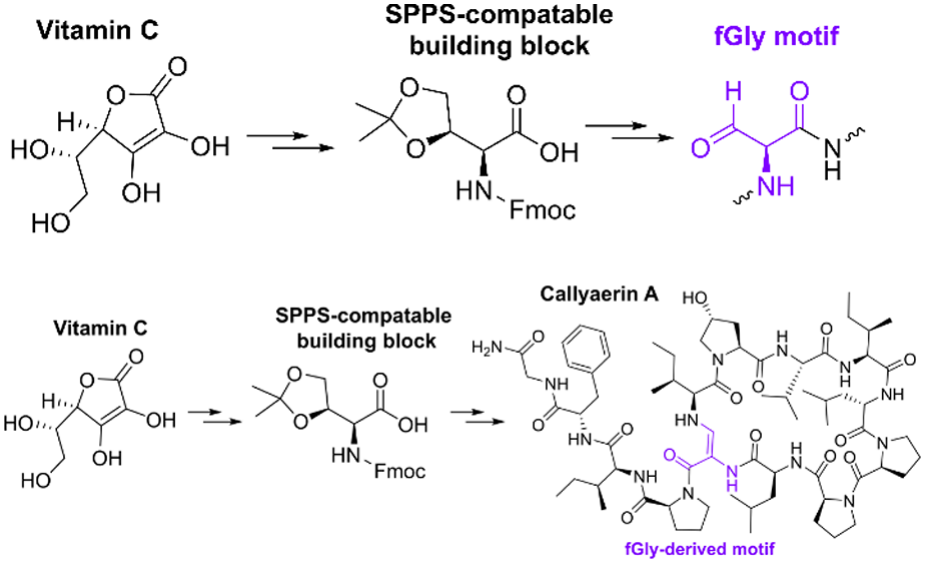

α-Formylglycine (fGly) is a rare residue located in the active site of sulfatases and serves as a precursor to pharmaceutically relevant motifs. The installation of fGly motifs into peptides is currently challenging due to degradation under the acidic and nucleophile-rich conditions accompanying resin cleavage during solid-phase peptide synthesis. We report the synthesis of acid- and nucleophile-tolerant α-formylglycine building blocks from vitamin C and use them to prepare callyaerin A, a macrocyclic peptide containing an fGly-derived motif.

In both eukaryotes and prokaryotes, enzymes capable of introducing α-formylglycine (fGly) residues (Scheme 1) into proteins prior to folding are essential for making post- or cotranslational modifications to type I sulfatases^1^ and some phosphonate monoester hydrolases/phosphodiesterases.^2^ A fGly residue is generated by the selective oxidation of a cysteine or serine residue embedded within a conserved consensus sequence (C/S)XPXRXXXLTG^1b,3^ by either formylglycine generating enzymes (FGEs)^4^ or anaerobic sulfatase maturating enzymes (anSMEs) (Scheme 1).^3b,5^ Oxidation of this residue is used to introduce an active-site aldehyde functionality into the sulfatase/phosphonate monoester hydrolase/phosphodiesterases, which when hydrated to a geminal diol serves as a nucleophile, allowing these enzymes to cleave sulfate/phosphonate esters.^1,6^ Incorporation of fGly consensus sequences into recombinant proteins can be used in conjugation with FGEs or anSMEs to produce fGly-labeled proteins, in which the fGly motif can serve as a unique bioconjugation handle.^7^

**Scheme 1:**
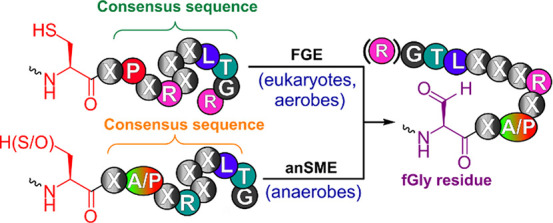
Oxidation of Cysteine or Serine Residues by Either FGE or anSME Enzymes to Yield Formylglycine Residues^1b,3−5^

FGly-functionalized peptides are therefore useful in studies involving sulfatases, phosphonate monoester hydrolases/phosphodiesterases, FGEs, and an SMEs, and would also prove useful in the development of bioconjugation techniques intended for selectively targeting fGly residues.^7a,7b,8^ Additionally, fGly can also be used as a handle through which pharmaceutically important rigid diaminoacrylamide motifs can be installed into peptides.^9^

Whereas chemical approaches for fGly installation into peptides have been reported,^9a,9b^ these methods currently suffer from limitations associated with the intolerance of fGly to the acidic conditions and nucleophiles (e.g., silanes and thiol-based scavengers^9a^ or cleaved 2,2,4,6,7-pentamethyldihydrobenzofuran-5-sulfonyl (Pbf) protecting groups) during the resin-cleavage step of solid-phase peptide synthesis (SPPS) (see Figure S51).^9b,9d^ Herein we describe the design and total synthesis of an SPPS-compatible fGly-precursor building block from vitamin C: a cheap, readily available chiral feedstock (Scheme 2A). We demonstrate that this building block is compatible with conventional and microwave-assisted SPPS and prepare the pharmaceutically relevant peptide callyaerin A; a potent antituberculosis macrocyclic peptide which contains a (*Z*)-2,3-diaminoacrylamide moiety derived from an fGly residue inaccessible to FGE/anSME-based enzymatic efforts.^9d,10^

**Scheme 2:**
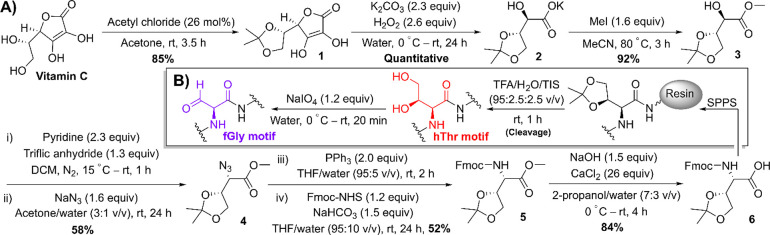
(A) Preparation of Protected fGly Precursor **6** from Vitamin C. (B) Preparation of fGly-Functionalized Peptides Using Protected fGly Precursor **6**

Oxidative cleavage of 1,2-diols^11^ or 1,2-amino alcohols^12^ using periodate is a mild and commonly used method for the installation of aldehydes into biomolecules. 1,2-Diols present in glycans and other biomolecules are highly susceptible to periodate oxidation,^11^ yet are tolerant to the conditions required for resin cleavage after SPPS. We thus envisioned installing 1,2-diol-functionalized 4-hydroxy-l-threonine (hThr) residues into peptides as formyl glycine precursors for the subsequent generation of fGly residues after resin cleavage via mild sodium periodate oxidation (Scheme 2B). We hypothesized this approach would overcome issues associated with the intolerance of fGly residues to the conditions encountered during SPPS resin cleavage.

Methyl 3,4-*O*-isopropylidene-l-threonate **3** was identified as a commercially available chiral feedstock and was itself synthesized in-house in excellent yield using inexpensive reagents in three steps from vitamin C (Scheme 2A).^13^ Subsequent substitution of the secondary alcohol in **3** via S_N_2 attack with sodium azide, followed by Staudinger reduction, Fmoc-protection, and methyl ester hydrolysis, allowed *N*-Fmoc-protected SPPS-compatible hThr l-amino acid **6** to be accessed in a further four steps from **3**. The additional chiral center of the protected 1,2-diol motif allowed **6** to be verified as a pure l-amino acid by NMR.

fGly building block **6** was used to prepare a test peptide NH_2_-Gly-Leu-Tyr-Arg-hThr-Ala-Gly-COOH **7** using Fmoc SPPS in a total yield of 95%, with HPLC analysis of **7** showing it to be of excellent purity (Figure 1). No acid-catalyzed breakdown products were observed during the cleavage of **7** from the peptide resin, and no side-products derived from the reaction of fGly with the cleaved Pbf group of the Arg residue were observed. Conversion of the hThr residue of **7** into fGly was then readily achieved via treatment of a 2 mM aqueous solution of **7** with 1.2 equiv of sodium metaperiodate (NaIO_4_), yielding peptide **8** (Figure 1). Peptides **7** and **8** were found to have the same retention time in LC–MS (most likely due to the similar polarities of hydrated fGly and hThr motifs), but mass spectrometry peaks attributable to **7** were fully lost within 20 min of treatment with NaIO_4_, leaving only signals attributable to **8** (Figure 1). Additionally, while oxidation of serine/threonine residues to fGly using RuO_4_ is known to lead to side-products via oxidative scission/retroaminal fragmentation,^9a,14^ no such byproducts were detected after the treatment of **7** with NaIO_4_ (see Figure S52).

**Figure 1:**
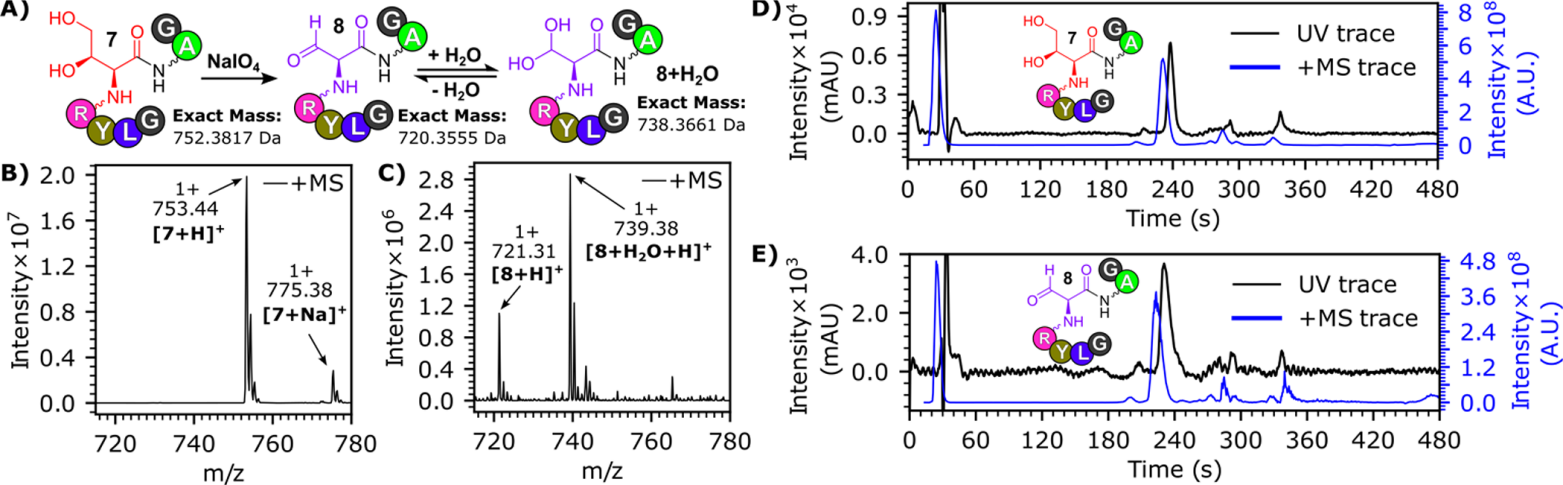
(A) Conversion of peptide **7** into peptide **8** using NaIO_4_. (B) MS analysis of **7**. (C) MS analysis of **8**. (D) LC–MS chromatogram of **7**. (E) LC_-MS chromatogram of **8**. LC_-MS chromatograms show the BPC+All MS trace and the UV chromatogram shows the absorbance for wavelengths 210–400 nm. LC_MS was conducted using LC gradient A (see Figure S1).

Having verified the compatibility of **6** with SPPS, and with conditions established for fGly installation via periodate treatment, we applied the methodology to the synthesis of callyaerin A **10**,^9c,9d^ which contains a rare (*Z*)-2,3-diaminoacrylamide motif derived from an fGly residue (Figure 2).^9a,9d^ The only previous synthesis of **10** used an alternative Fmoc-protected building block but reported problems associated with the epimerization of this building block in SPPS, in addition to acid-catalyzed fragmentation of the fGly-functionalized linear callyaerin A peptide upon resin cleavage.^9d^ By contrast, we hypothesized that a comparable linear peptide constructed using **6** would be far less prone to acid-catalyzed fragmentation during resin cleavage.

**Figure 2:**
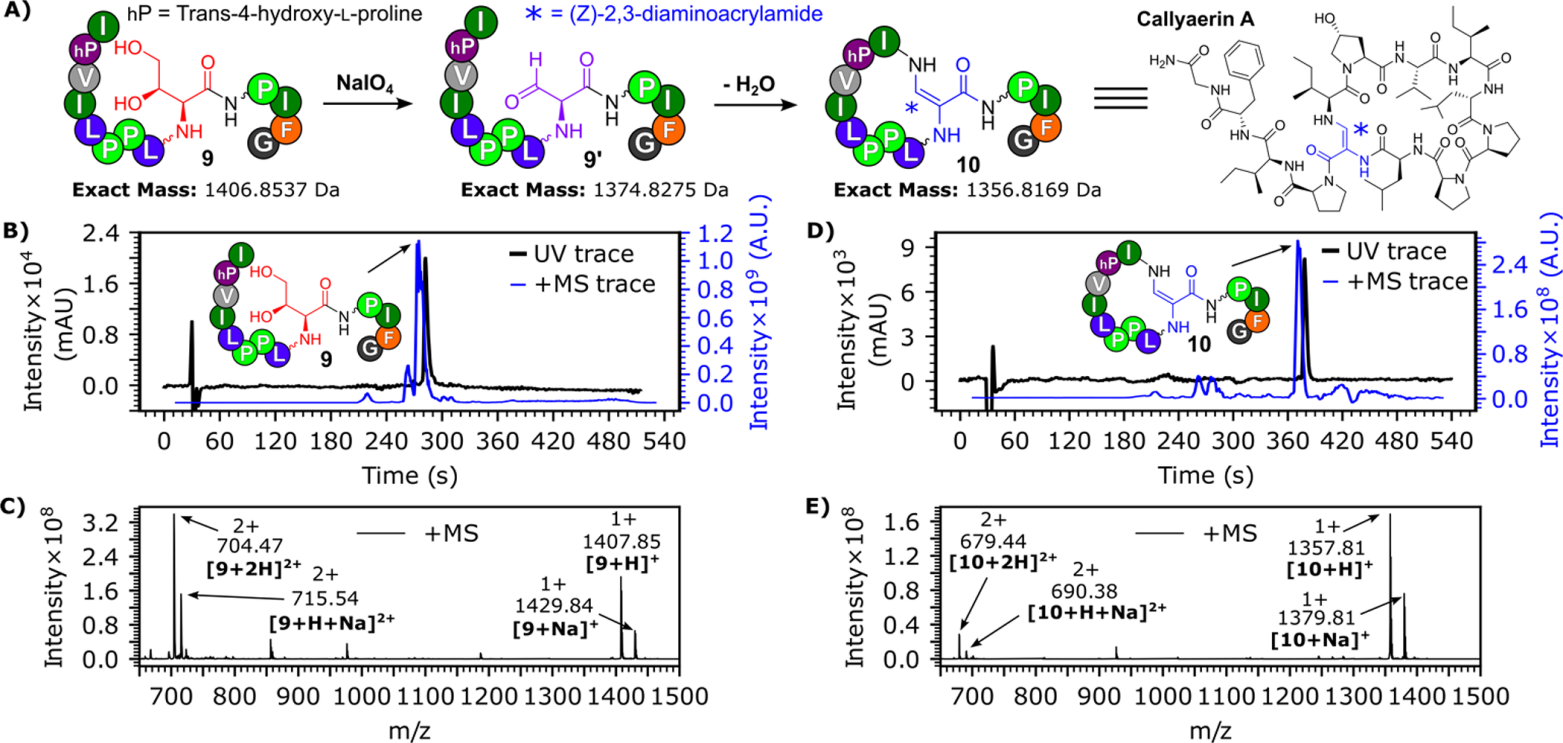
(A) Conversion of linear callyaerin A precursor **9** into callyaerin A **10** (via **9′**) using NaIO_4_. (B) LC–MS chromatogram of **9**. (C) Mass spectrum of **9**. (D) LC–MS chromatogram of **10**. (E) Mass spectrum of **10**. Note that the peak at *m*/*z* 926.57 corresponds to a b_2_ fragment ion of **10** (Figure S50). LC–MS chromatograms show the BPC+All MS trace and the UV chromatogram shows the absorbance for wavelengths 210–400 nm. LC–MS was conducted using LC gradient B (see Figure S2). For MS evidence of **9′** see Figure S53.

Microwave-assisted fully automated Fmoc SPPS was used to prepare linear callyaerin A precursor **9** (Figure 2), verifying that **6** is suitable for use in microwave-assisted peptide synthesis. A 2 mM solution of **9** was then treated with 1.2 equiv of NaIO_4_, and the hThr residue of **9** was cleanly converted to fGly in a one-pot reaction within 20 min, yielding a mixture of linear callyaerin A **9′** and callyaerin A **10** (see Figure S53). Complete cyclization of **9′** into **10** was facilitated via the extraction of the peptide mixture into MeCN and the addition of 0.1% formic acid and MgSO_4_. Due to the profound change in the shape accompanying cyclization, **9** and **10** have distinct retention times as well as distant mass spectrometry peaks (Figure 2).

In summary, Fmoc-protected hThr building block **6** can be readily synthesized from vitamin C using inexpensive reagents and used in SPPS to install hThr residues into peptides. Utilization of hThr as a precursor to fGly can circumvent incompatibility issues associated with the intolerance of fGly toward the conditions/species encountered during SPPS, and the hThr residue can then be cleanly converted to fGly off-resin using NaIO_4_. We demonstrated that our approach can not only be used to prepare fGly-functionalized peptides but also used to prepare cyclic peptides bearing pharmaceutically-important rigidifying motifs. Although under harsh conditions amino acids such as Met, Trp, Tyr, and Ser/Thr can be susceptible periodate oxidation,^15^ by performing reactions at neutral pH and low temperature with controlled reaction stoichiometry, akin to the conditions used here, these residues have been shown to tolerate NaIO_4_.^11a,16^ Furthermore, addition of excess Met to the reaction has been shown to further reduce overoxidation of peptides with no loss of biological activity, notably in the case of the polypeptide chemokine RANTES which contains Met and also multiple disulfide forming Cys residues,^17^ which are highly susceptible to periodate oxidation.^18^ We therefore propose our building block may have broad applications in the fields of bioconjugation and pharmacology, particularly as bioconjugations using formylglycine are currently underexplored, the biological mechanisms of formylglycine installation has not yet been unequivocally elucidated^19^ and formylglycine-derived motifs are present in many pharmaceutically-relevant compounds, including the tuberactinomycin^9a,20^ and callyaerin^9a,9c,9d,10,21^ families of antibiotics.

## Data Availability

The data underlying this study are available in the published article and its Supporting Information.

## Supporting Information Available

Experimental procedures and spectroscopic and analytical data for all new compounds, including copies of the NMR spectra (PDF)
