# Supplementary material for: Preparation and Application of an Inexpensive α-Formylglycine Building Block Compatible with Fmoc Solid-Phase Peptide Synthesis
Source: Org Lett. 2023 Jan 20;25(12):2001–5. doi: 10.1021/acs.orglett.2c04059 (PMC10071478; doi:10.1021/acs.orglett.2c04059)
Supplement: Supplementary file 1 — ol2c04059_si_001.pdf [file ol2c04059_si_001.pdf]

Preparation and Application of an Inexpensive α-Formylglycine Building Block Compatible with Fmoc Solid-Phase Peptide Synthesis

Nicholas D. J. Yates, [a]* Matthew E. Warnes,[a] Reuben Breetveld,[a] Christopher D. Spicer,[a] Nathalie Signoret,[b] Martin Fascione [a]*

a. Department of Chemistry, University of York, York, YO10 5DD, UK

b. Hull York Medical School, University of York, York, YO10 5DD, UK

* Corresponding authors. *Email:* nicholas.yates@york.ac.uk, *Email:* martin.fascione@york.ac.uk

Supporting information

Contents

General considerations **2**

Synthesis and characterisation of small molecule probes **4**

5,6-O-isopropylidene-L-ascorbic acid **1** 4

Potassium-3,4-O-isopropylidene-L-threonate **2** 8

Methyl 3,4-O-isopropylidene-L-threonate **3** 13

(Methyl (S)-2-azido-2-((R)-2,2-dimethyl-1,3-dioxolan-4-yl)acetate **4** (via **3'**) 19

Methyl (2S)-[(4R)-2,2-dimethyl-1,3-dioxolan-4-yl]({[(9H-fluoren-9-yl)methoxy]carbonyl}amino) acetate **5** .. 27

(2S)-[(4R)-2,2-dimethyl-1,3-dioxolan-4-yl]({[(9H-fluoren-9-yl)methoxy]carbonyl}amino)acetic acid **6** 36

Test peptide **7** 44

Test peptide **8** 45

Linear Callyaerin A precursor **9** 47

Callyaerin A **10** 49

Additional Figures **58**

References **62**

General considerations

Anhydrous solvents were dried over a PureSolv MD 7 Solvent Purification System. Anhydrous solvents were used in reactions unless otherwise stated, or where aqueous/organic cosolvent mixtures were employed. GPR-grade solvents were used for flash chromatography purposes. Solution-phase synthetic reactions were carried out using oven-dried glassware. All concentrations were performed *in vacuo* unless otherwise stated. Thin layer chromatography was carried out on Merck silica gel 60 F254 precoated aluminium foil sheets and these were visualized using UV light (254 nm) and/or PPh_3 (10% in DCM) and/or ninhydrin (1.5% ninhydrin, 3% AcOH in n-butanol), and/or H_2SO_4 (5% H_2SO_4 in MeOH). Unless otherwise indicated, flash column chromatography was performed on Supelco® silica gel (particle size 35–75 µm, pore diameter 60 Å, 220-440 mesh) and the solvent system used is recorded in parentheses.

Reagents (including peptide coupling reagents) were purchased from Sigma-Aldrich and used as supplied, unless otherwise indicated. Fmoc-protected amino acids were purchased from Fluorochem.

Proton and carbon nuclear magnetic resonance (^1H and ^{13}C NMR respectively) spectra were recorded on either a Jeol ECX-400 (400 MHz) or a Bruker Neo 700MHz spectrometer. Assignments of NMR spectra were conducted on MestReNova, using additional NMR experiments including ^1H-^1H-COSY, DEPT and ^1H-^{13}C-HMQC spectra when necessary. The numbering system for NMR assignments of compounds does not follow IUPAC rules. All chemical shifts are quoted on the δ scale in ppm using residual solvent as the internal standard (^1H NMR: $CDCl_3$ = 7.26; methanol-d_4 = 3.31; D_2O = 4.69; DMSO-d_6 = 2.50 and ^{13}C NMR: $CDCl_3$ = 77.16, methanol-d_4 = 49.00, DMSO-d_6 = 39.52). Coupling constants (J) are reported in Hz with the following splitting abbreviations: s = singlet, d = doublet, t = triplet, q = quartet, m = multiplet, app = apparent, br = broad.

Melting points (m.p.) were recorded on a Gallenkamp melting point apparatus. Fourier transform infrared (FT-IR) spectra were recorded on a PerkinElmer UATR 2 spectrometer using the attenuated total reflectance (ATR) technique. Absorption maxima (υmax) are reported in wavenumbers (cm^{-1}).

Optical rotations were measured using a Bellingham and Stanley ADP 450 Automatic Digital Peltier Controlled Polarimeter equipped with a 589 nm LED and a path length of 1 dm. $[\alpha]_D^{20}$ values reported in units of 10^{-1} deg cm^2 g^{-1}.

Small-molecule high resolution mass spectrometry (HRMS) data were obtained at RT on a Bruker Daltonics microTOF mass spectrometer coupled to an Agilent 1200 series LC system at The University York Centre of Excellence in Mass Spectrometry (CoEMS). Nominal and exact m/z values are reported in Daltons.

High Performance Liquid Chromatography-Electrospray Ionization Mass Spectrometry (LC-MS) of peptides was performed using a Dionex UltiMate® 3000 Ci Rapid Separation LC system equipped with an UltiMate® 3000 photodiode array detector probing at 210–400 nm, coupled to a HCT ultra ETD II (Bruker Daltonics) ion trap spectrometer, using an Accucore C18 column (150 × 2.1 mm, 2.6 µm particle size. The LC-MS apparatus was controlled using Chromeleon® 6.80 SR12 software (ThermoScientific), esquireControl version 6.2, Build 62.24 software (Bruker Daltonics), and Bruker compass HyStar 3.2-SR2, HyStar version 3.2, Build 44 software (Bruker Daltonics) at CoEMS. Water (solvent A) and acetonitrile (solvent B), both containing 0.1% formic acid, were used as the mobile

phase at a flow rate of 0.3 mL min^{-1}. LC traces were measured via UV absorption between 210-400 nm. Two LC gradients were used. Gradient A and Gradient B. These gradients were programmed as shown below:

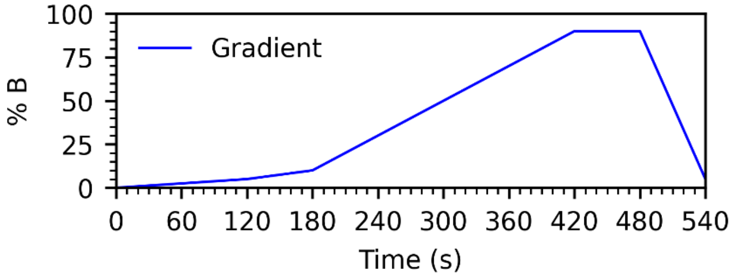

Figure S 1. LC gradient A.

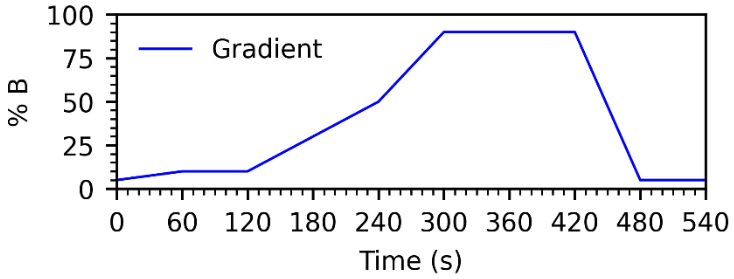

Figure S 2. LC gradient B.

Synthesis and characterisation of small molecule probes

5,6-O-isopropylidene-L-ascorbic acid 1

1 was synthesised using an adapted literature procedure.[1] To a solution of L-ascorbic acid (20 g, 114 mmol) in acetone (80 mL) was added a catalytic amount of acetyl chloride (2.1 mL, 30 mmol, 0.26 equiv) at rt. After stirring for 3.5 h at rt, the mixture was placed in the refrigerator overnight. The solid was then filtered off and washed with cold acetone to yield **1** as a white solid (26.5 g, 85%).

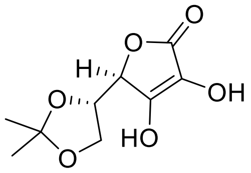

1

^{1}H-NMR (400 MHz, DMSO-d$_6$): δ_H 11.30 (s, 1H), 8.48 (s, 1H), 4.71 (d, *J* = 2.96 Hz, 1H), 4.26 (ddd, *J* = 7.05, 6.30, 2.96 Hz, 1H), 4.09 (dd, *J* = 8.42, 7.05 Hz, 1H), 3.88 (dd, *J* = 8.42, 6.30 Hz, 1H), 1.25 (s, 6H).

^{13}C-NMR (101 MHz, DMSO-d$_6$): δ_C 170.3, 152.4, 118.2, 109.0, 74.2, 73.5, 64.9, 25.8, 25.5.

FT-IR (ATR) (υmax/cm^{-1}): 3231 (O-H stretch), 2993 (C-H stretch), 1753 (C=O stretch), 1660 (C=C stretch), 1332, 1137 (C-O-C stretch), 1062.

[*α*]$_D$^{20}: +55 (*c* 1.0, H_2O) (lit.**[*α*]**$_D$^{25}: +7.0 (*c* 0.56, acetone))[2]

mp: 202 °C (lit.^{mp}: 195 – 200 °C)[1]

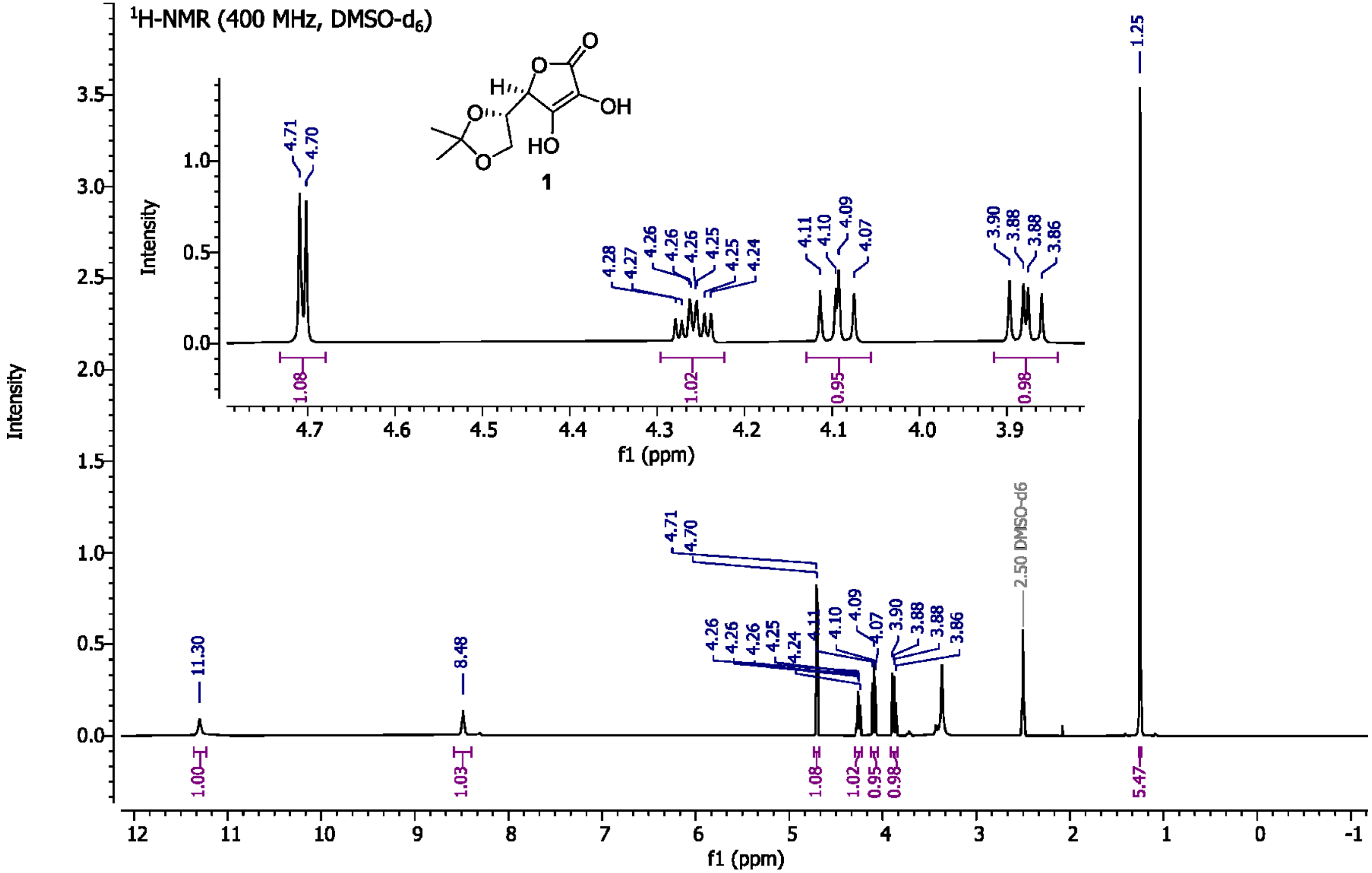

Figure S 3. ¹H-NMR spectrum of **1**.

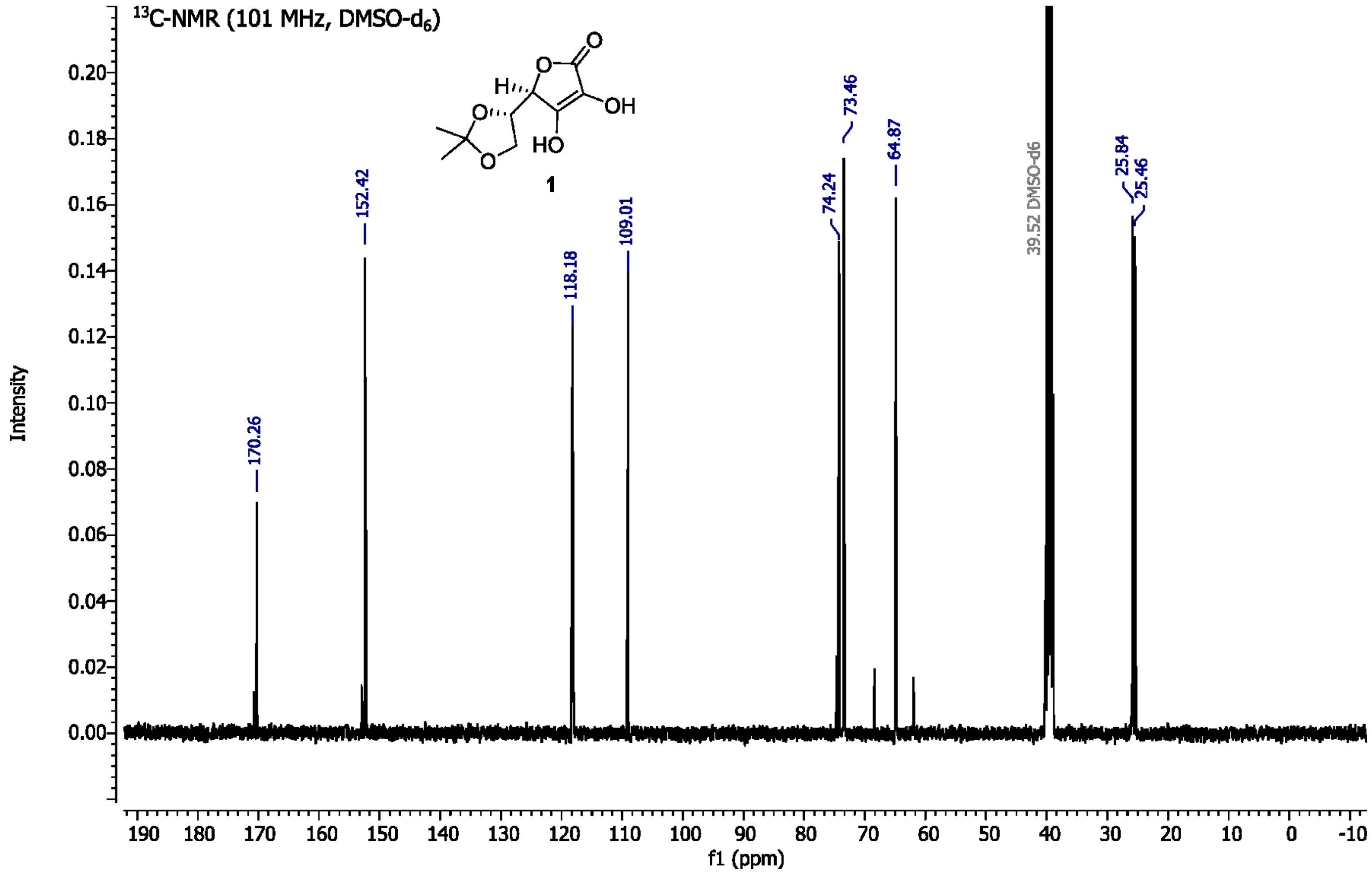

Figure S 4. ^{13}C-NMR spectrum of **1**.

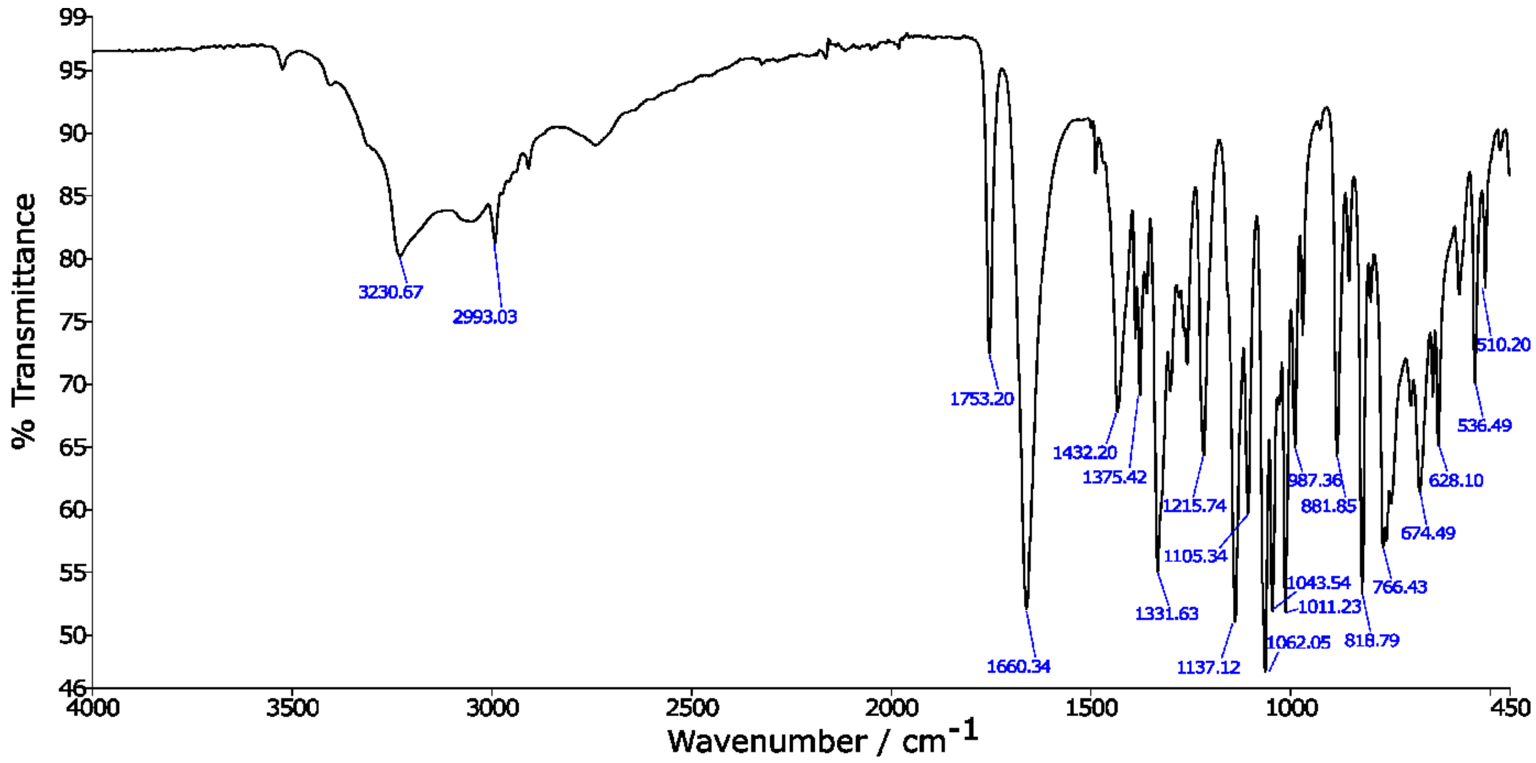

Figure S 5. FT-IR (ATR) spectrum of **1**.

Potassium-3,4-O-isopropylidene-L-threonate 2

To a stirred solution of potassium carbonate (59.2 g, 428 mmol) in water (227 mL) was added **1** (39.7 g, 184 mmol). The resultant solution was cooled to 0 °C and 30% aqueous H_2O_2 was added dropwise (47.9 mL, 469 mmol), taking care to make sure that the temperature of the reaction solution was kept below 10 °C. The resulting solution was then stirred at rt for 24 h, after which time it was concentrated *in vacuo* to yield a white solid. To the white solid was added EtOH (500 mL), and the resulting suspension refluxed with stirring for 30 minutes. Solids were removed by filtration while the EtOH remained hot using a sintered funnel and the eluate collected. The removed solids were again refluxed in EtOH (500 mL) for 30 minutes, removed via filtration and the eluate collected a further four times. The eluates were then combined and concentrated *in vacuo* to yield potassium-3,4-O-isopropylidene-L-threonate **2** as a white solid in quantitative yield (39.4 g).

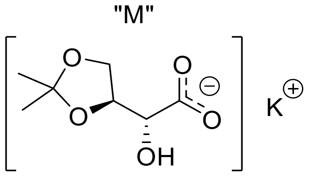

2

^{1}H-NMR (400 MHz, D_2O): δ_H 4.42 (ddd, J = 6.86, 6.56, 4.32 Hz, 1H), 4.14 (dd, J = 8.59, 6.86 Hz, 1H), 3.98-3.92 (m, 2H), 1.43 (s, 3H), 1.36 (s, 3H).

^{13}C-NMR (101 MHz, D_2O): δ_C 177.7, 109.9, 77.1, 72.2, 65.6, 25.3, 24.2.

HRMS (ESI) m/z: $[M+K+H]^+$ Calcd for $C_7H_{12}O_5K$ 215.0316; Found 215.0261. $[M+2Na]^+$ Calcd for $C_7H_{11}O_5Na_2$ 221.0396; Found 221.0404. $[M+Na+K]^+$ Calcd for $C_7H_{11}O_5NaK$ 237.0136; Found 237.0132.

FT-IR (ATR) (υmax/cm^{-1}): 3447 (O-H stretch, alcohol), 3224 (O-H stretch), 2984 (C-H stretch), 1581 (C-O asymmetric stretch, carboxylate), 1372 (C-O symmetric stretch, carboxylate), 1123 (C-O stretch, alcohol), 1066.

$[\alpha]_D^{20}$: +46 (c 1.0, H_2O)

mp: 138 °C

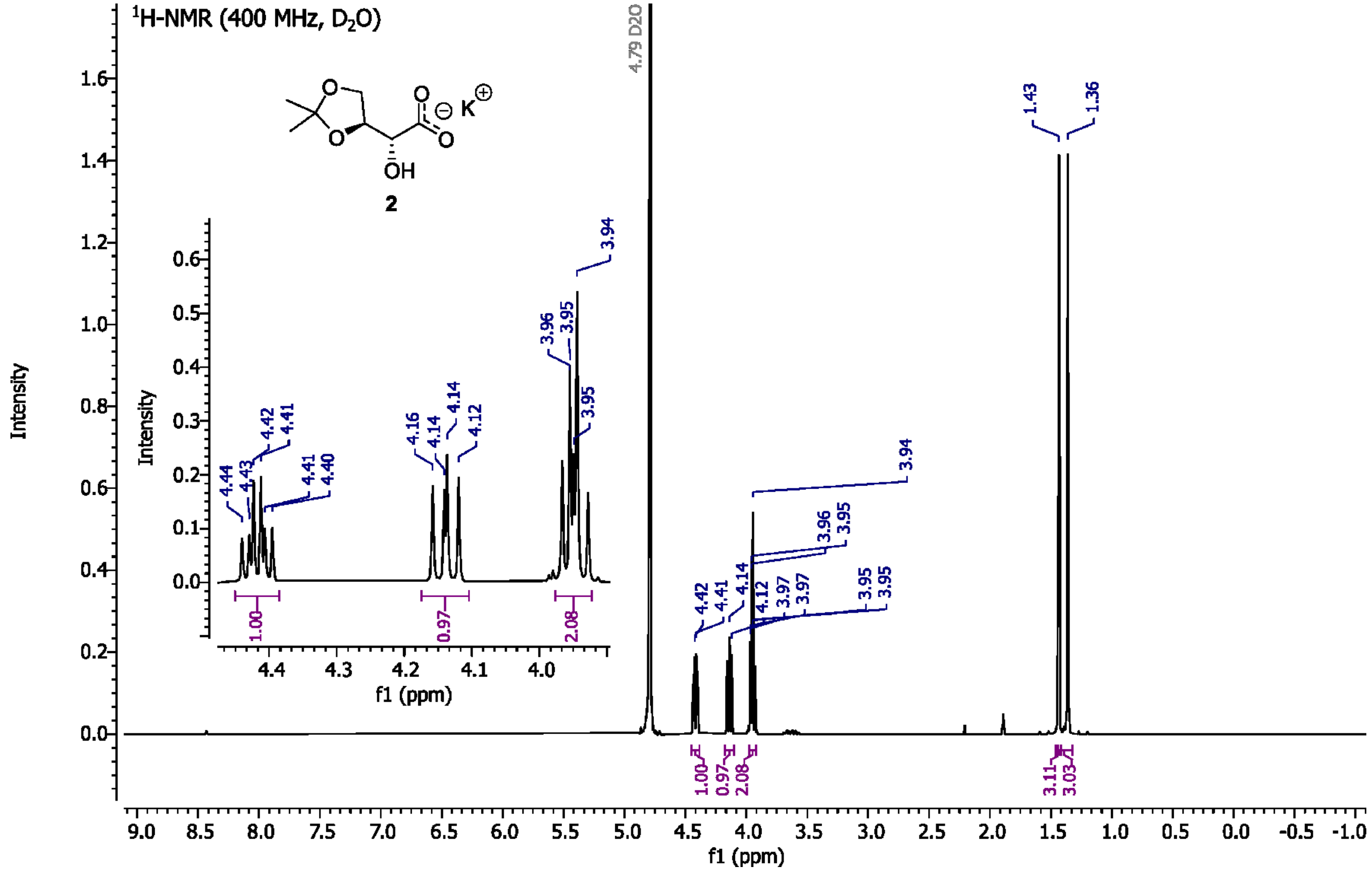

Figure S 6. ^{1}H-NMR spectrum of **2**.

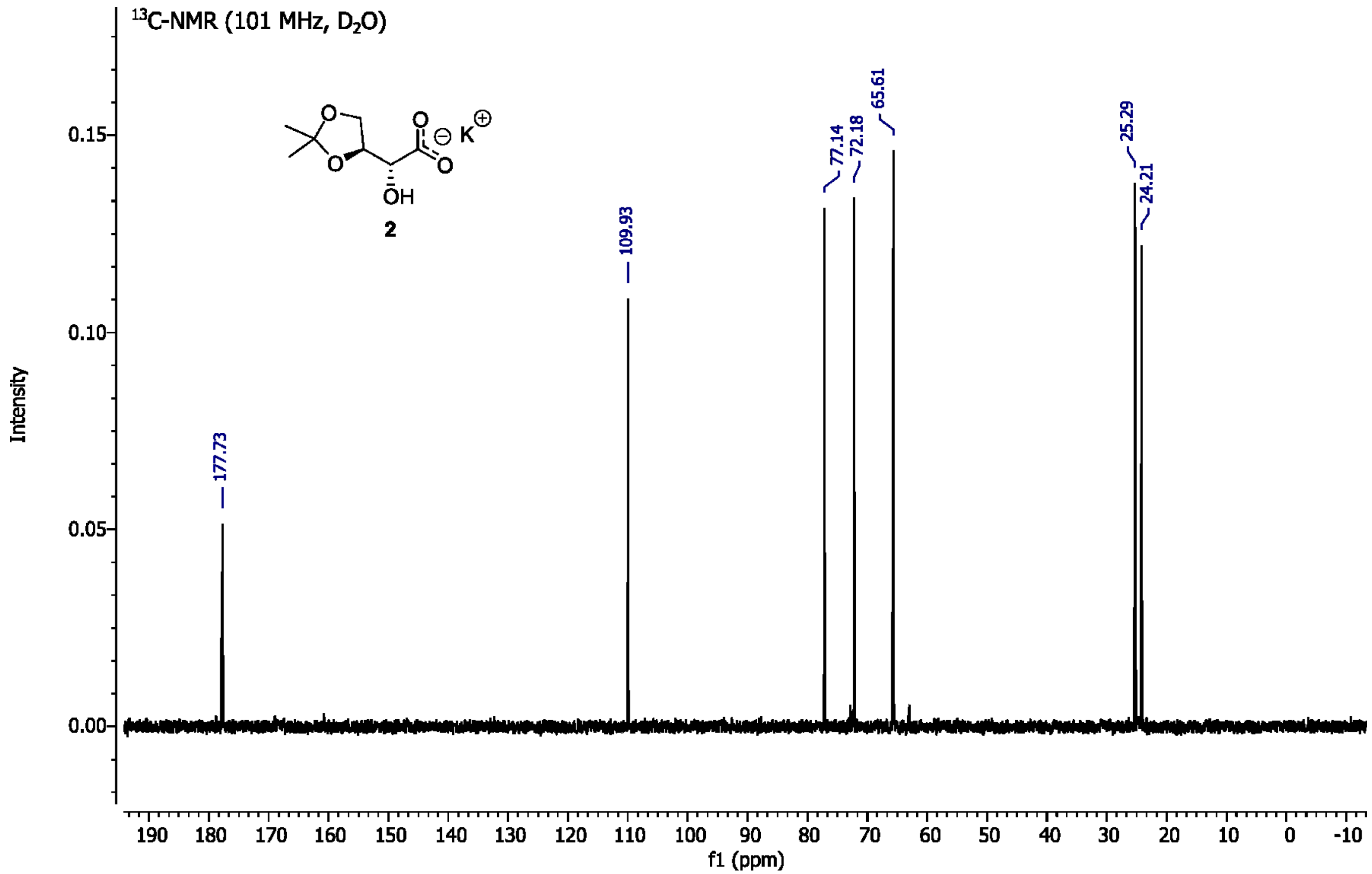

Figure S 7. ^{13}C-NMR spectrum of **2**.

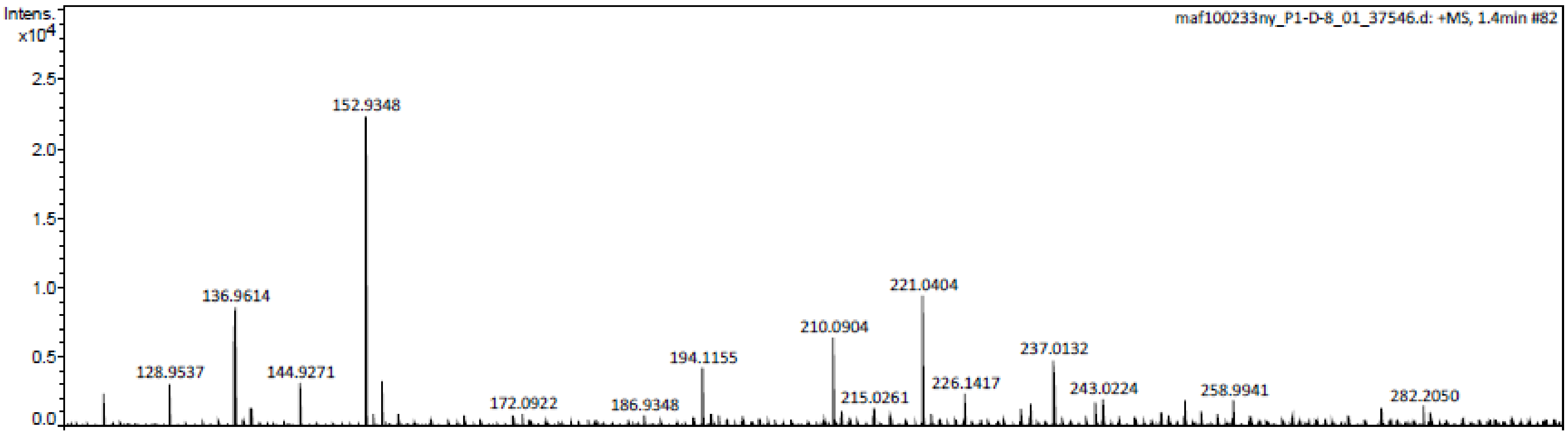

Figure S 8. (ESI)HRMS of **2**.

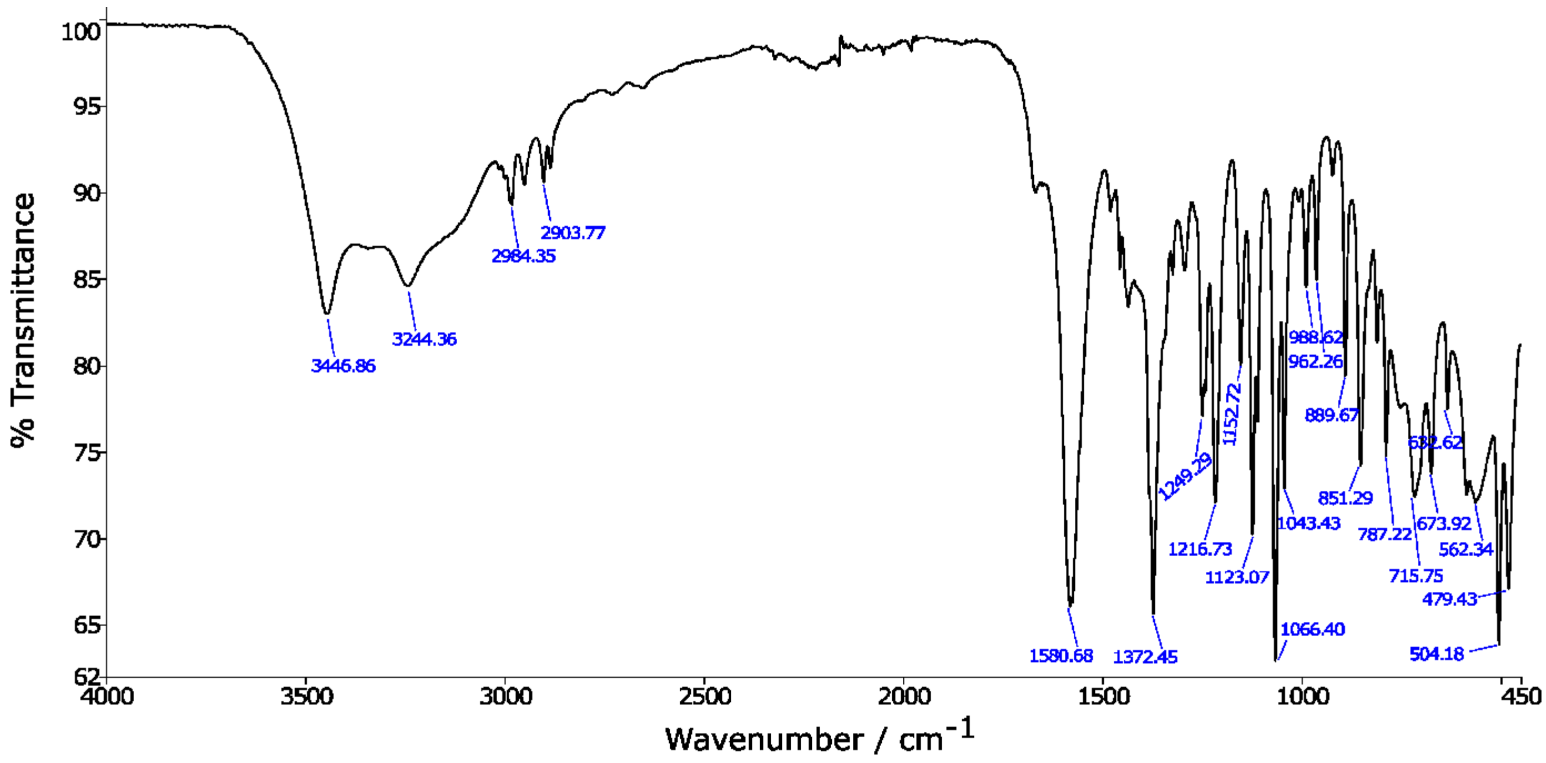

Figure S 9. FT-IR (ATR) spectrum of **2**.

Methyl 3,4-O-isopropylidene-L-threonate 3

To a suspension of **2** (8.32 g, 38.8 mmol) in MeCN (52 mL) at rt was added iodomethane (3.84 mL, 61.4 mmol). The resultant mixture was stirred at 80 °C for 3 h, after which time solids were removed via filtration. The eluate was concentrated *in vacuo* and purified via flash column chromatography (hexane → 80% EtOAc in hexane) to yield **3** as a near-colourless oil (6.73 g, 35.4 mmol, 92%).

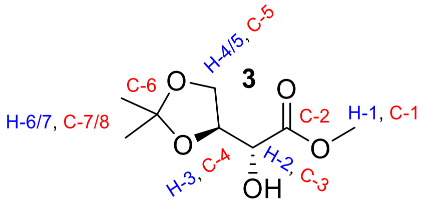

^{1}**H-NMR** (400 MHz, $CDCl_3$): δ_H 4.39 (ddd, *J* = 6.86, 6.73, 2.75 Hz, 1H, H-3), 4.12 (dd, *J* = 8.15, 2.75 Hz, 1H, H-2), 4.10 (dd, *J* = 8.33, 6.73 Hz, 1H, H-4), 4.01 (dd, *J* = 8.33, 6.86 Hz, 1H, H-5), 3.82 (s, 3H, H-1), 2.95 (d, *J* = 8.15 Hz, 1H, O<u>H</u>), 1.42 (s, 3H, H-6), 1.35 (s, 3H, H-7).

^{13}**C-NMR** (101 MHz, $CDCl_3$): δ_C 172.7 (C-2), 110.2 (C-6), 76.4 (C-4), 70.5 (C-3), 65.7 (C-5), 52.9 (C-1), 26.3 (C-7), 25.4 (C-8).

HRMS (ESI) m/z: $[M+Na]^+$ Calcd for $C_8H_{14}O_5Na$ 213.0733; Found 213.0735.

FT-IR (ATR) ($\upsilon max/cm^{-1}$): 3476 (O-H stretch, alcohol), 2989 (C-H stretch), 1740 (C=O stretch, ester), 1208 (C-O stretch, ester), 1132 (C-O stretch, alcohol), 1065.

$[\alpha]_D^{20}$: +15 (*c* 1.0, $CHCl_3$)

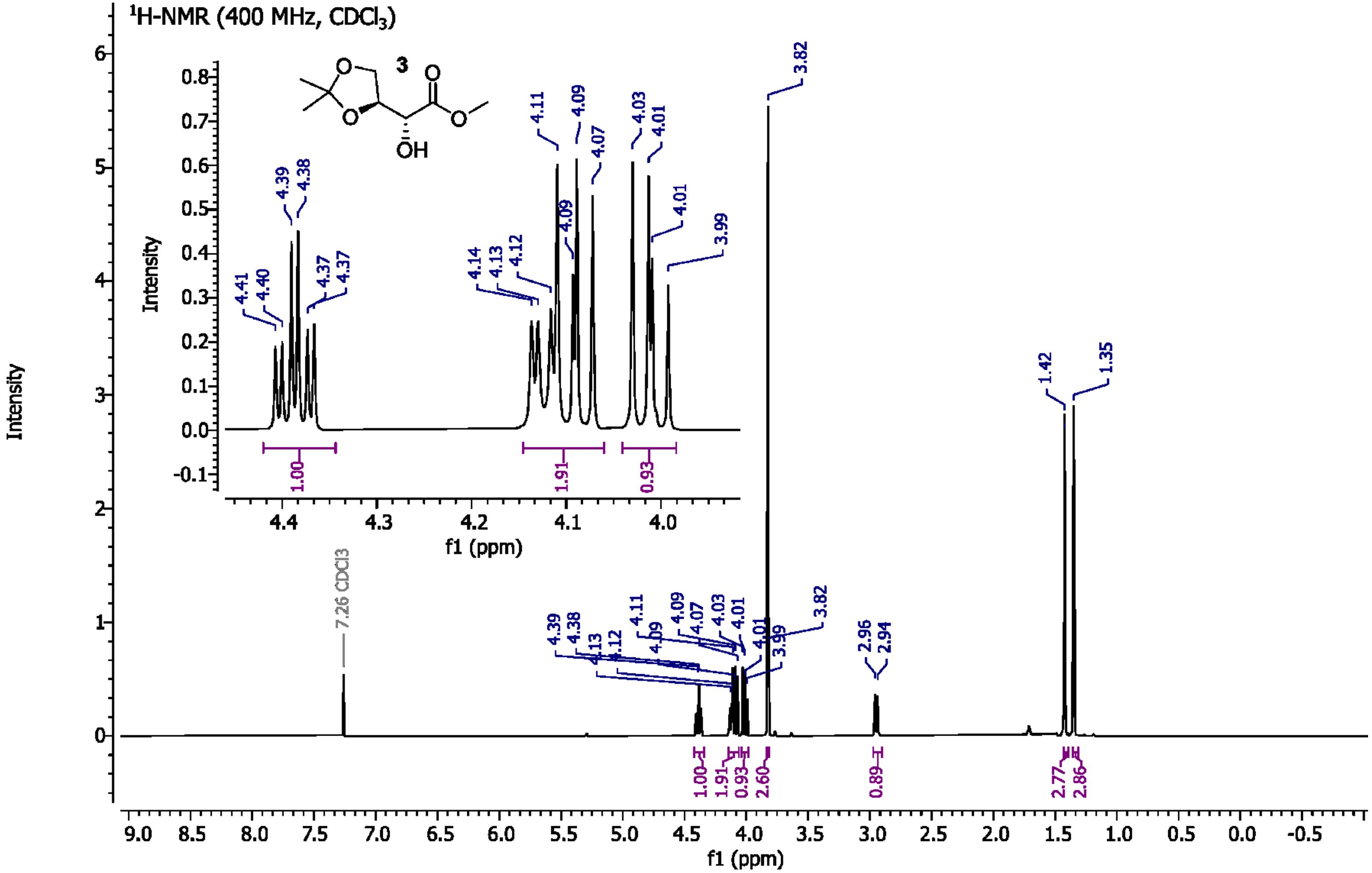

Figure S 10. ^{1}H-NMR spectrum of **3**.

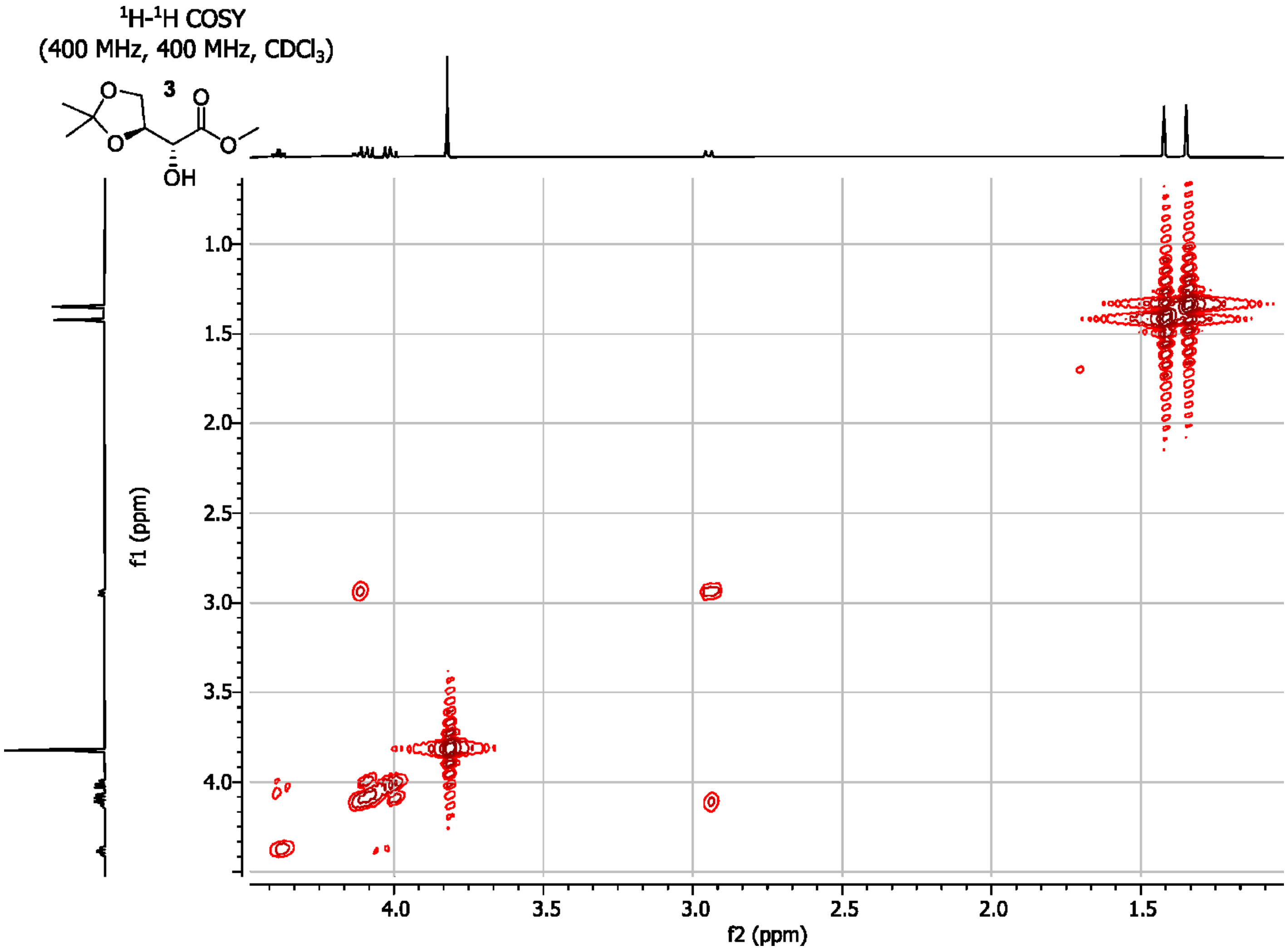

Figure S 11. ^{1}H-^{1}H COSY spectrum of **3**.

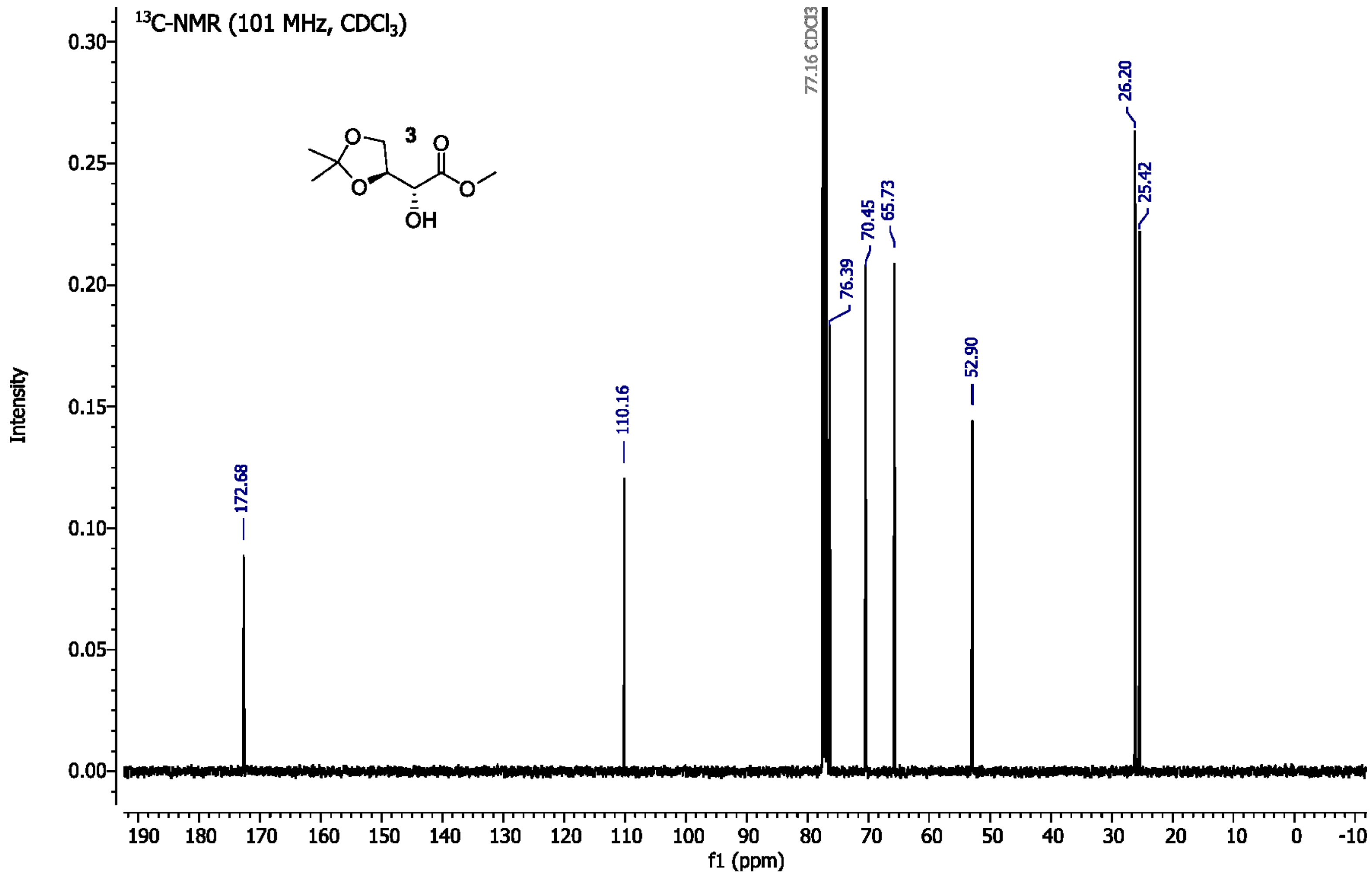

Figure S 12. ^{13}C-NMR spectrum of **3**.

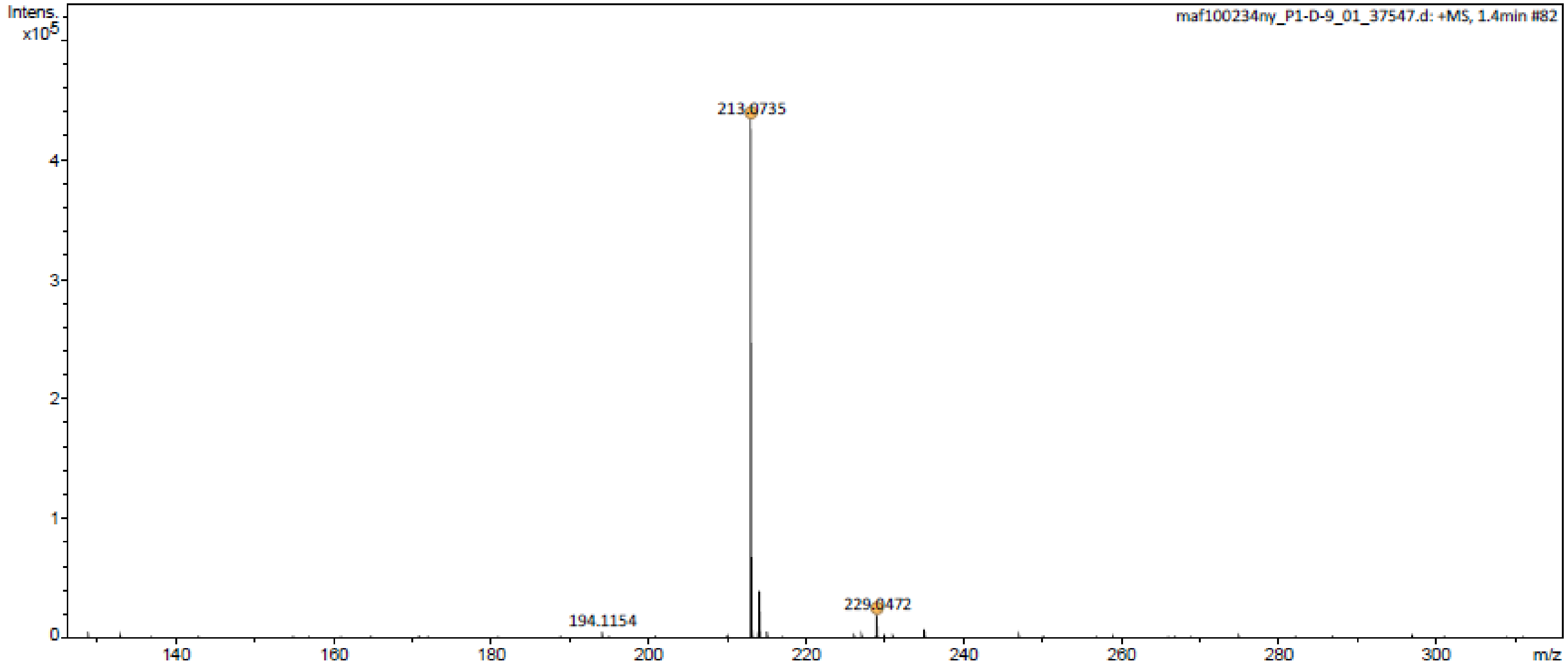

Meas. m/z	#	Ion Formula	m/z	err [ppm]	err [mDa]	mSigma	Mean err [ppm]
213.0735	1	C8H14NaO5	213.0733	-0.5	-0.1	1.5	-1.0
229.0472	1	C8H14KO5	229.0473	0.5	0.1	10.6	-1.0

Figure S 13. (ESI)HRMS of **3**.

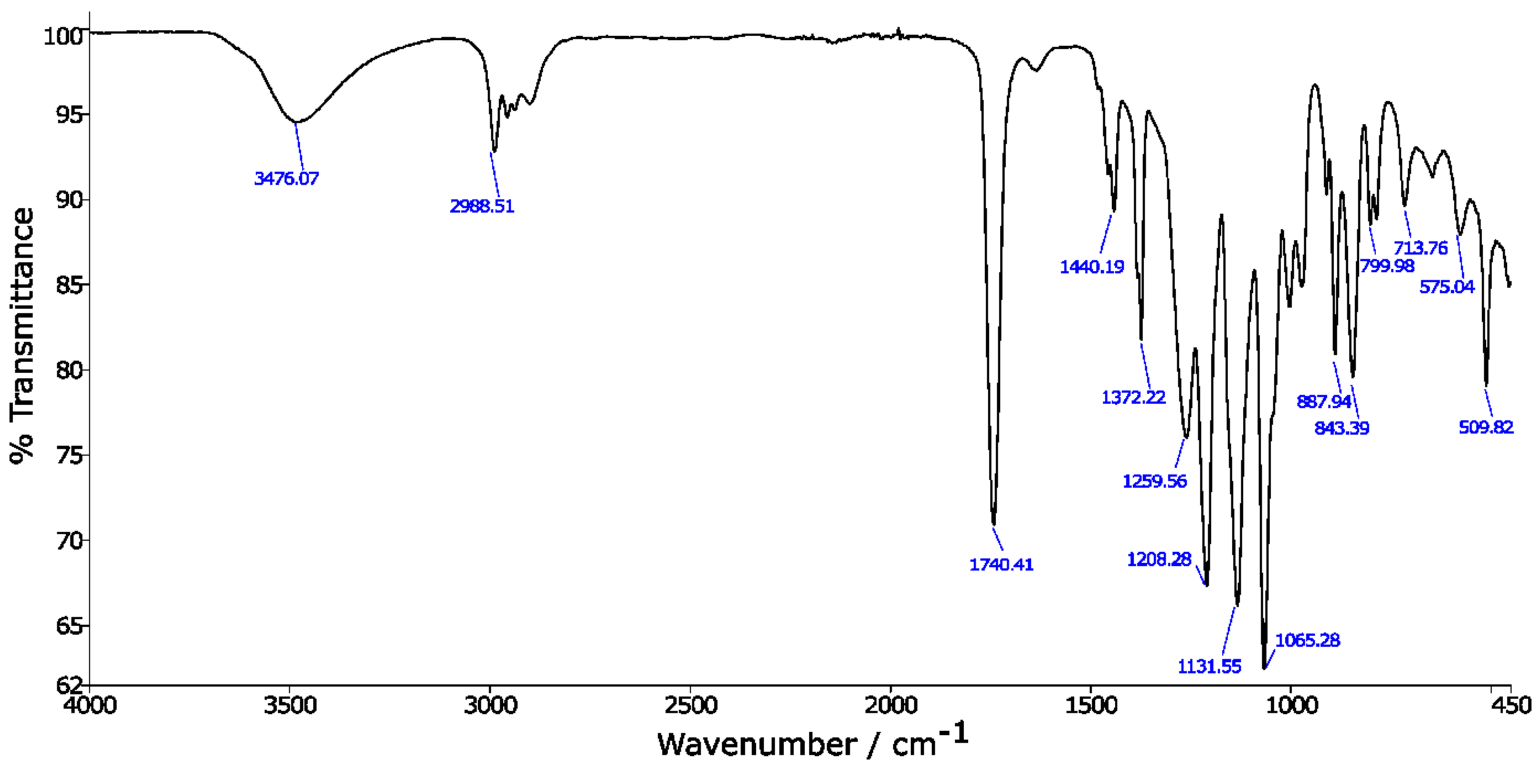

Figure S 14. FT-IR (ATR) spectrum of **3**.

(Methyl (S)-2-azido-2-((R)-2,2-dimethyl-1,3-dioxolan-4-yl)acetate 4 (via 3')

3 (26.2 g, 137 mmol) was dissolved in anhydrous DCM (250 mL) and pyridine (26 mL, 321 mmol) under N_2 and then cooled to -15 °C. Trifluoromethanesulfonic anhydride (50 g, 177 mmol) was then added dropwise over 30 minutes at -15 °C with vigorous stirring. The reaction solution was then allowed to warm to rt and stirred for a further 1 h. The reaction mixture was then transferred to a separating funnel, washed with brine (125 mL), saturated $NaHCO_3$ (125 mL), 1 M HCl (125 mL), saturated $NaHCO_3$ (125 mL) and brine (125 mL). The organic layer was then dried over $MgSO_4$ and concentrated *in vacuo* to yield a brown oil. The oil was dissolved in ethyl acetate and hexane was then added until a brown tar separated. The supernatant was then decanted, leaving behind the tar. Concentration of the supernatant via slow evaporation under a stream of nitrogen gas yielded triflate **3'** as off-white needle like crystals which were isolated via filtration and dried *in vacuo* (27.4 g, 82.2 mmol, 60%).

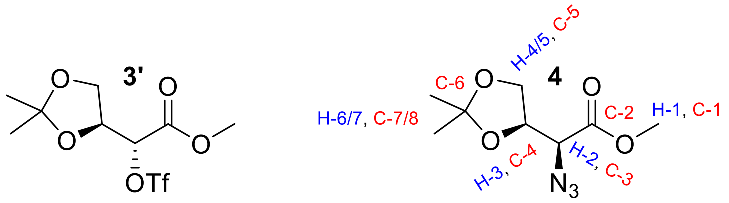

3' (27.4 g, 82.2 mmol) and sodium azide (8.30 g, 128 mmol) were then placed in a round bottom flask and 175 mL of a 3:1 v/v mixture of acetone to water was added. The resultant solution was stirred vigorously overnight at rt in the dark, during which time the solution turned a dark brown colour. The reaction solution was then transferred to a separating funnel and ethyl acetate (250 mL) and water (250 mL) added. The organic extraction was subsequently washed with water (125 mL) and brine (125 mL). The organic layer was then dried over $MgSO_4$ and concentrated in vacuo to yield a dark orange oil. This oil was purified via flash column chromatography (hexane → 80% EtOAc in hexane) to yield **4** as a colourless oil (17.0 g, 78.8 mmol, 96% from **3'**, 58% from **3**). Fractions containing **4** were identified by treating TLC plates with PPh_3 stain followed by ninhydrin stain.

^{1}H-NMR (400 MHz, $CDCl_3$): δ_H 4.47-4.41 (m, 1H, H-3), 4.08-4.04 (m, 2H, H-2,4), 3.99 (dd, *J* = 8.90, 5.06 Hz, 1H, H-5), 3.81 (s, 3H, H-1), 1.45 (s, 3H, H-6), 1.34 (s, 3H, H-7).

^{13}C-NMR (100 MHz, $CDCl_3$): δ_C 168.5 (C-2), 110.5 (C-6), 75.4 (C-4), 65.8 (C-5), 63.4 (C-3), 53.0 (C-1), 26.4 (C-7), 25.1 (C-8).

HRMS (ESI) m/z: $[M+Na]^+$ Calcd for $C_8H_{13}N_3O_4Na$ 238.0798; Found 238.0799.

FT-IR (ATR) (υmax/cm^{-1}): 2989 (C-H stretch), 2109 (N=N=N stretch, azide), 1743 (C=O stretch, ester), 1204 (C-O stretch, ester), 1060.

$[\alpha]_D^{20}$: -9 (*c* 1.0, $CHCl_3$)

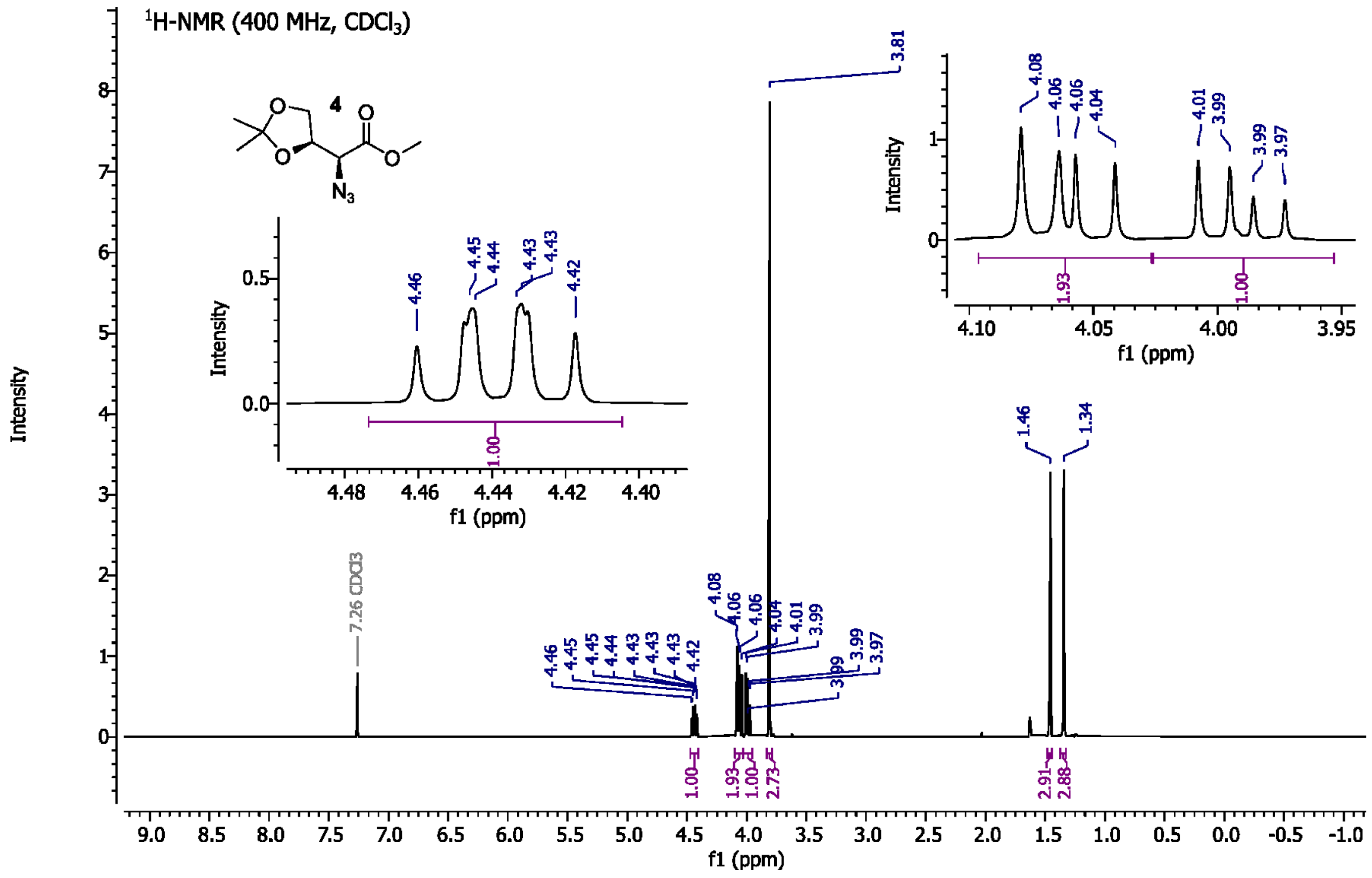

Figure S 15. ^{1}H-NMR spectrum of **4**.

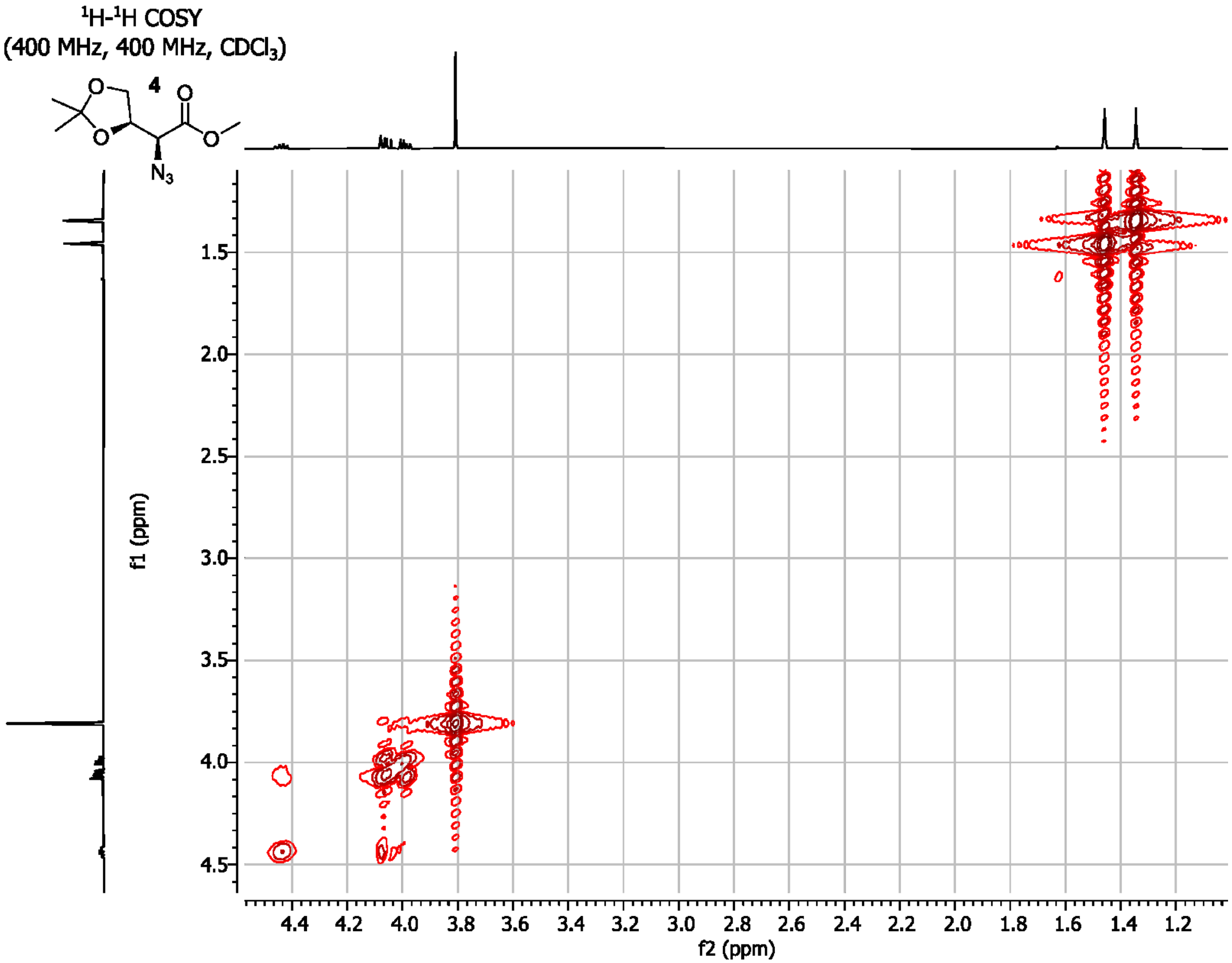

Figure S 16. ^{1}H-^{1}H COSY spectrum of **4**.

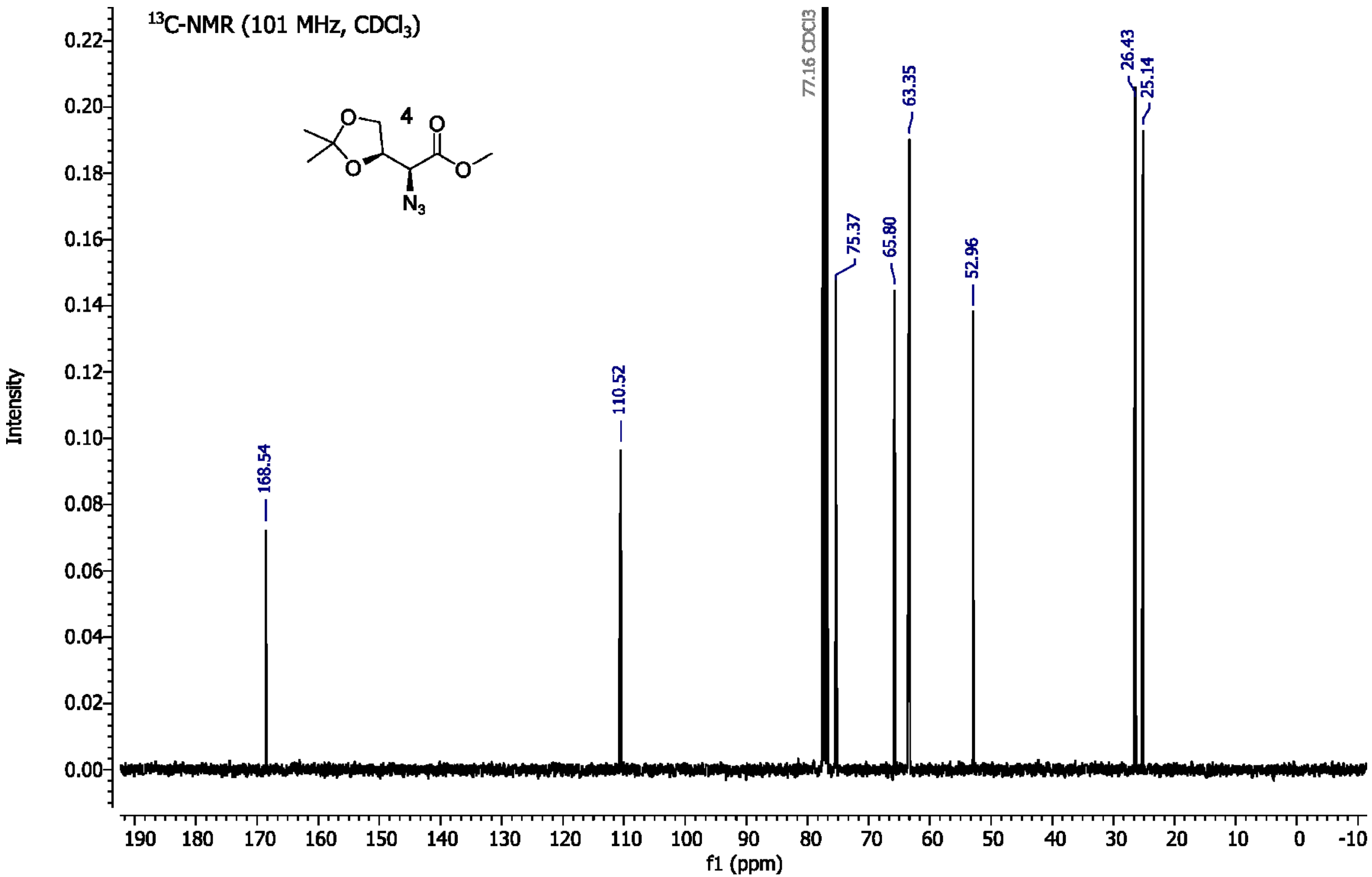

Figure S 17. ^{13}C-NMR spectrum of **4**.

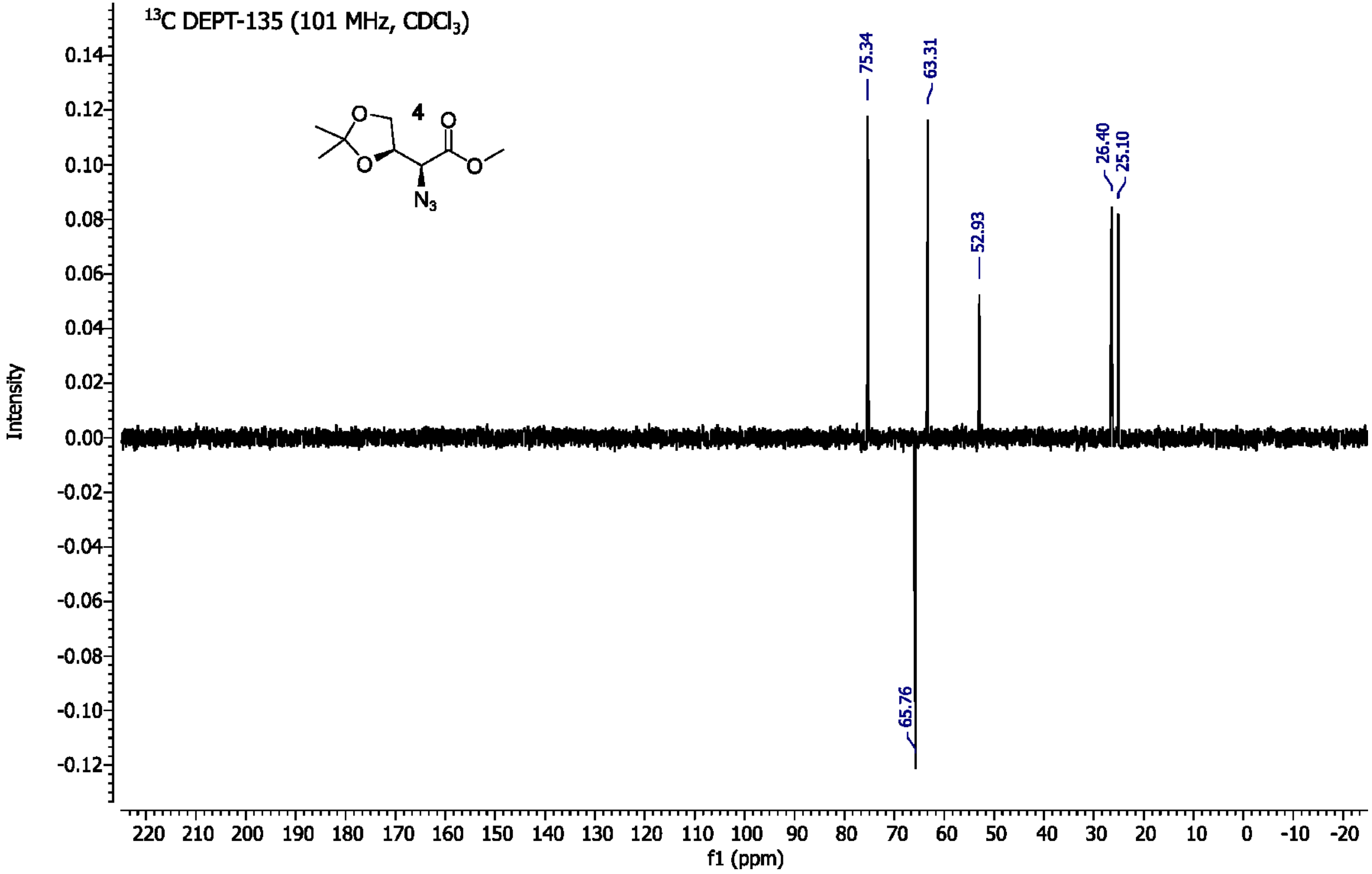

Figure S 18. ^{13}C DEPT-135 spectrum of **4**.

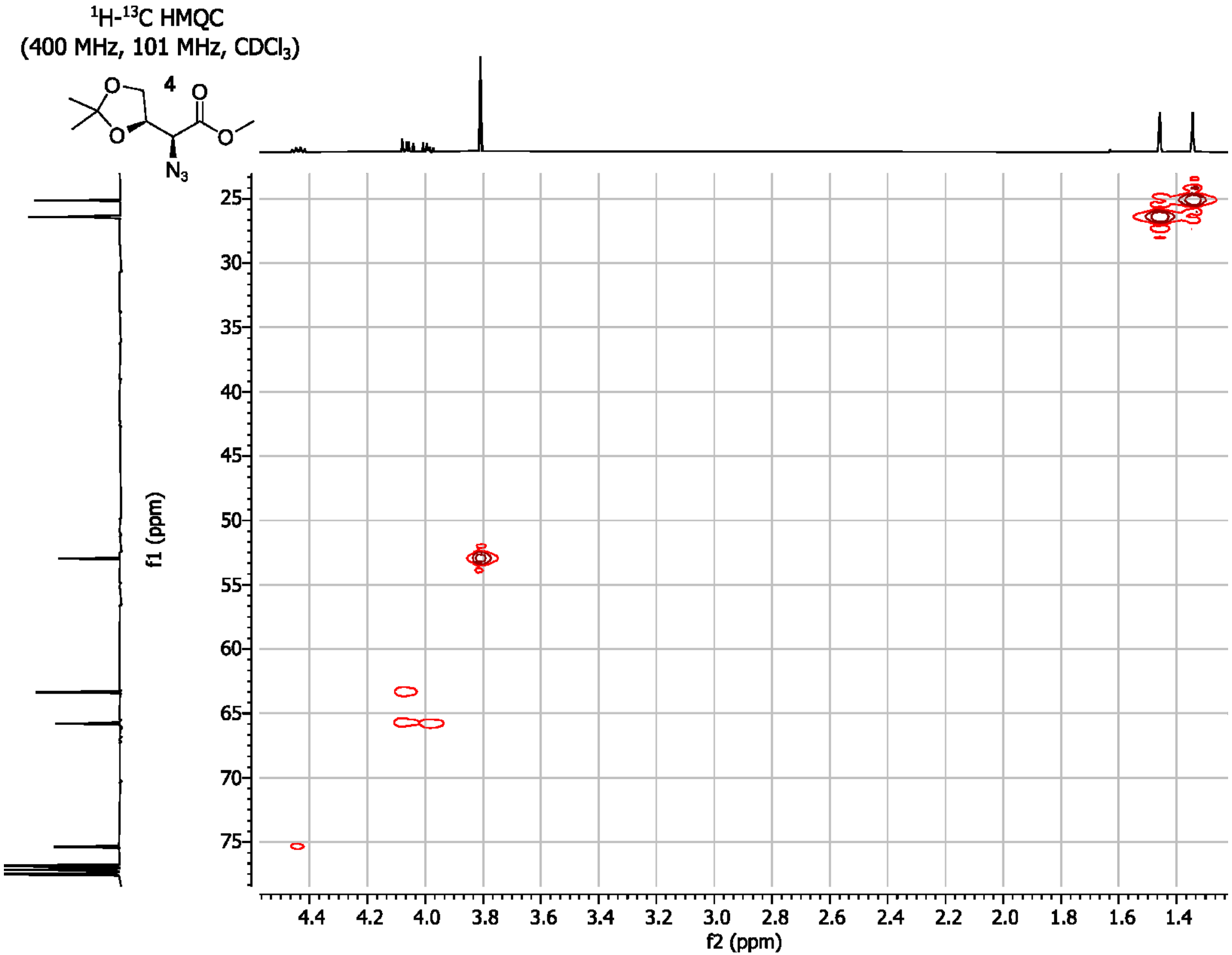

Figure S 19. ^{1}H-^{13}C HMQC spectrum of **4**.

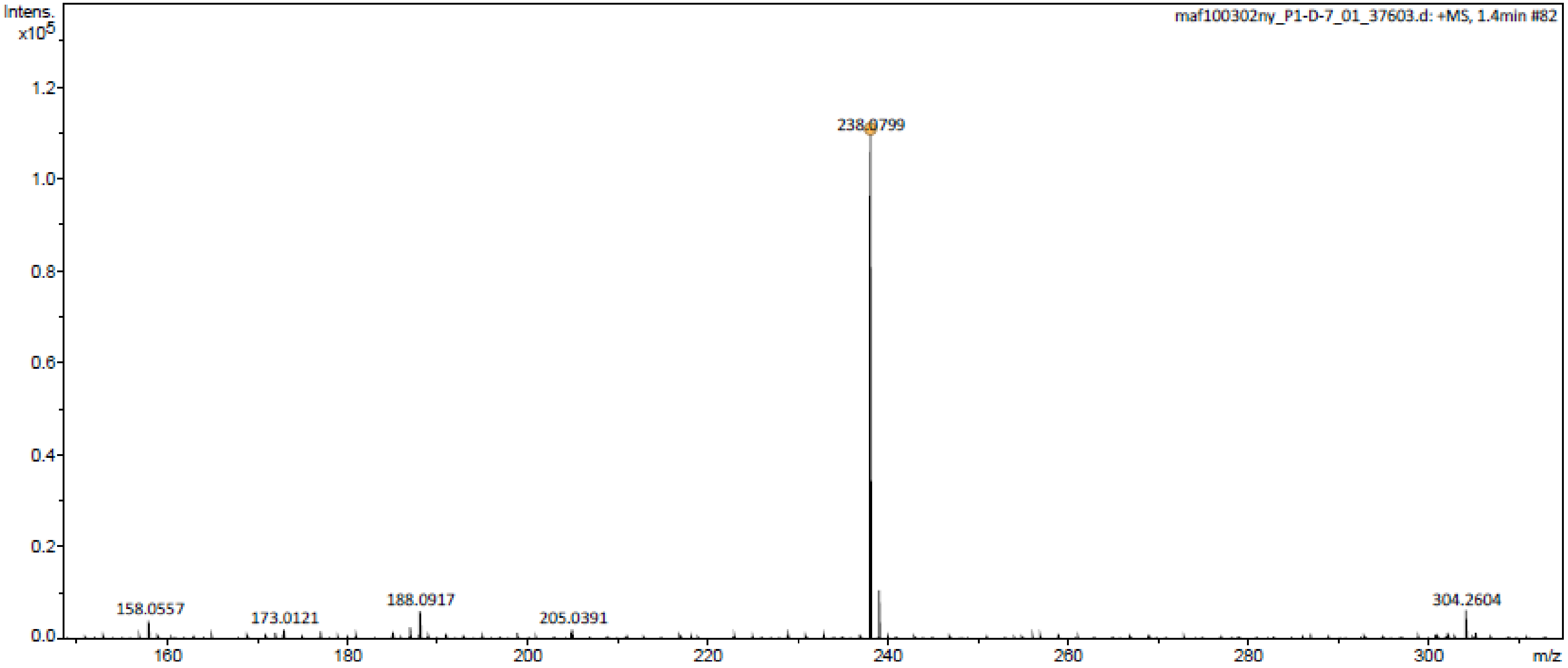

Meas. m/z	#	Ion Formula	m/z	err [ppm]	err [mDa]	mSigma	Mean err [ppm]
238.0799	1	C8H13N3NaO4	238.0798	-0.4	-0.1	4.4	-3.7

Figure S 20. (ESI)HRMS of **4**.

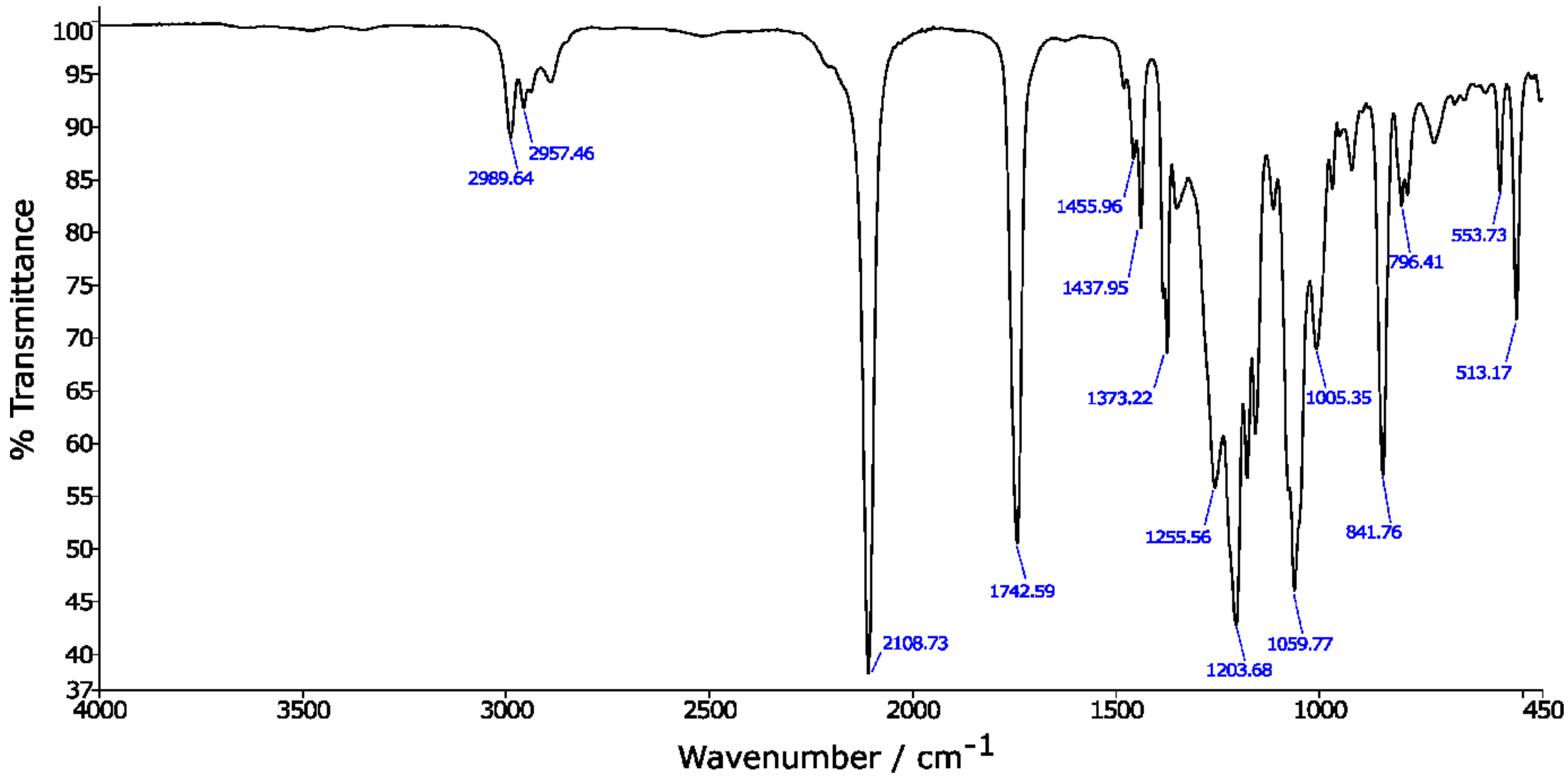

Figure S 21. FT-IR (ATR) spectrum of **4**.

Methyl (2S)-[(4R)-2,2-dimethyl-1,3-dioxolan-4-yl]({[(9H-fluoren-9-yl)methoxy]carbonyl}amino) acetate 5

Note that two methods are reported below. **Method 2** was higher yielding, but the product was easier to purify when using **Method 1**.

Method 1: To **4** (6.00 g, 27.9 mmol) dissolved in MeOH (100 mL) was added a heaped spatula of 10% palladium on activated carbon. The system was then placed under 1 atm of H_2. The resultant mixture was stirred at rt until azide reduction was complete by TLC (approx. 2 h). Azides and amines were visualised on the TLC plates using PPh_3 and ninhydrin stains. The Pd/C was then removed via filtration and 9-fluorenylmethyl *N*-succinimidyl carbonate (11.3 g, 33.5 mmol) was added to the eluate to yield a suspension. DCM (100 mL) was then added to aid solvation. The reaction solution was then stirred overnight at rt, concentrated *in vacuo*, resuspended in EtOAc (200 mL) and washed with water (100 mL) and brine (100 mL). The organic extract was then dried over $MgSO_4$ and concentrated *in vacuo* to yield a yellow oil that was purified via flash column chromatography (hexane → EtOAc) to yield **5** (4.43 g, 10.8 mmol, 39%) as an off-colourless oil that solidified into an off-white solid.

Method 2: To **4** (1.00 g, 4.64 mmol) dissolved in THF (47.5 mL) was added water (2.5 mL). PPh_3 (2.44 g, 9.28 mmol) was then added and the resultant solution stirred for 2 h at rt. 9-fluorenylmethyl *N*-succinimidyl carbonate (1.9 g, 5.57 mmol), $NaHCO_3$ (0.585 g, 6.96 mmol) and water (2.5 mL) were then added and the reaction solution was stirred overnight at rt. After this time the solution was concentrated *in vacuo*, resuspended in EtOAc (200 mL) and washed with water (100 mL) and brine (100 mL). The organic extract was then dried over $MgSO_4$ and concentrated *in vacuo* to yield a crude residue. This residue was dissolved in the minimum volume of hot EtOAc and was then cooled using an ice-bath, prompting the precipitation of PPh_3O. The PPh_3O was removed via filtration and the eluate was purified via flash column chromatography (hexane → EtOAc) to yield **5** (0.998 g, 2.43 mmol, 52%) as an off-colourless oil that solidified into an off-white solid.

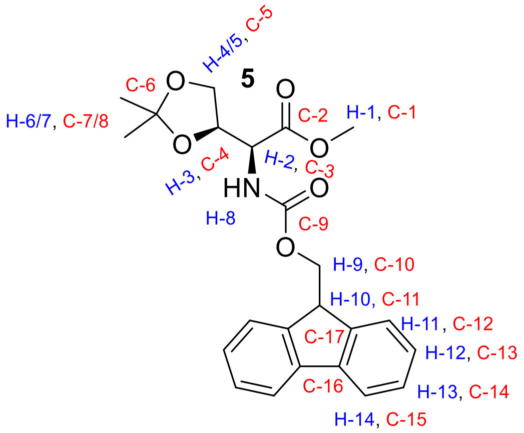

^{1}H-NMR (400 MHz, $CDCl_3$): δ_H 7.79-7.74 (m, 2H, H-14), 7.63-7.57 (m, 2H, H-11), 7.44-7.37 (m, 2H, H-13), 7.35-7.29 (m, 2H, H-12), 5.58 (d, *J* = 8.70 Hz, 1H, H-8), 4.48 (dd, *J* = 8.70 Hz, 4.26, 1H, H-2), 4.45-4.33 (m, 3H, H-3,H-9), 4.22 (t, *J* = 6.97 Hz, H-10), 4.15-4.03 (m, 2H, H-4, H-5), 3.79 (s, 3H, H-1), 1.40 (s, 3H, H-6), 1.33 (s, 3H, H-7).

^{13}C-NMR (100 MHz, $CDCl_3$): δ_C 170.3 (C-2), 156.0 (C-9), 143.8 (C-17), 141.5 (C-16), 127.9 (C-14), 127.2 (C-13), 125.2 (C-12), 120.2 (C-15), 110.4 (C-6), 76.4 (C-4), 67.4 (C-10), 65.9 (C-5), 56.4 (C-3), 52.8 (C-1), 47.3 (C-11), 26.5 (C-7), 25.0 (C-8).

HRMS (ESI) m/z: $[M+Na]^+$ Calcd for $C_{23}H_{25}NO_6Na$ 434.1574; Found 434.1574.

FT-IR (ATR) (υmax/cm^{-1}): 3323 (N-H stretch, carbamate), 2989 (C-H stretch), 2952 (C-H stretch), 1717 (C=O stretches), 1207 (C-O stretch, ester), 1055.

$[\alpha]_D^{20}$: +26 (*c* 1.0, $CHCl_3$)

mp: 97 °C

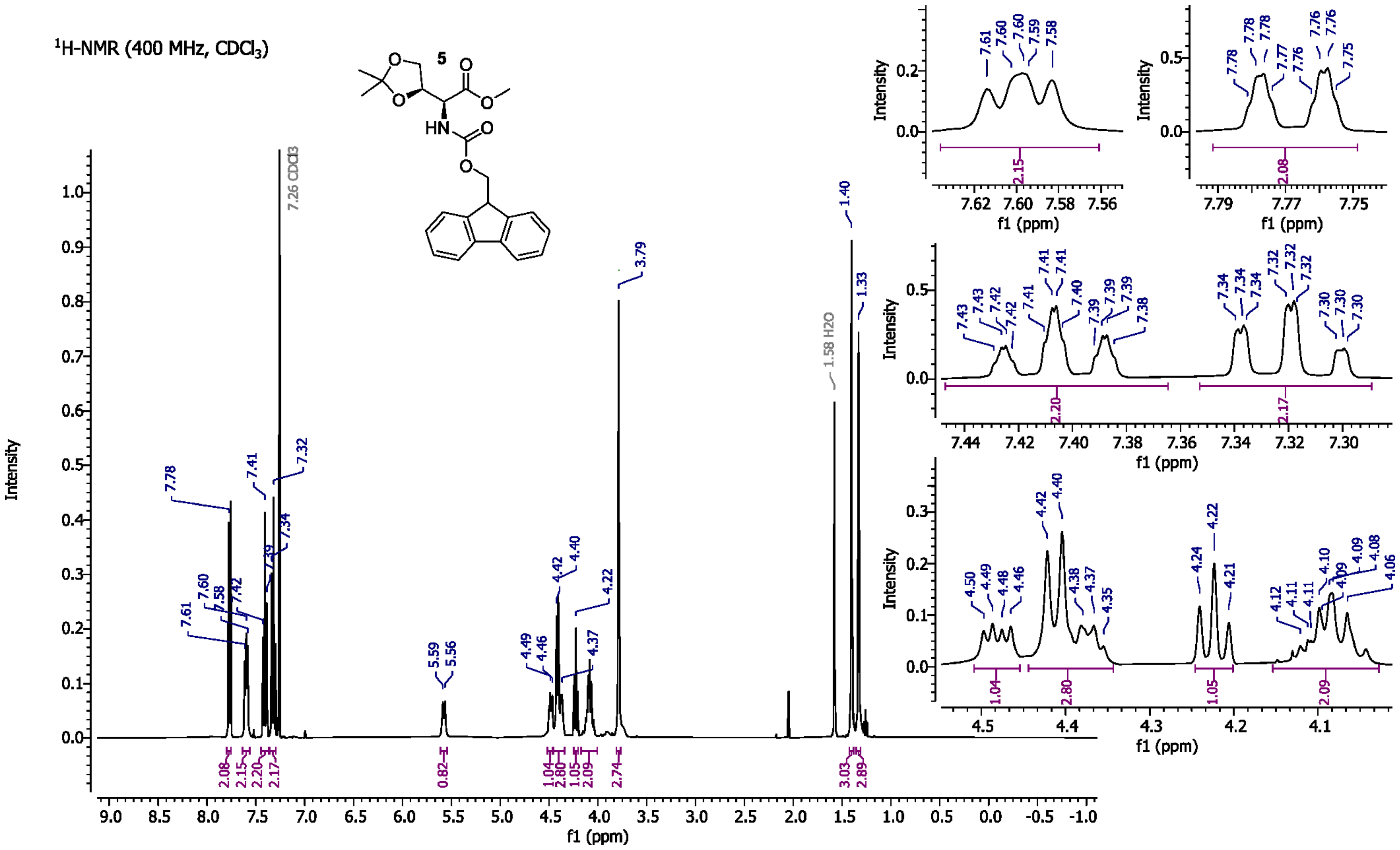

Figure S 22. ^{1}H-NMR spectrum of **5**.

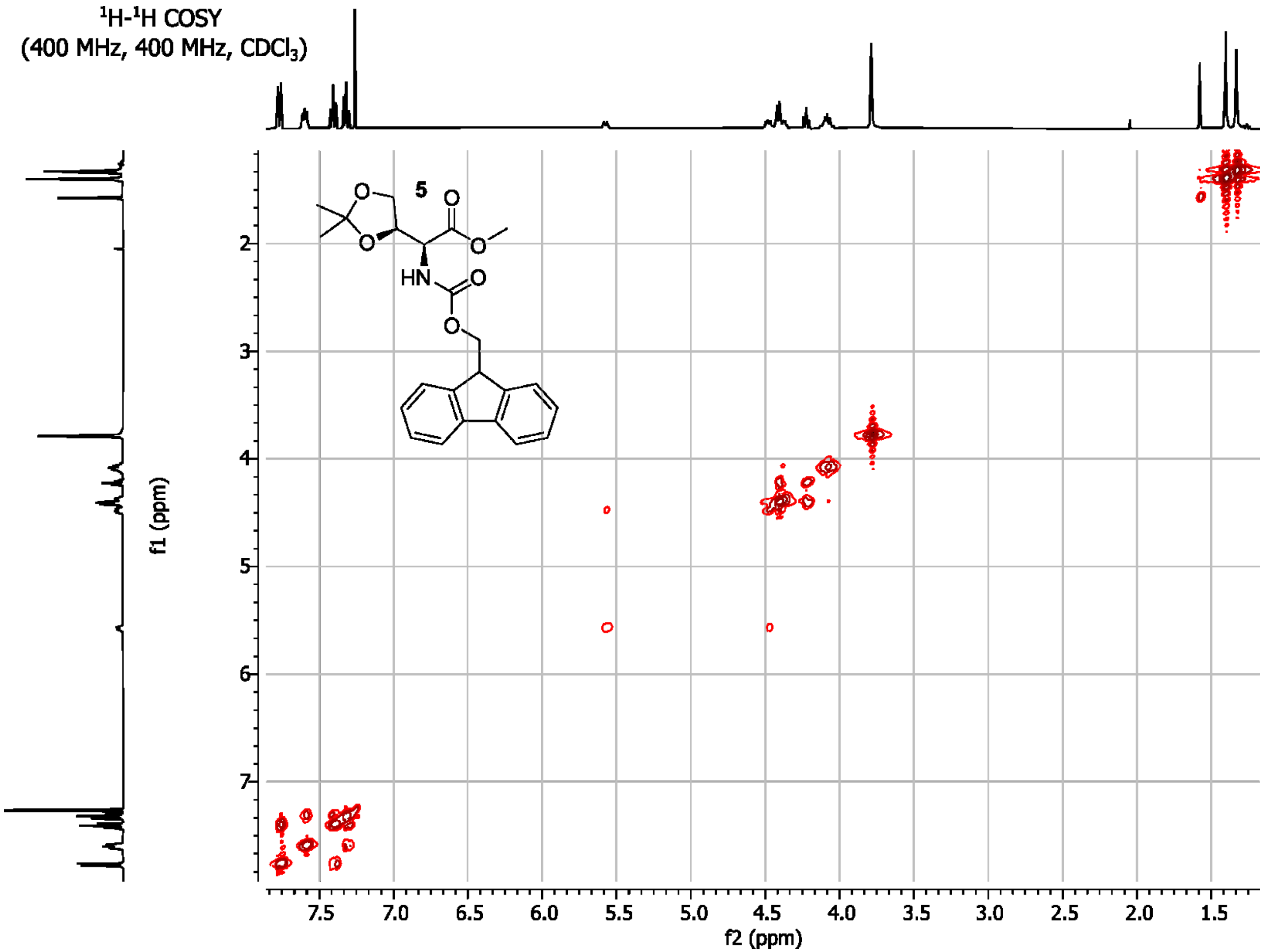

Figure S 23. ^{1}H-^{1}H COSY spectrum of **5**.

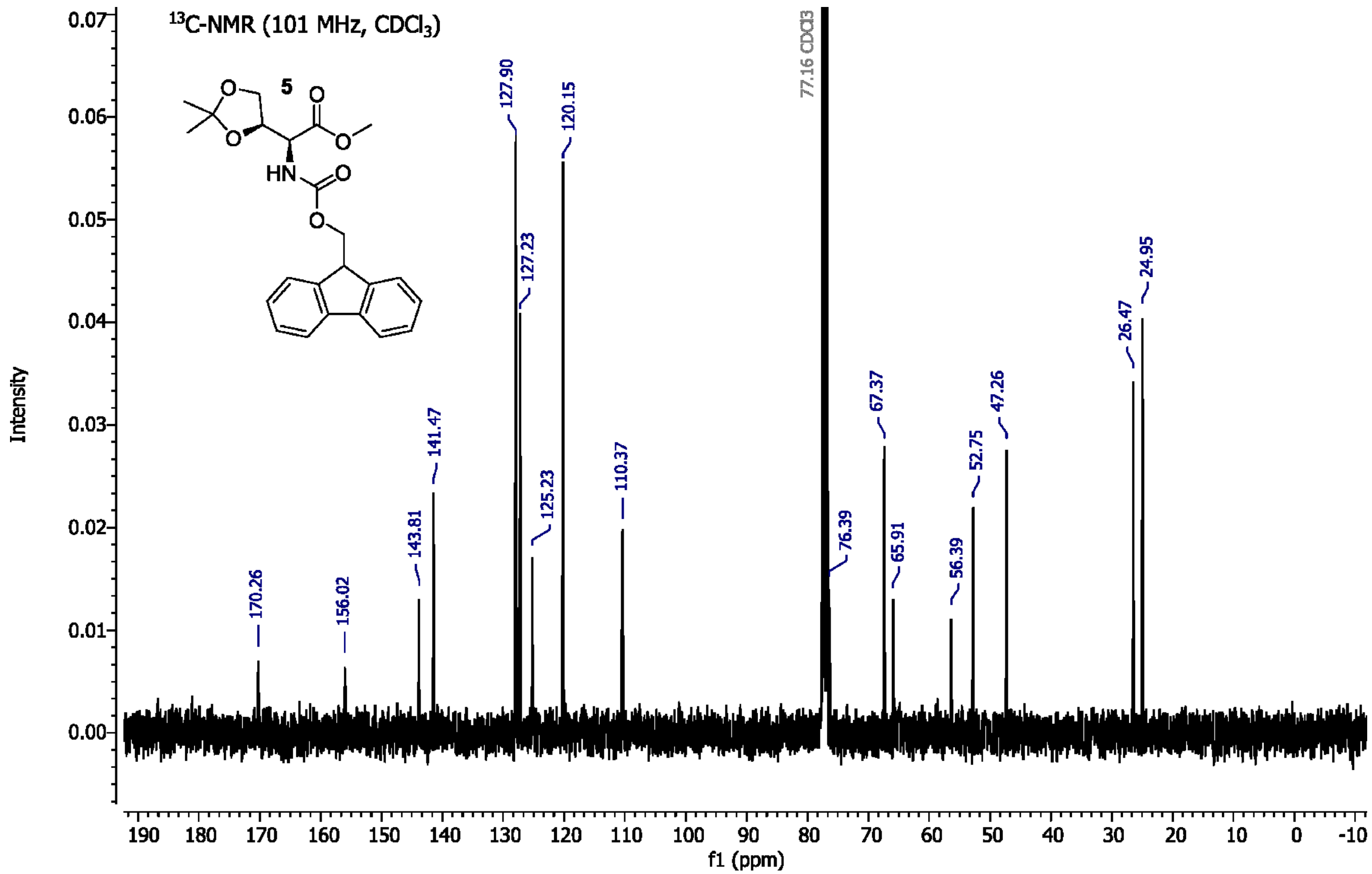

Figure S 24. ^{13}C-NMR spectrum of **5**.

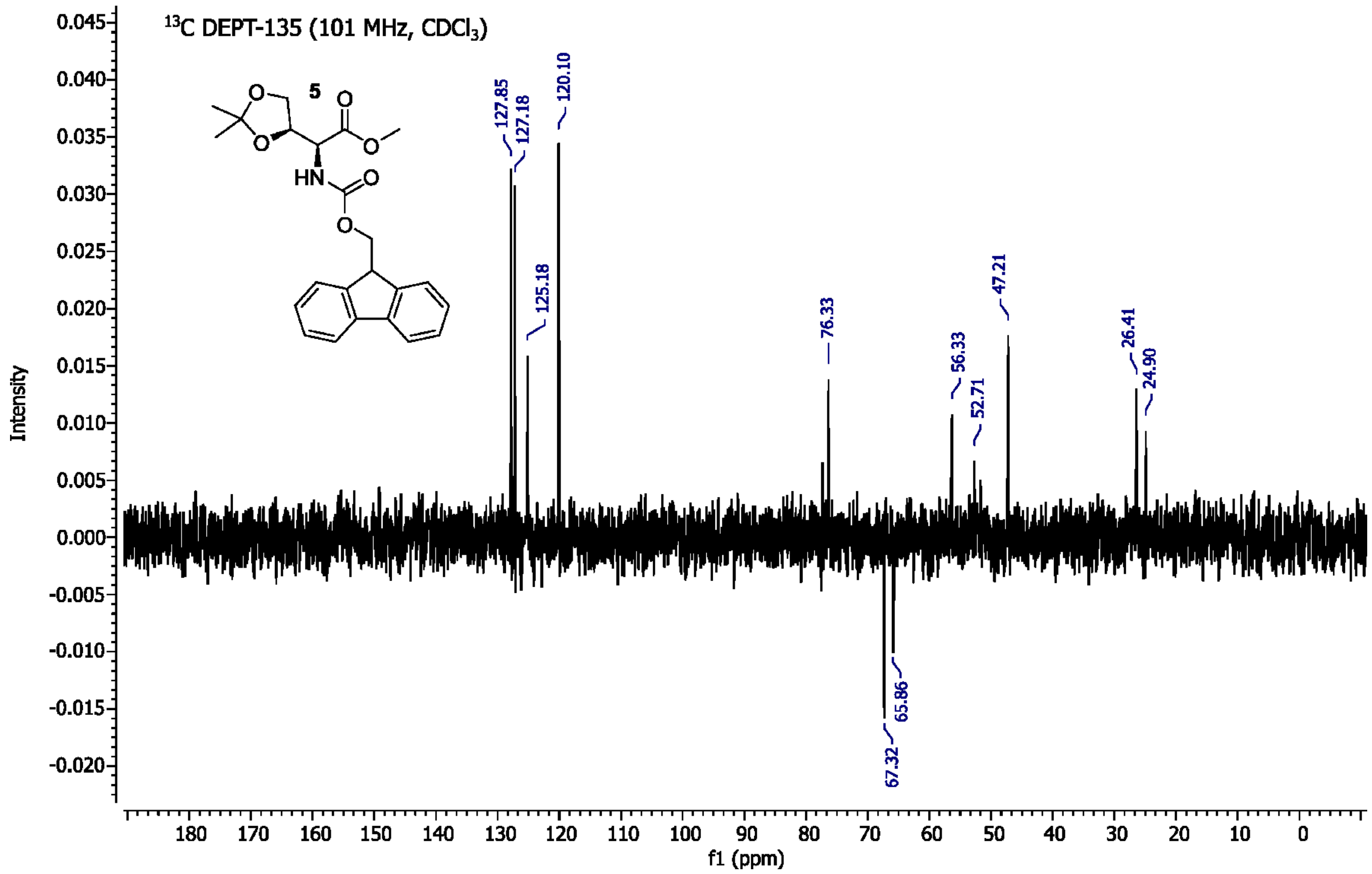

Figure S 25. ^{13}C DEPT-135 spectrum of **5**.

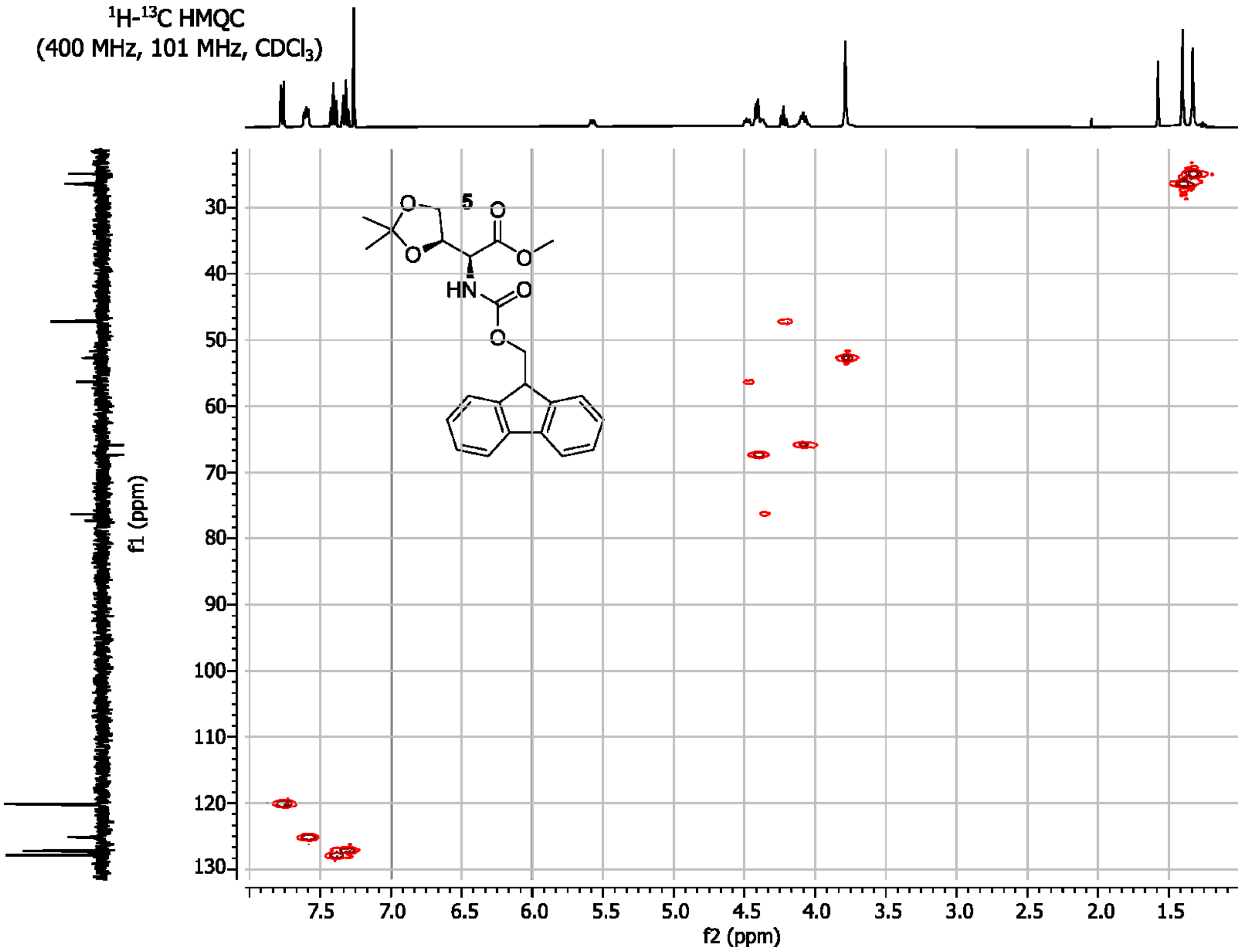

Figure S 26. ^{1}H-^{13}C HMQC spectrum of **5**.

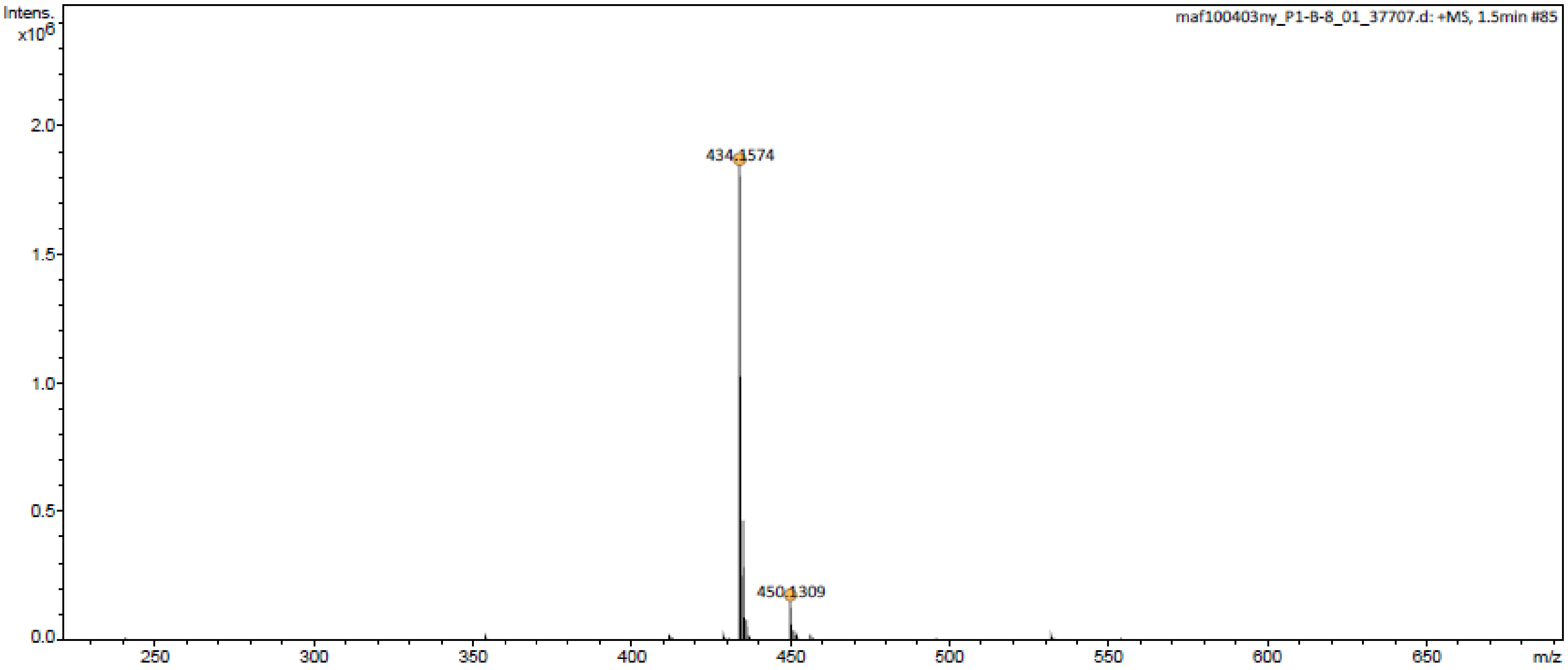

Meas. m/z	#	Ion Formula	m/z	err [ppm]	err [mDa]	mSigma	Mean err [ppm]
434.1574	1	C23H25NNaO6	434.1574	-0.0	-0.0	2.4	-0.0
450.1309	1	C23H25KNO6	450.1313	1.0	0.5	4.8	-0.4

Figure S 27. (ESI)HRMS of **5**.

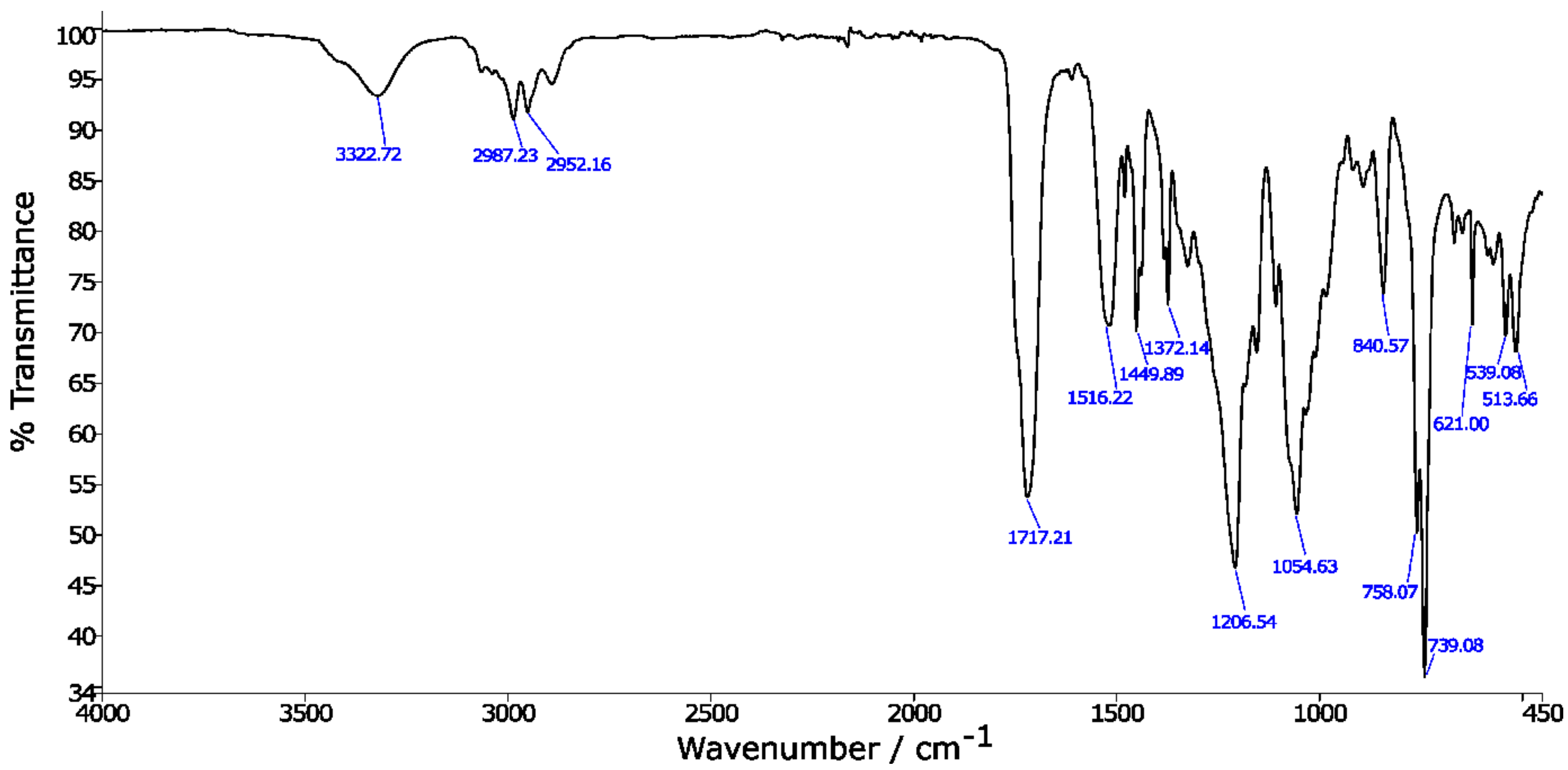

Figure S 28. FT-IR (ATR) spectrum of **5**.

(2S)-[(4R)-2,2-dimethyl-1,3-dioxolan-4-yl]({[(9H-fluoren-9-yl)methoxy]carbonyl}amino)acetic acid 6

$CaCl_2$ (25.0 g, 226 mmol) was dissolved with stirring in a 7:3 v/v 2-propanol:water mixture (290 mL). 13.2 mL of 1 M NaOH was then added and the resultant cloudy mixture was cooled to 0 °C. **5** (3.64 g, 8.84 mmol) was dissolved in the minimum volume of DCM and the $CaCl_2$/NaOH solution was then added in one portion. The reaction solution was then stirred rt until TLC showed the reaction to have reached completion (approx. 4 hours). The reaction solution was then transferred into a separating funnel and DCM (250 mL) and brine (250 mL) were added. The aqueous layer was acidified to pH 5 via the dropwise addition of 1 M HCl and the biphasic system shaken to extract the product into the organic layer. The aqueous layer was then further extracted with DCM (2 × 100 mL). All organic extracts were then combined and dried over $MgSO_4$ before being concentrated *in vacuo*. This yielded a pale green oil which was purified via flash column chromatography (25% EtOAc in hexane + 0.5% AcOH → EtOAc + 0.5%) to yield **6** (2.95 g, 7.43 mmol, 84%) as a colourless oil that solidified into a white solid.

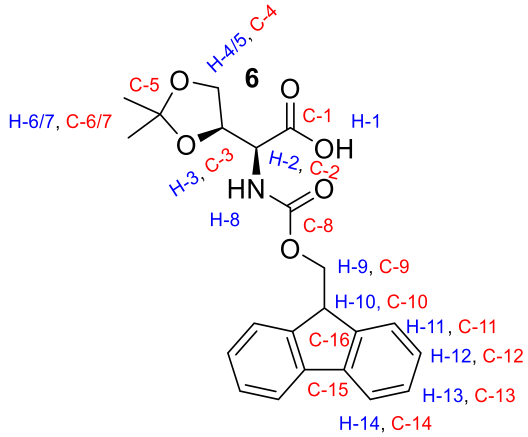

^{1}H-NMR (400 MHz, $CDCl_3$): δ_H 7.79-7.73 (m, 2H, H-14), 7.63-7.55 (m, 2H, H-11), 7.43-7.36 (m, 2H, H-13), 7.34-7.28 (m, 2H, H-12), 6.61 (br s, 1H, H-1), 5.65 (d, *J* = 8.34 Hz, 1H, H-8), 4.50 (dd, *J* = 8.34 Hz, 4.76, 1H, H-2), 4.45-4.38 (m, 2H, H-9), 4.22 (t, *J* = 6.76 Hz, H-10), 4.16-4.04 (m, 2H, H-4/5), 1.43 (s, 3H, H-6), 1.34 (s, 3H, H-7).

^{13}C-NMR (100 MHz, $CDCl_3$): δ_C 173.4 (C-1, 156.4 (C-2), 143.7 (C-16), 141.5 (C-15), 127.9 (C-13), 127.2 (C-12), 125.2 (C-11), 120.2 (C-14), 110.7 (C-5), 75.9 (C-3), 67.5 (C-9), 65.9 (C-4), 56.2 (C-2), 47.2 (C-10), 26.4 (C-6), 24.9 (C-7).

HRMS (ESI) m/z: $[M-H]^-$ Calcd for $C_{22}H_{22}NO_6$ 396.1453; Found 396.1457.

FT-IR (ATR) (υmax/cm^{-1}): 3310 (N-H stretch, carbamate), 3300-2750 (O-H stretch, carboxylic acid), 2987 (C-H stretch), 2937 (C-H stretch), 1710 (C=O stretches), 1213 (C-O stretch), 1056.

$[\alpha]_D^{20}$: +16 (*c* 1.0, $CHCl_3$)

mp: 83 °C

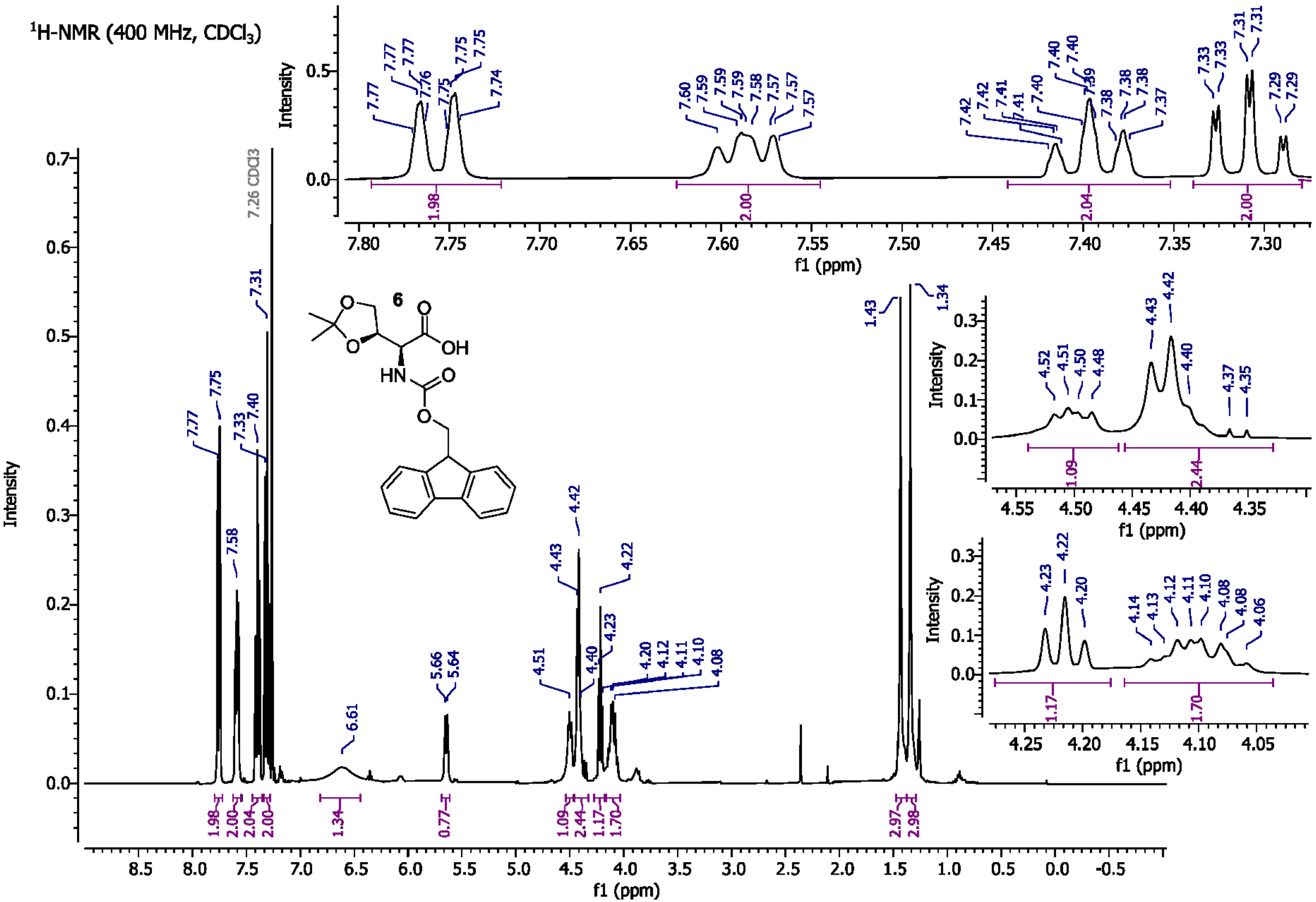

Figure S 29. ^{1}H-NMR spectrum of **6**.

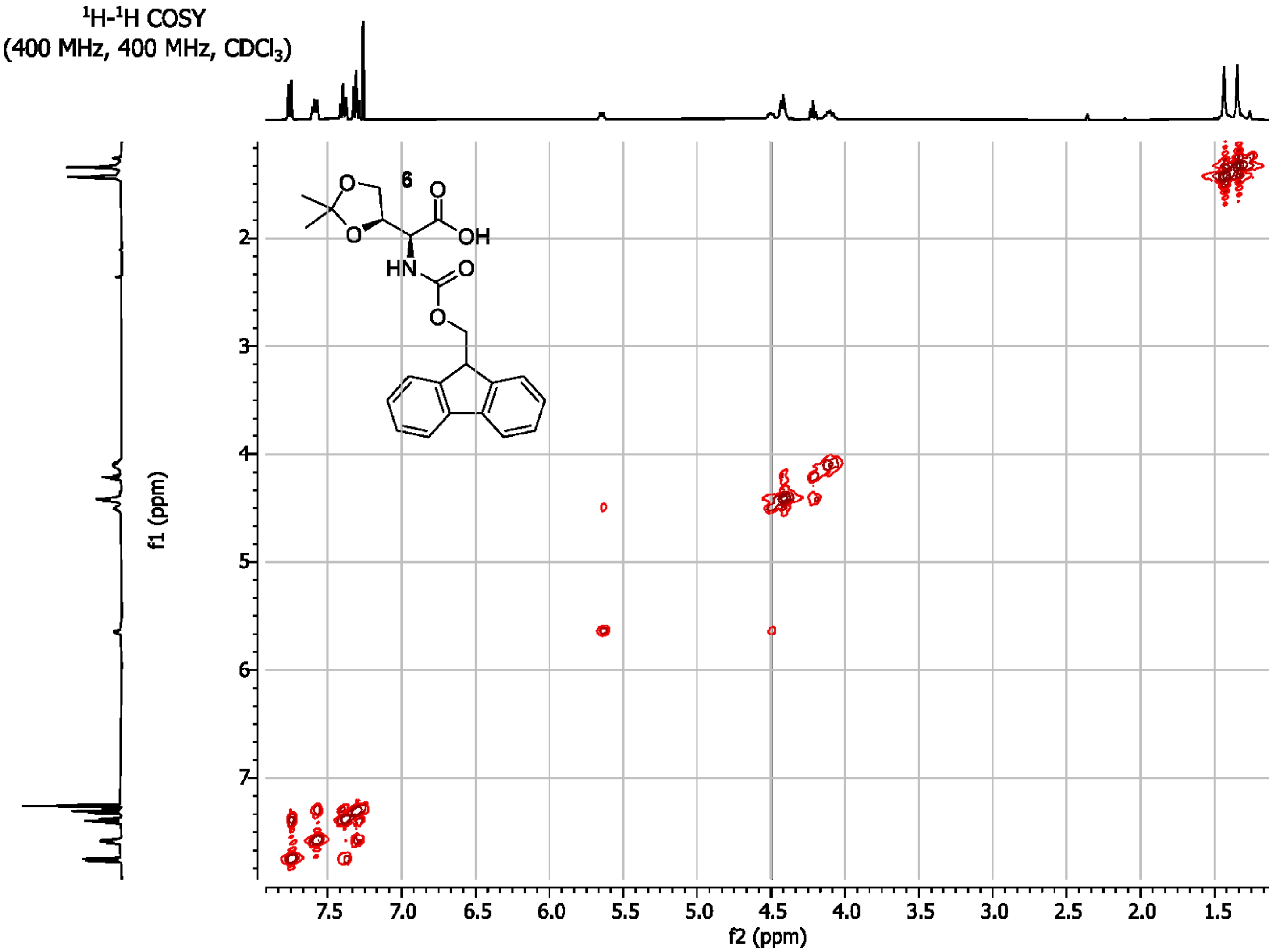

Figure S 30. ¹H-¹H COSY spectrum of **6**.

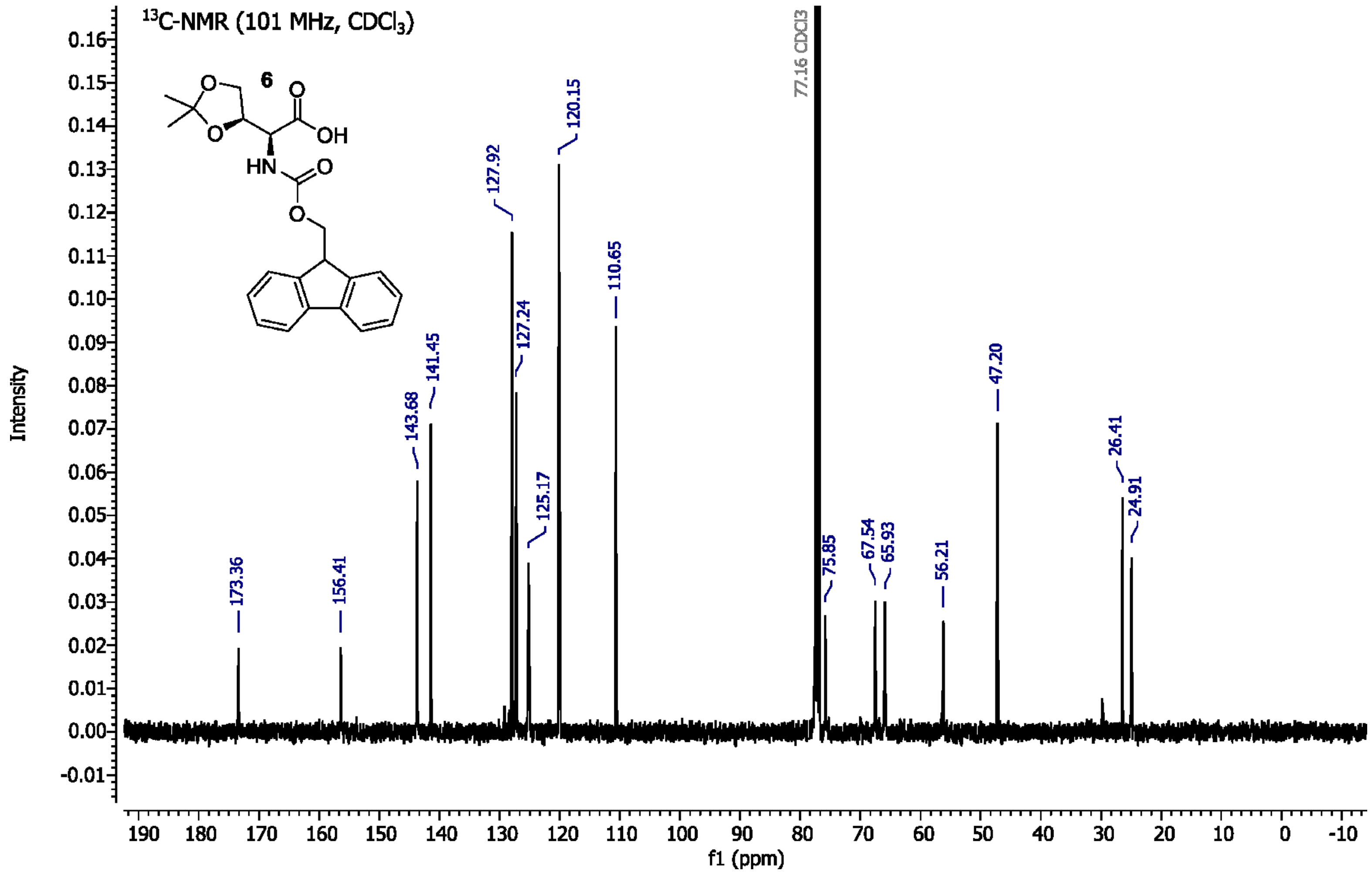

Figure S 31. ^{13}C-NMR spectrum of **6**.

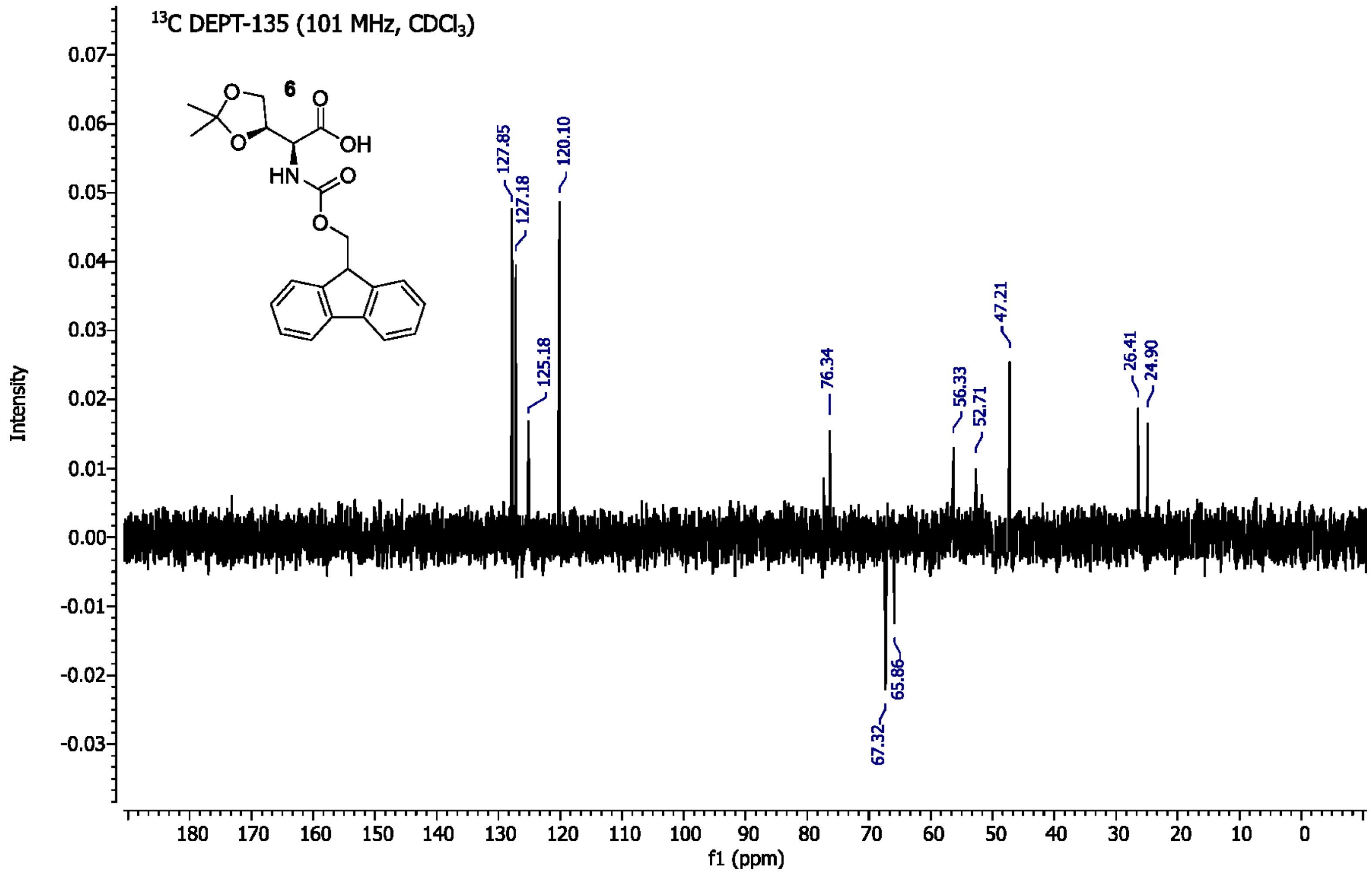

Figure S 32. ^{13}C DEPT-135 spectrum of **6**.

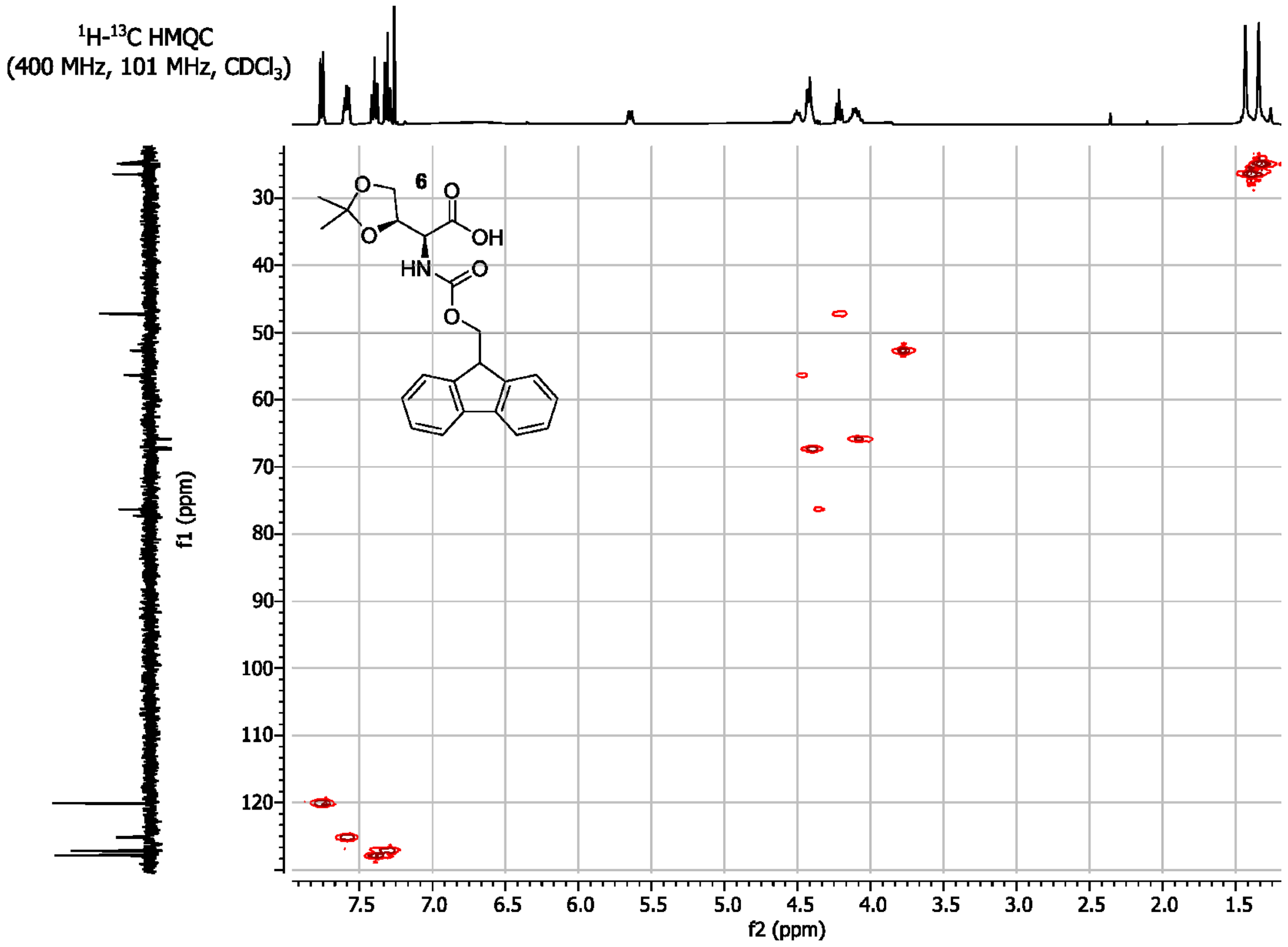

Figure S 33. ^{1}H-^{13}C HMQC spectrum of **6**.

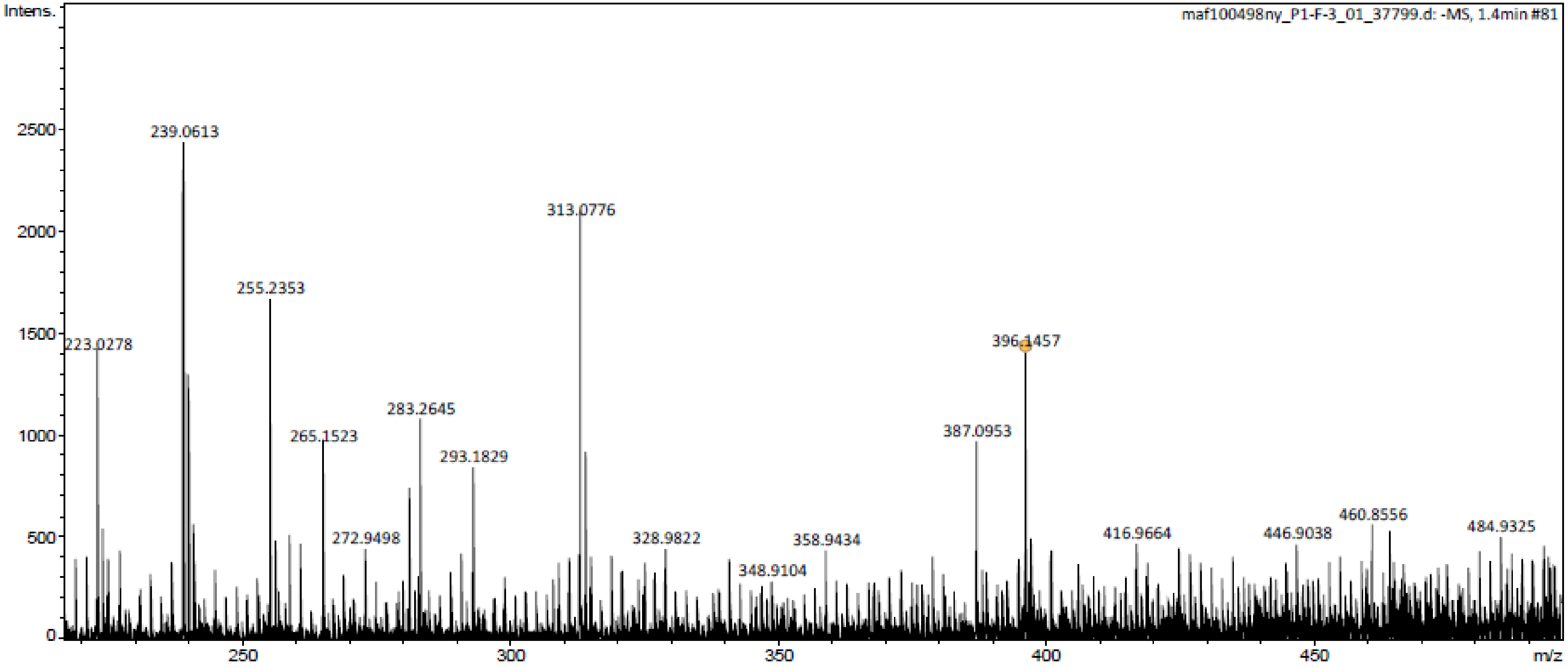

Meas. m/z	#	Ion Formula	m/z	err [ppm]	err [mDa]	mSigma	Mean err [ppm]
396.1457	1	C22H22NO6	396.1453	-1.1	-0.4	65.1	-1.3

Figure S 34. (ESI)HRMS of **6**.

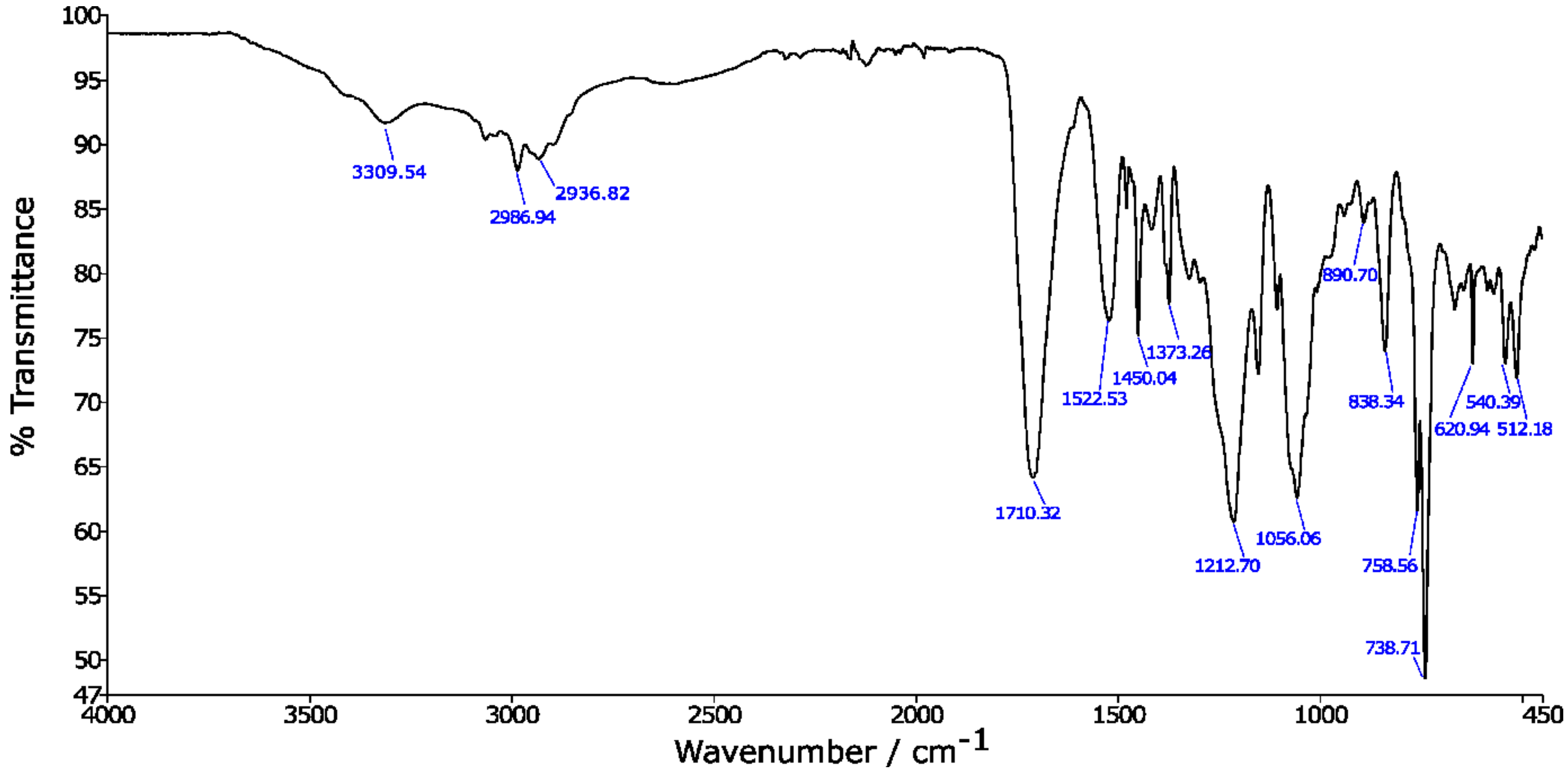

Figure S 35. FT-IR (ATR) spectrum of **6**.

Test peptide 7

7 was synthesised using Fmoc solid-phase peptide synthesis (SPPS). H-Gly-2-ClTrt resin (40 mg, 0.79 mmol/g, 0.032 mmol, styrene+1% divinylbenzene copolymer matrix, 200-400 mesh, Novabiochem) was weighed out into an SPPS cartridge fitted with a PTFE stopcock, swollen in DMF for 30 minutes and then filtered. For each amino acid coupling during the SPPS of **7** the following method was used:

DIPEA (58 µL, 0.35 mmols, 11 eq) was added to a solution of Fmoc-protected amino acid (0.16 mmols, 5 eq) and HCTU (64 mg, 0.16 mmols, 5 eq) dissolved in the minimum volume of DMF. The resultant solution was then immediately added to the resin. The reaction mixture was gently agitated by rotation for 1 h and the resin was filtered off and washed with DMF (3 x 2 minutes with rotation). A solution of 20 % piperidine in DMF was added to the resin and the mixture gently agitated by rotation for 2 minutes. The resin was filtered off and this piperidine treatment process was repeated a further four more times. The resin (now bearing deprotected amine functionality) was then washed using DMF (5 x 2 minutes with rotation) prior to coupling on the next amino acid.

The order of the amino acids used to prepare the peptide backbone of **7** were as follows: Fmoc-L-leucine, Fmoc-*O*-tert-butyl-L-tyrosine, N_{α}-Fmoc-N_{ω}-Pbf-L-arginine, fGly building block **6**, Fmoc-L-alanine, Boc-glycine.

After coupling on the final glycine residue and washing with DMF (5 x 2 minutes with rotation), the resin was washed with DCM (3 x 2 minutes with rotation) and MeOH (3 x 2 minutes with rotation). The resin was dried on a vacuum manifold and further dried on a high vacuum line overnight. 5 mL of a cleavage cocktail solution (95:2.5:2.5 TFA:H_2O:triisopropylsilane) was then added to the resin and the mixture gently agitated by rotation for 1 h. The reaction mixture was drained into cold Et_2O (incubated at -20 °C prior to use) and centrifuged at 4000 rpm at 4 °C until pelleted (ca 5-10 minutes). The supernatant was then carefully decanted. The pellet was subsequently resuspended in cold Et_2O and the centrifugation and supernatant decantation process repeated a further three more times. The precipitated peptide pellet was then dissolved in 20% AcOH (aq) and lyophilised to obtain **7** as a fluffy solid (23 mg, 95%) for use without further purification.

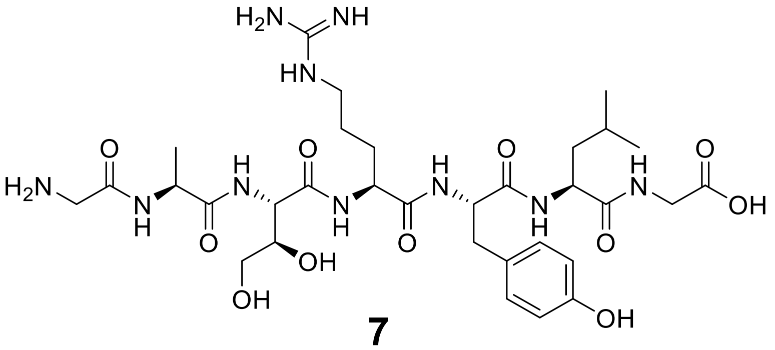

7

HRMS (ESI) m/z: $[M+H]^+$ Calcd for $C_{32}H_{53}N_{10}O_{11}$ 753.3890; Found 753.3902. $[M+Na]^+$ Calcd for $C_{32}H_{53}N_{10}O_{11}Na$ 775.3709; Found 775.3716.

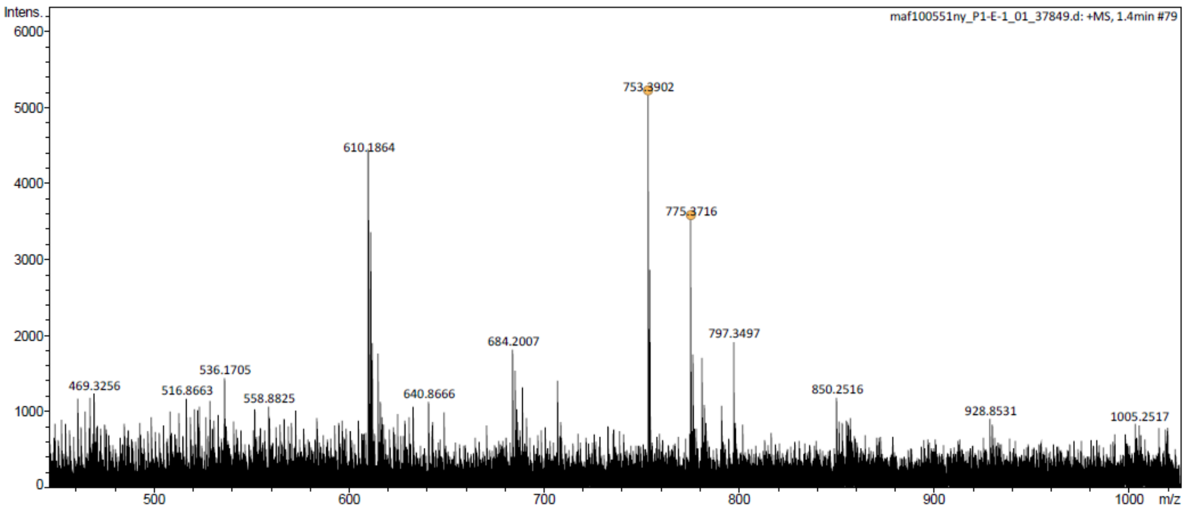

Meas. m/z	#	Ion Formula	m/z	err [ppm]	err [mDa]	mSigma	Mean err [ppm]
753.3902	1	C32H53N10O11	753.3890	-1.6	-1.2	82.6	-4.1
775.3716	1	C32H52N10NaO11	775.3709	-0.8	-0.6	55.9	-3.4

Figure S 36. (ESI)HRMS of **7**.

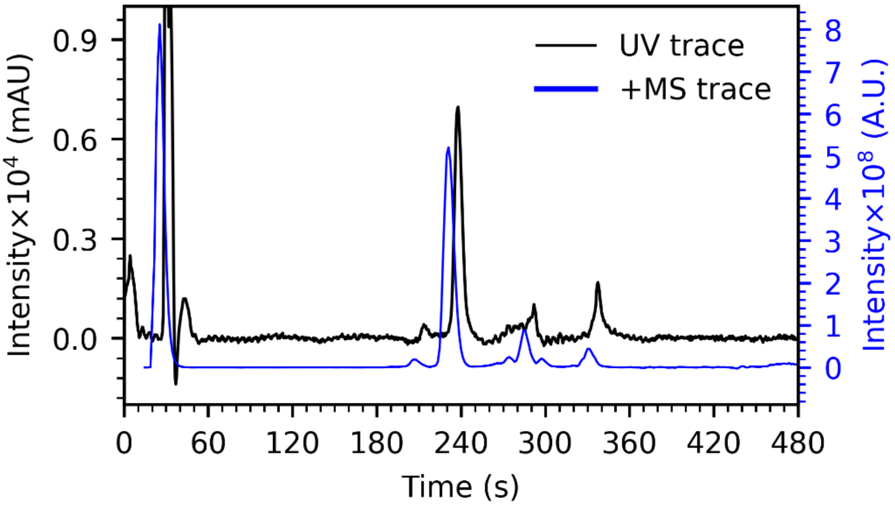

Figure S 37. LC-MS trace of test peptide **7**. The peak at ca. 230 s corresponds to **7** (see **Figure 1** of the main paper). The UV trace was recorded at 210-400 nm. The peak at ca. 10 s corresponds to injection.

Test peptide 8

To a solution of **7** (865 µL, 2.0 mM, 1.3 mg, in HPLC-grade water) was added a solution of $NaIO_4$ (42 µL, 50 mM in HPLC-grade water). The reaction was mixed thoroughly and allowed to sit for 20 minutes at rt, after which time LC-MS showed the complete conversion of **7** into **8**. The solution was then loaded onto a solid phase extraction cartridge (Grace Davison Extract Clean, 8 mL reservoir, Fisher Scientific) equilibrated with water/acetonitrile. After initial washing with water, the product was eluted over a gradient of acetonitrile. The product was then diluted with water, and subsequently lyophilised to give **8** as a white fluffy solid.

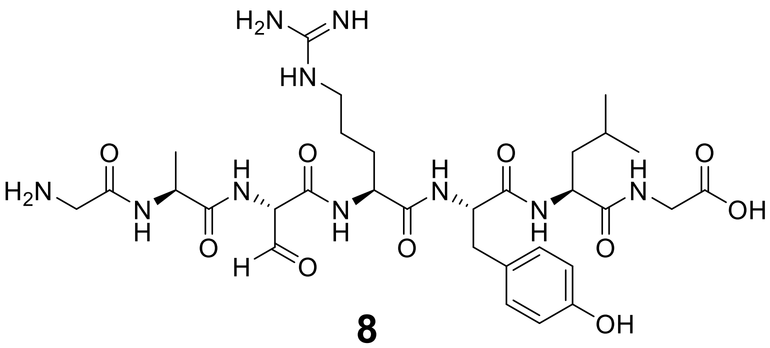

8

HRMS (ESI) m/z: [M+H]$^+$ Calcd for $C_{31}H_{48}N_{10}O_{10}$ 721.3628; Found 721.3658.

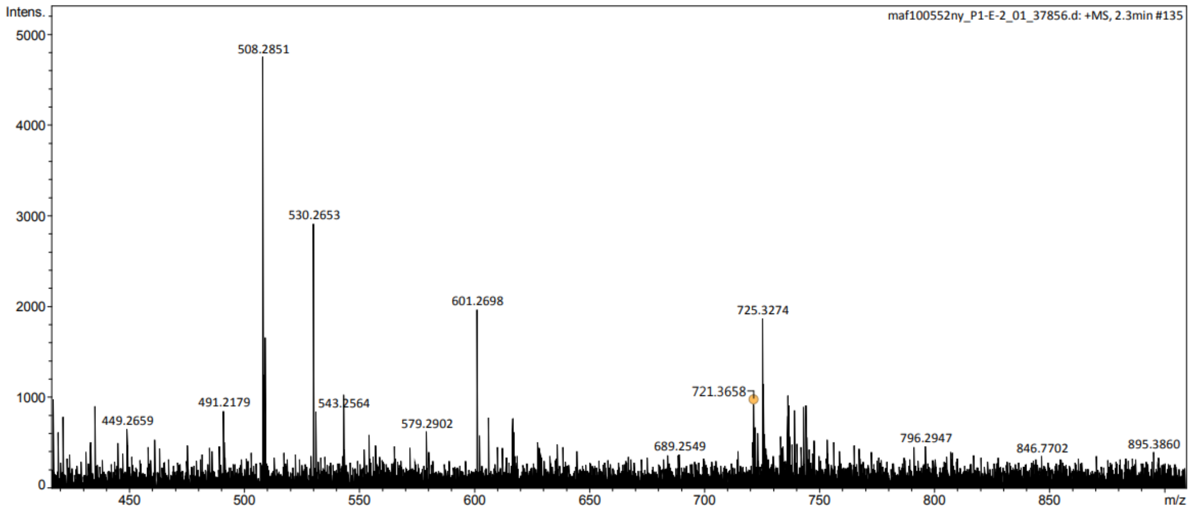

Meas. m/z	#	Ion Formula	m/z	err [ppm]	err [mDa]	mSigma	Mean err [ppm]
721.3658	1	C31H49N10O10	721.3628	-4.2	-3.1	n.a.	-1.8

Figure S 38. (ESI)HRMS of **8**.

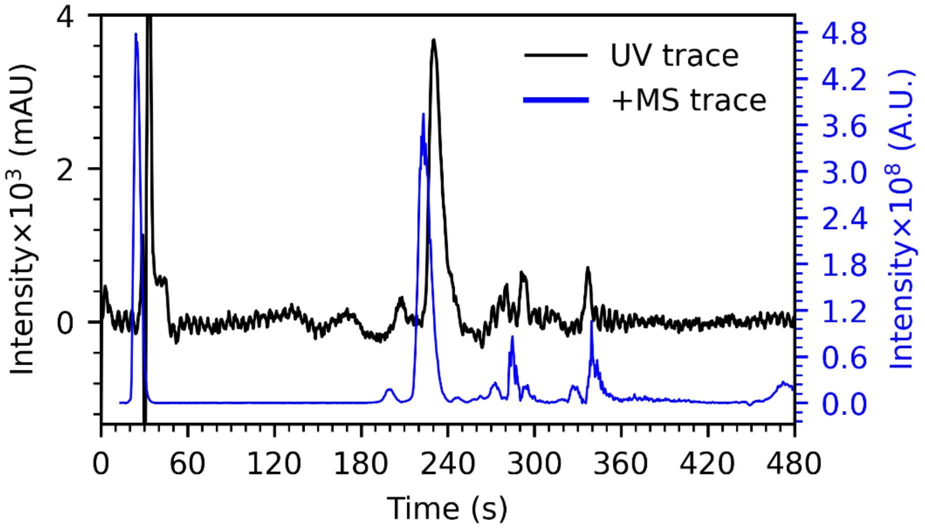

Figure S 39. LC-MS trace of test peptide **8**. The peak at ca. 220 s corresponds to **8** (see **Figure 1** of the main paper). The UV trace was recorded at 210-400 nm. The peak at ca. 10 s corresponds to injection.

Linear Callyaerin A precursor 9

Peptide **9** was synthesised via Fmoc SPPS using a CEM Liberty Lite Automated Microwave Peptide Synthesiser, according to the manufacturers standard protocols. Briefly, Fmoc-protected amino acids (5.5 equiv., 0.2 m in DMF) including fGly building block **6** were coupled in the presence of N,N'-diisopropylcarbodiimide (DIC, 15 equiv.) and Oxyma Pure (5 equiv.) under microwave irradiation at a temperature of 90 °C for 2 minutes. Fmoc deprotection was performed using 20% piperidine in DMF at 90 °C for 60 seconds. The synthesis was performed on a 0.1 mmol scale using Rink Amide MBHA resin (C-terminal amide, 0.5 mmol/g, styrene+1% divinylbenzene copolymer matrix, 100-200 mesh, Fluorochem). Prior to cleavage, the resin was washed sequentially washed with DMF (5 x 2 minutes with rotation), DCM (3 x 2 minutes with rotation) and MeOH (3 x 2 minutes with rotation). The resin was then dried on a high vacuum line overnight. 5 mL of a cleavage cocktail solution (95:2.5:2.5 TFA:H_2O:triisopropylsilane) was then added to the resin and the mixture gently agitated by rotation for 1 h. The reaction mixture was drained into cold Et_2O (incubated at -20 °C prior to use) and centrifuged at 4000 rpm at 4 °C until pelleted (ca 5-10 minutes). The supernatant was then carefully decanted. The pellet was subsequently resuspended in cold Et_2O and the centrifugation and supernatant decantation process repeated a further three more times. The precipitated peptide pellet was then dissolved in 20% AcOH (aq) and lyophilised to obtain **9** as a fluffy off-white solid (78 mg, 55%) for use without further purification.

9

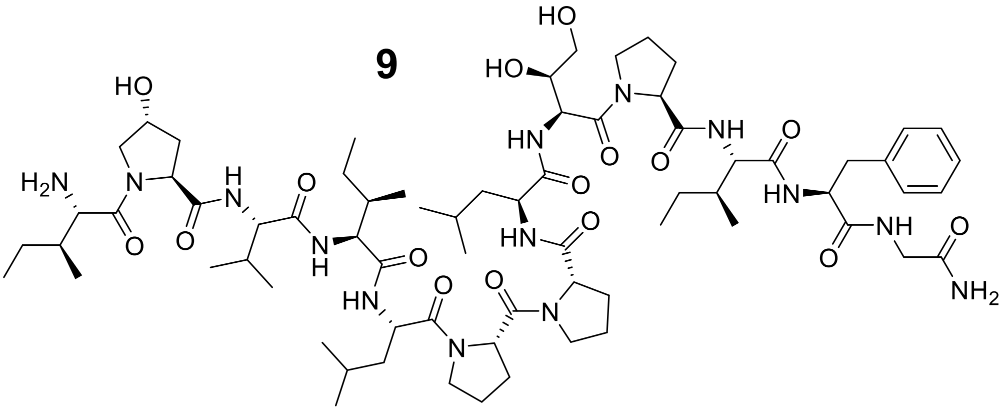

HRMS (ESI) m/z: $[M+H]^+$ Calcd for $C_{70}H_{115}N_{14}O_{16}$ 1407.8610; Found 1407.8545. $[M+Na]^+$ Calcd for $C_{70}H_{114}N_{14}O_{16}Na$ 1429.8429; Found 1429.8394. See main paper for HPLC trace.

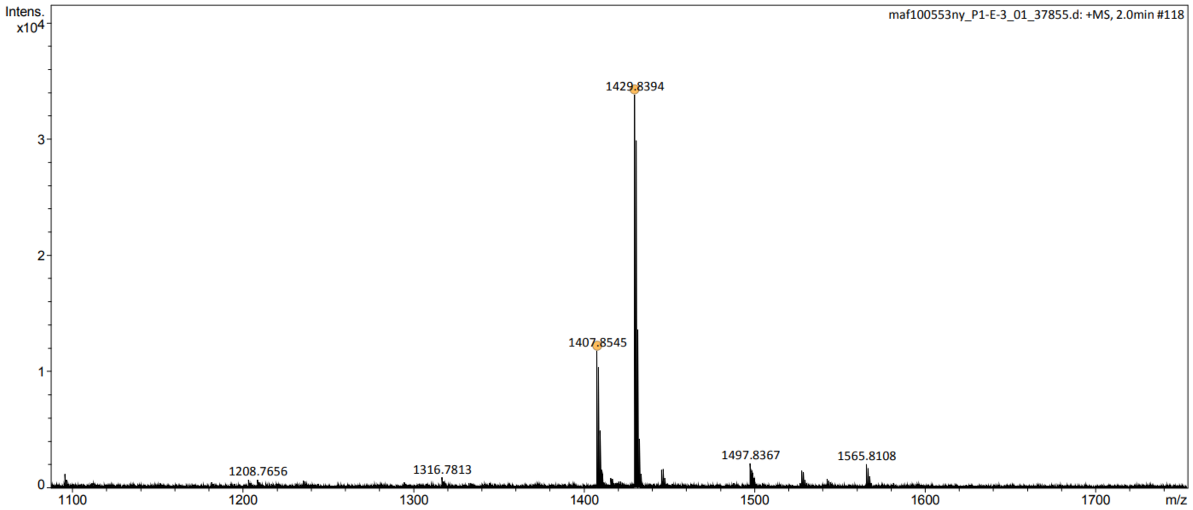

Meas. m/z	#	Ion Formula	m/z	err [ppm]	err [mDa]	mSigma	Mean err [ppm]
1407.8545	1	C70H115N14O16	1407.8610	4.6	6.5	33.2	2.8
1429.8394	1	C70H114N14NaO16	1429.8429	2.5	3.5	29.0	2.9

Figure S 40. (ESI)HRMS of **9**.

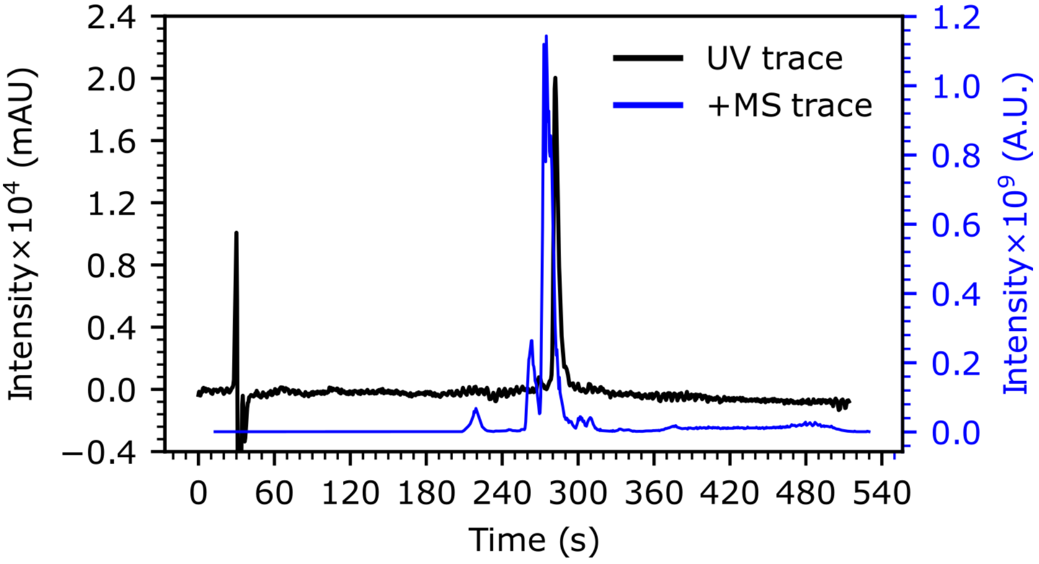

Figure S 41. LC-MS trace of **9**. The peak at ca. 280 s corresponds to **9** (see **Figure 2** of the main paper). The UV trace was recorded at 210-400 nm. The peak at ca. 10 s corresponds to injection.

Callyaerin A 10

Test scale: To a solution of **9** (350 µL, 2.0 mM, 1.0 mg, in HPLC-grade water) was added a solution of $NaIO_4$ (17 µL, 50 mM in HPLC-grade water). The reaction was mixed thoroughly and allowed to sit for 20 minutes at rt, after which time LC-MS showed the complete conversion of **9** into **9'** and **10**. The solution was then loaded onto a solid phase extraction cartridge (Grace Davison Extract Clean, 8 mL reservoir, Fisher Scientific) equilibrated with water/acetonitrile. After initial washing with water, the product was eluted over a gradient of acetonitrile. Fractions containing **10** were identified via LC-MS, diluted with water and lyophilised to give **10** as a white fluffy solid.

Prep scale: To a solution of **9** (4.6 mL, 2.1 mM, 13 mg, in HPLC-grade water) at 0 °C was added a solution of $NaIO_4$ (221 µL, 50 mM in HPLC-grade water) in 5 instalments. The reaction was mixed thoroughly and then incubated for 20 minutes at rt. NaCl (780 mg) was added to reaction solution and the mixture gently agitated until the NaCl dissolved and peptide was observed to have precipitated. MeCN (5 mL) was then added and the resultant biphasic mixture shaken and cooled to -20 °C. The organic layer was separated from the aqueous layer and set aside. A further 4 extractions of the aqueous layer using MeCN were performed in this manner. The organic extractions were then combined, dried over $MgSO_4$ and concentrated *in vacuo* to yield a crude material containing both uncyclized and cyclized Callyaerin A (**9'** and **10**). This crude material was re-dissolved in MeCN (50 mL) and $MgSO_4$ (one spatula) and formic acid (50 µL) were then added. The resultant solution was stirred for 1.5 hours at rt, after which time the $MgSO_4$ was removed via filtration and a crude sample of **10** (13 mg) was recovered by concentrating the eluate *in vacuo* (the addition of toluene to the eluate was used to assist in the removal of residual formic acid during concentration *in vacuo*).

The crude sample of **10** was further purified via reverse-phase flash chromatography on a Teledyne CombiFlash® NEXTGEN 300+ system using a C18 column (RediSep® Rf Gold C18 Reversed Phase column, 5.5 gram media) that was pre-equilibrated with water. **10** was purified using a linear gradient from water → MeCN over 10 minutes at a flow rate of 13 mL min^{-1}, holding at 100% MeCN (13 mL min^{-1}) for a further 6 minutes after the linear gradient before re-equilibrating the column back into water (see **Figure S43**). Fractions containing **10** were identified using LC-MS and were then pooled and lyophilised to give **10** (5.5 mg, 42%) as a white fluffy powder.

10

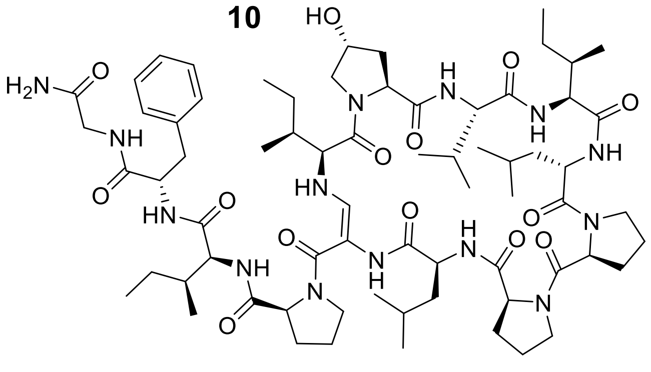

HRMS (ESI) m/z: $[M+Na]^+$ Calcd for $C_{69}H_{108}N_{14}O_{14}Na$ 1379.8062; Found 1379.8157.

^{1}H-NMR (700 MHz, DMSO-d_6): See **Figure S45**, **Figure S46** and **Table S1**.

^{13}C-NMR (176 MHz, DMSO-d_6): See **Figure S47**, **Figure S48** and **Table S1**.

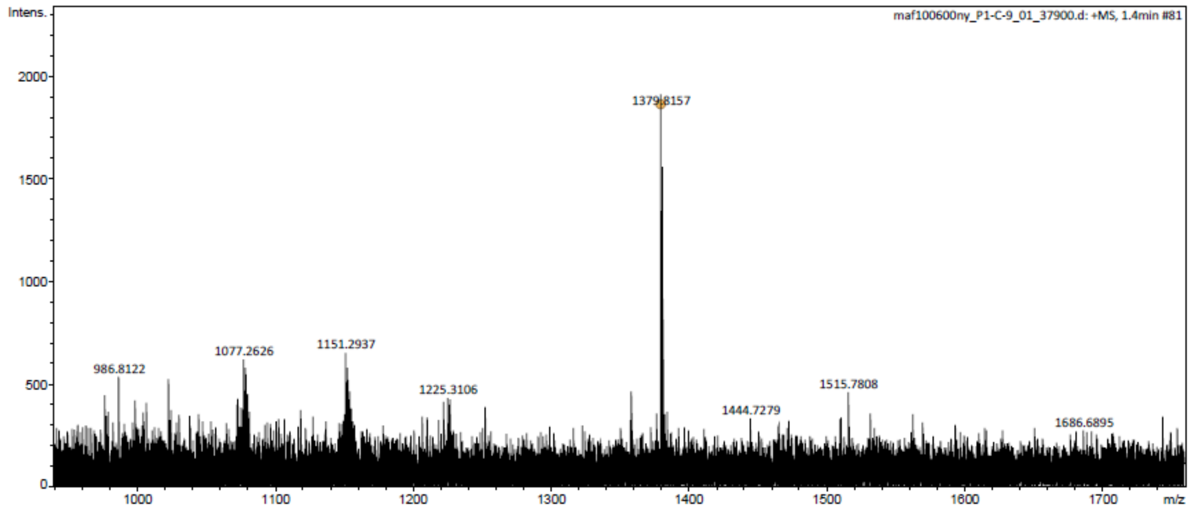

Meas. m/z	#	Ion Formula	m/z	err [ppm]	err [mDa]	mSigma	Mean err [ppm]
1379.8157	1	C69H108N14NaO14	1379.8062	-6.9	-9.5	370.2	-2.4

Figure S 42. (ESI)HRMS of **10**.

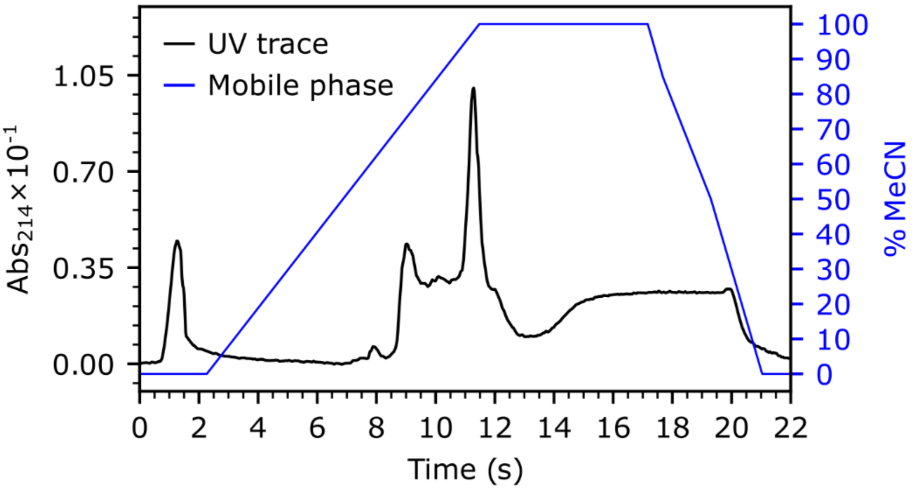

Figure S 43. Chromatographic trace recorded during the purification of **10** via reverse-phase flash chromatography on a Teledyne CombiFlash® NEXTGEN 300+ system using a C18 column (RediSep® Rf Gold C18 Reversed Phase column, 5.5 gram media). The UV trace was recorded at 214 nm, and the peak at ca. 11.5 min corresponds to **10**.

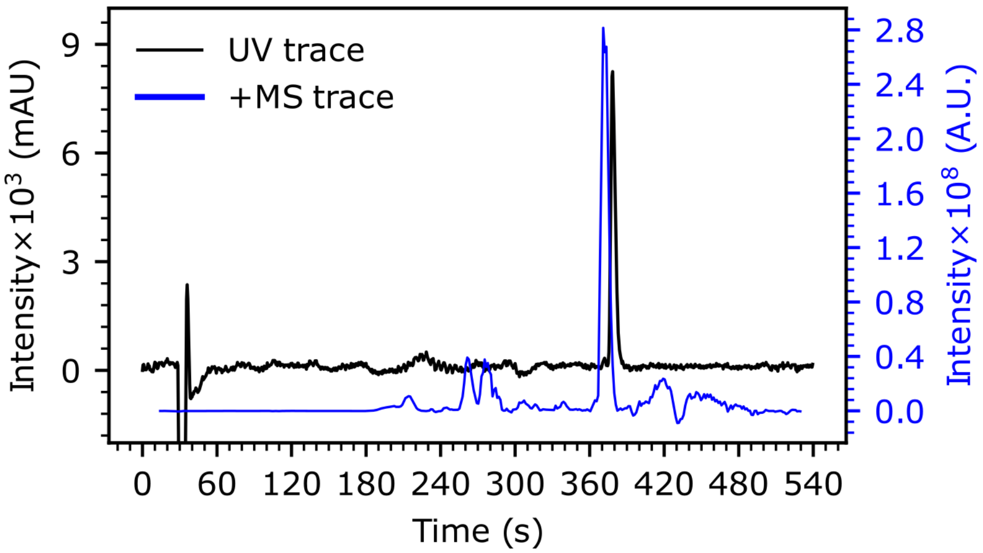

Figure S 44. LC-MS trace of a purified sample of **10**. The peak at ca. 370 s corresponds to **10** (see **Figure 2** of the main paper). The UV trace was recorded at 210-400 nm. The peak at ca. 10 s corresponds to injection.

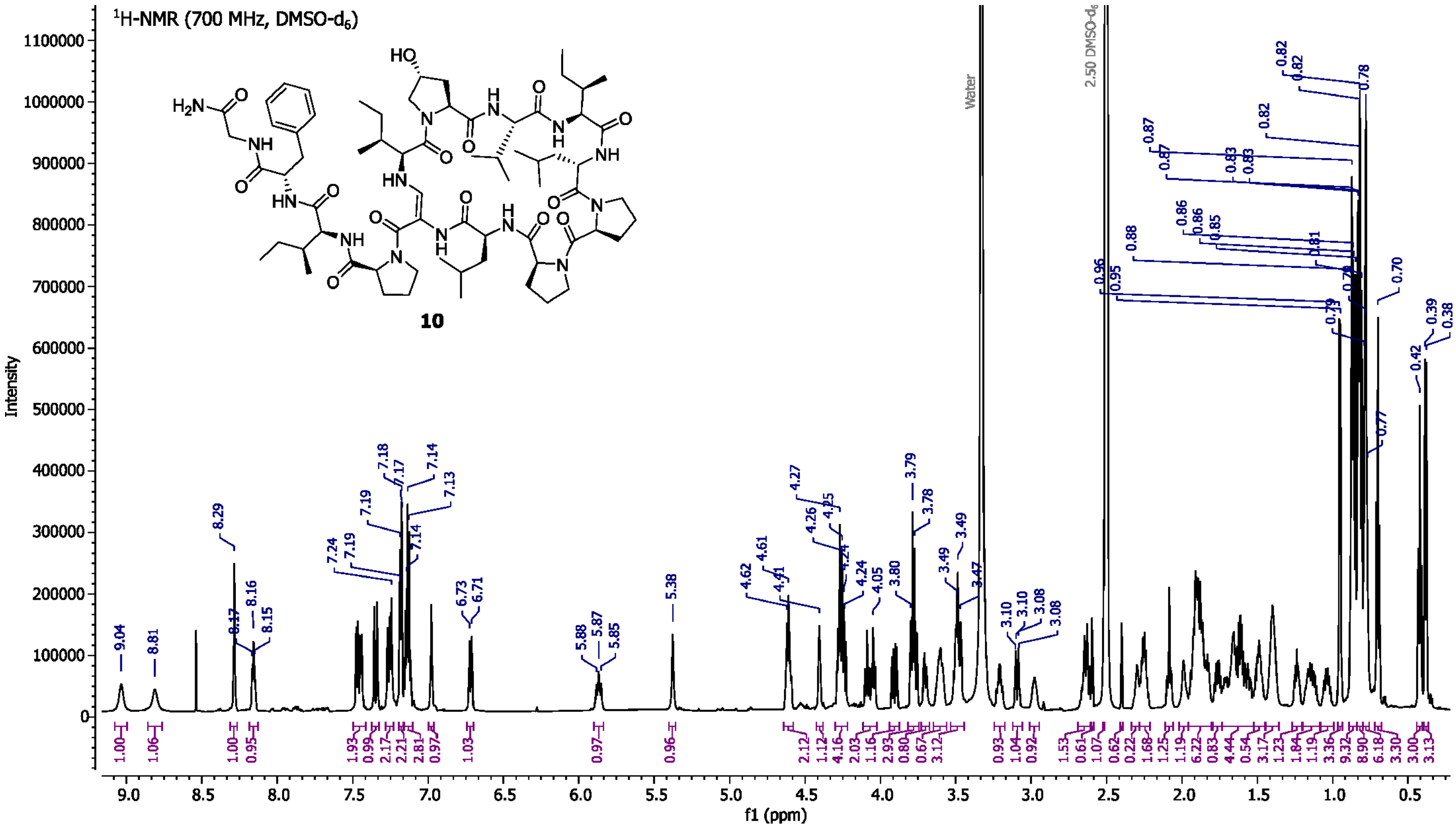

Figure S 45. 700 MHz ^{1}H-NMR spectrum of **10**.

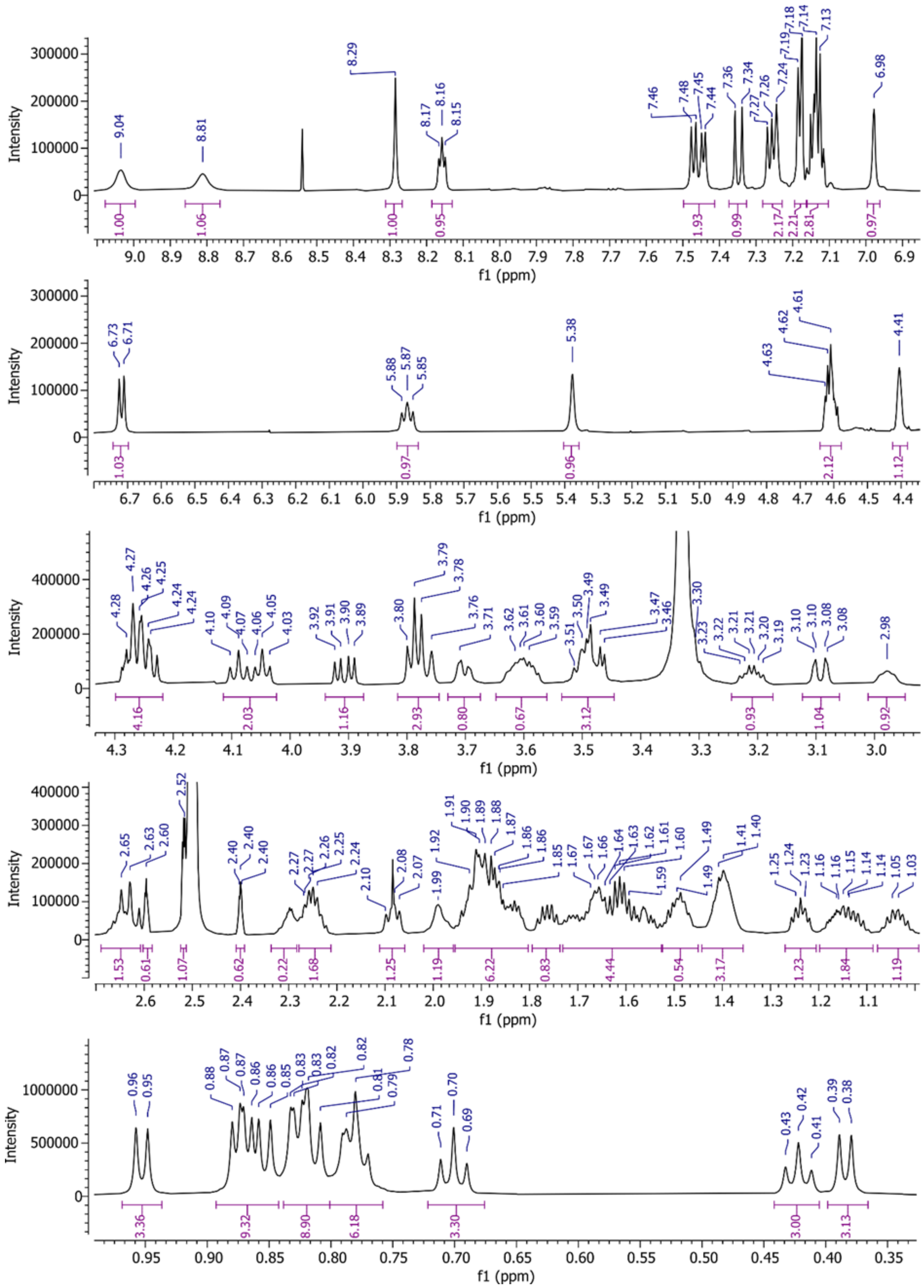

Figure S 46. Regions of the 700 MHz ^{1}H-NMR spectrum of **10**.

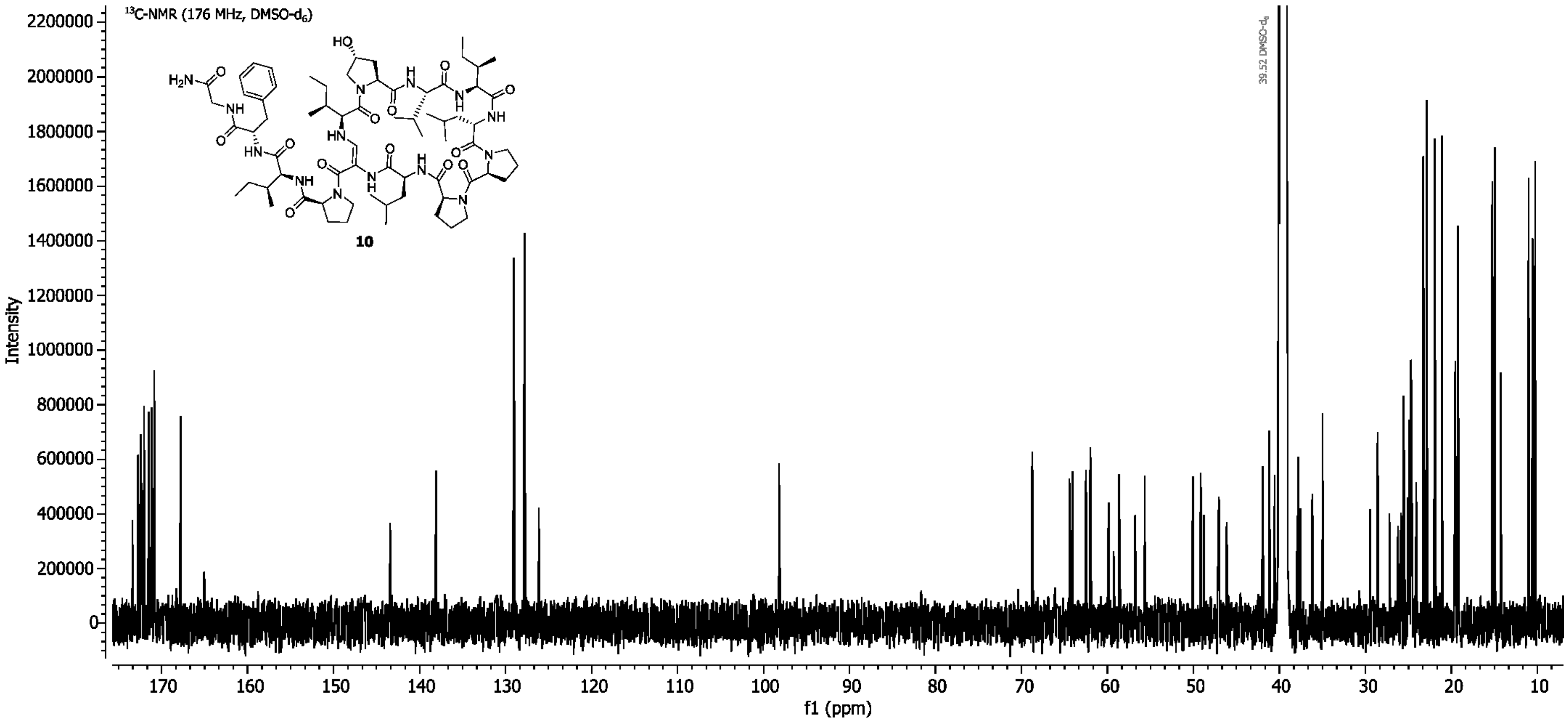

Figure S 47. 176 MHz ^{13}C-NMR spectrum of **10**.

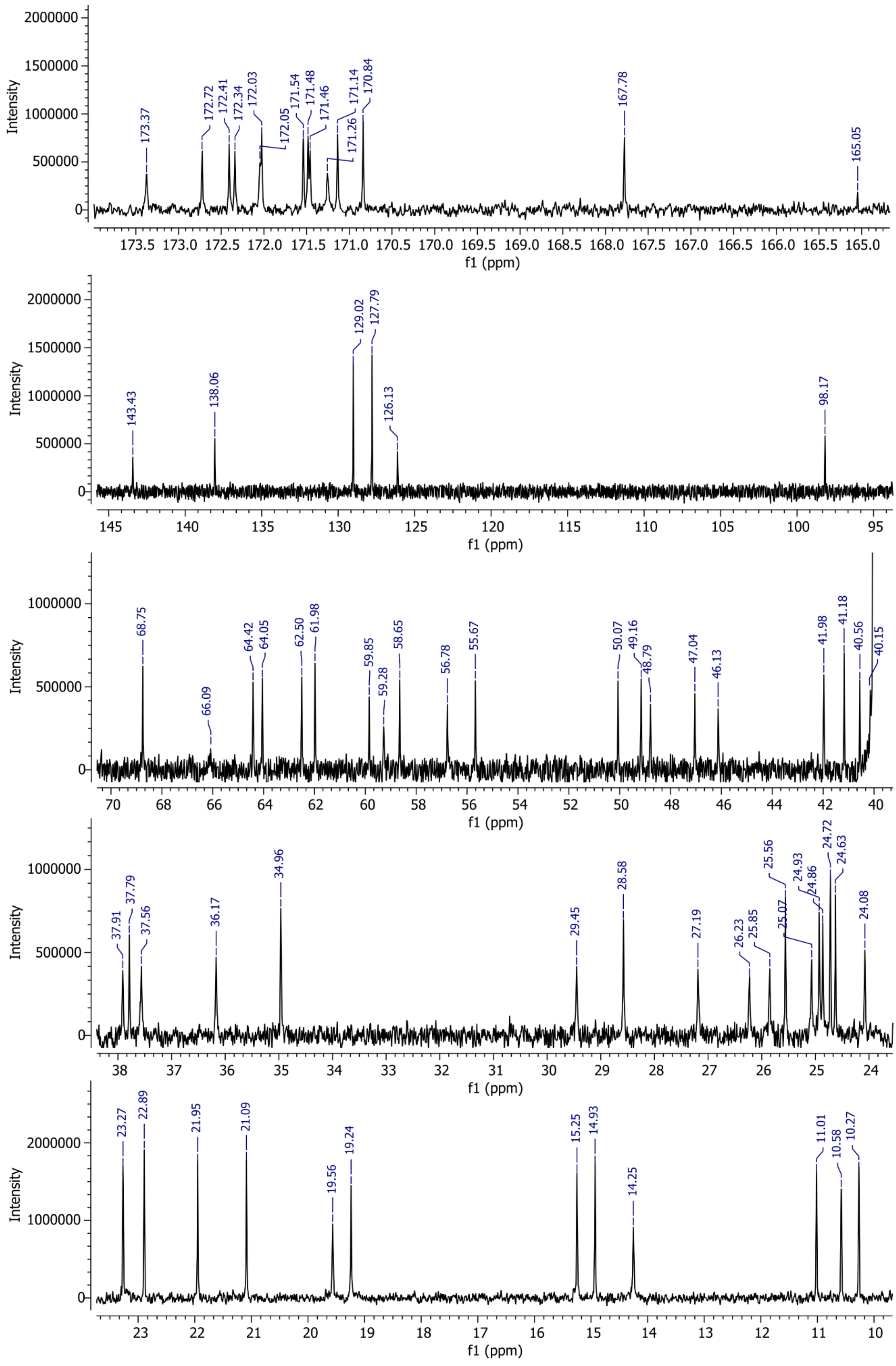

Figure S 48. Regions of the 176 MHz ^{13}C-NMR spectrum of **10**.

Table S1. Assignment of the 700 MHz ^{1}H NMR and 176 MHz ^{13}C NMR spectra of **10** cross-referenced with literature data.[3]

		**Literature[3]**		**10**	
	**Environment**	**δ_H (J in Hz)**	**δ_C**	**δ_H (J in Hz)**	**δ_C**
**Isoleucine / 1**	NH	5.87 (dd, 12.2, 10.9)	-	5.87 (dd, 12.2, 10.9)	-
	CO	-	172	-	172.0 / 172.1
	α	4.06 (m)	64.4	4.08 (m)	64.4
	β	1.40 (m)	37.8	1.40 (m)	37.8
	γ	1.40 (m), 0.79 (m)	24	1.41 (m), 0.79 (m)	24.1
	γ'	0.79 (m)	14.2	0.79 (m)	14.3
	δ	0.43 (t, 7.3)	10.6	0.42 (t, 7.33)	10.6
**Hydroxyproline / 2**	OH	5.37 (br s)	-	5.38 (s)	-
	CO	-	173.3	-	173.4
	α	4.26 (m)	55.6	4.26 (m)	55.7
	β	2.08 (m), 1.87 (m)	37.7	2.08 (m), 1.88 (m)	37.6
	γ	4.41 (m)	68.7	4.41 (m)	68.8
	δ	3.78 (m), 3.69 (d, 8.0)	56.7	3.78 (m), 3.70 (dd, 10.7, 2.12)	56.8
**Valine / 3**	NH	9.04 (br s)	-	9.04 (br s)	-
	CO	-	172	-	172.0 / 172.1
	α	2.98 (m)	66.1	2.98 (m)	66.1
	β	2.64 (m)	37.8	2.64 (m)	37.9
	γ	0.84 (m)	19.5	0.83 (m)	19.6
		0.82 (m)	19.2	0.82 (m)	19.2
**Isoleucine / 4**	NH	8.81 (br s)	-	8.81 (br s)	-
	CO	-	171.2	-	171.3
	α	3.78 (m)	59.3	3.78 (m)	59.3
	β	1.49 (m)	36.1	1.49 (m)	36.2
	γ	1.40 (m), 1.14 (m)	*	1.40 (m), 1.14 (m)	*
	γ'	0.81 (m)	15.2	0.81 (m)	15.3
	δ	0.78	10.2	0.78	10.3
**Leucine / 5**	NH	7.44 (d, 6.9)	-	7.44 (d, 7.02)	-
	CO	-	172.3	-	172.3
	α	4.61 (m)	49.1	4.61 (m)	49.2
	β	1.75 (m), 1.22 (m)	41.1	1.76 (m), 1.24 (m)	41.2
	γ	1.63 (m)	*	1.63 (m)	*
	δ	0.88 (m)	21	0.88 (m)	21
	δ'	0.87 (m)	21	0.87 (m)	21
**Proline / 6**	CO	-	171.4	-	171.5 / 171.5
	α	4.06 (m)	64	4.05 (m)	64.0
	β	2.26 (m), 1.87 (m)	26.2	2.26 (m), 1.87 (m)	26.2
	γ	1.98 (m), 1.87 (m)	*2	1.99 (m), 1.87 (m)	*2
	δ	3.60 (m), 3.48 (m)	46.1	3.61 (m), 3.47 (m)	46.1

**Proline / 7**	CO	-	171.4	-	171.5 / 171.5
	α	4.26 (m)	62.5	4.26 (m)	62.5
	β	2.26 (m), 1.63 (m)	28.5	2.26 (m), 1.63 (m)	28.6
	γ	1.93 (m), 1.87 (m)	*2	1.93 (m), 1.87 (m)	*2
	δ	3.60 (m), 3.31 (m)	47	3.60 (m), 3.30 (m)	47.0
**Leucine / 8**	NH	6.72 (d, 10.0)	-	6.72 (d, 10.0)	-
	CO	-	172.4	-	172.4
	α	4.61 (m)	50	4.61 (m)	50.1
	β	1.87 (m), 1.58 (m)	40.5	1.88 (m), 1.58 (m)	40.6
	γ	1.63 (m)	*	1.64 (m)	*
	δ	0.96 (d, 6.6)	23.2	0.95 (d, 6.57)	23.3
	δ'	0.85 (m)	22.8	0.85 (m)	22.8
**DAA / 9**	NH	8.29 (s)	-	8.29 (s)	-
	CO	-	167.7	-	167.8
	α	-	98.2	-	98.2
	β	7.35 (d, 13.2)	143.4	7.35 (d, 13.37)	143.4
**Proline / 10**	CO	-	172.7	-	172.7
	α	4.26 (m)	61.9	4.26 (m)	62.0
	β	2.26 (m), 1.49 (m)	29.4	2.26 (m), 1.49 (m)	29.5
	γ	1.87 (m), 1.75 (m)	*2	1.87 (m), 1.76 (m)	*2
	δ	3.48 (m), 3.21 (m)	48.7	3.48 (m), 3.21 (m)	48.8
**Isoleucine / 11**	NH	7.47 (d, 8.8)	-	7.47 (d, 8.71)	-
	CO	-	171.1	-	171.1
	α	3.78 (m)	58.6	3.78 (m)	56.7
	β	1.63 (m)	34.9	1.63 (m)	35.0
	γ	1.14 (m), 1.05 (m)	*	1.14 (m), 1.05 (m)	*
	γ'	0.39 (d, 6.7)	14.9	0.39 (d, 6.72)	14.9
	δ	0.70 (t, 7.4)	11	0.70 (t, 7.33)	11.0
**Phenylalanine / 12**	NH	7.26 (d, 8.9)	-	7.26 (d, 8.63)	-
	CO	-	171.5	-	171.5
	α	4.26 (m)	59.3	4.26 (m)	59.3
	β	3.10 (d, 11.4), 2.64 (m)	39.9	3.10 (dd, 13.8, 2.15), 2.64 (m)	40.2
	Aromatic signals	7.19-7.19 (m)	138<br>129<br>127.7<br>126.1	3.10 (dd, 13.8, 2.15), 2.64 (m)	138.1<br>129.0<br>127.8<br>126.1
**Glycine / 13**	NH	8.17 (t, 6.1)	-	8.16 (t, 6.14)	-
	CO	-	170.8	-	170.8
	α	3.91 (dd, 16.5, 7.1), 3.48 (m)	42	3.91 (dd, 16.62, 7.15), 3.48 (m)	42.0
	NH_2	7.25 (br s), 6.98 (br s)	-	7.24 (br s), 6.98 (br s)	-

* = One of the signals at 24.6, 24.7, 24.8 or 24.9 ppm.
*2 = One of the signals at 25.0, 25.5 or 25.8 ppm.

Additional Figures

7

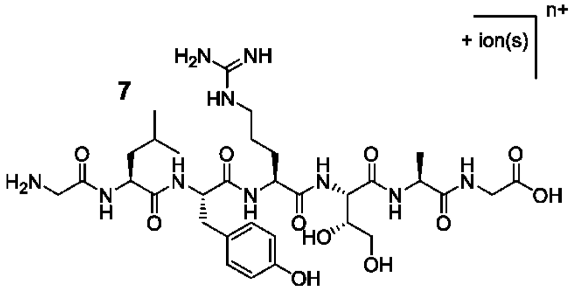

Ion(s)	Chemical Formula	m/z
H^+	$C_{32}H_{53}N_{10}O_{11}^+$	753.3890
Na^+	$C_{32}H_{52}N_{10}NaO_{11}^+$	775.3709

8

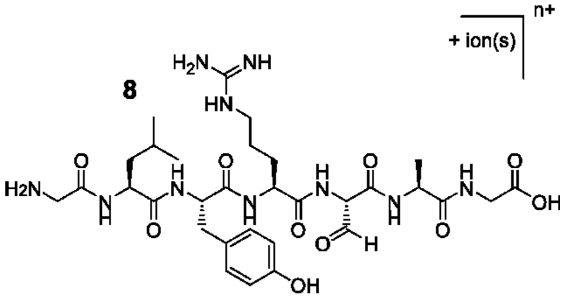

Ion(s)	Chemical Formula	m/z
H^+	$C_{31}H_{49}N_{10}O_{10}^+$	721.3628
Na^+	$C_{31}H_{48}N_{10}NaO_{10}^+$	743.3448

8 (hydrate)

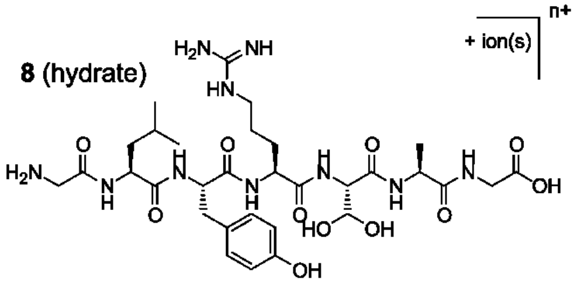

Ion(s)	Chemical Formula	m/z
H^+	$C_{31}H_{51}N_{10}O_{12}^+$	739.3733

Figure S 49. Mass spectroscopy assignments for the peaks in **Figure 1**

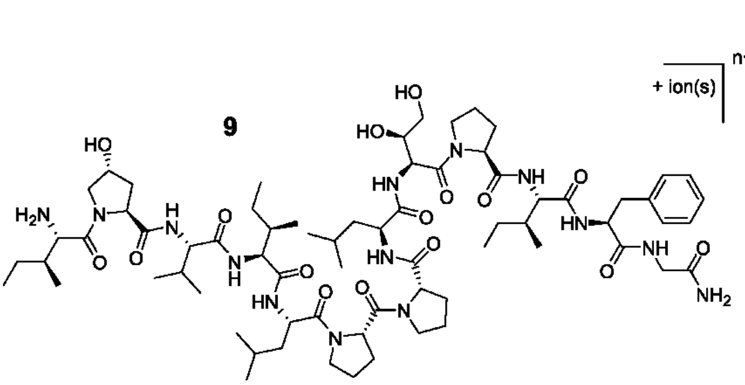

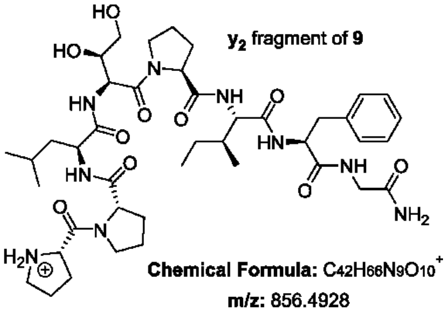

Ions	Chemical Formula	m/z
H^+	$C_{70}H_{115}N_{14}O_{16}^{+}$	1407.861
Na^+	$C_{70}H_{114}N_{14}NaO_{16}^{+}$	1429.843
$2H^+$	$C_{70}H_{116}N_{14}O_{16}^{2+}$	704.4342
$H^+ + Na^+$	$C_{70}H_{115}N_{14}NaO_{16}^{2+}$	715.4251

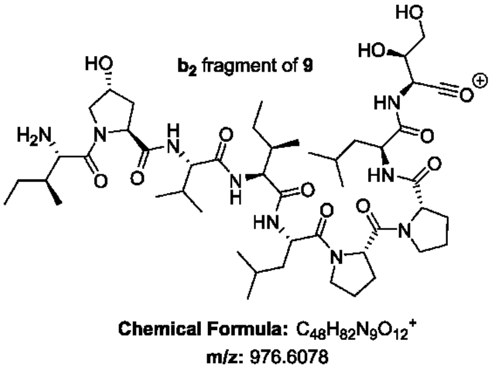

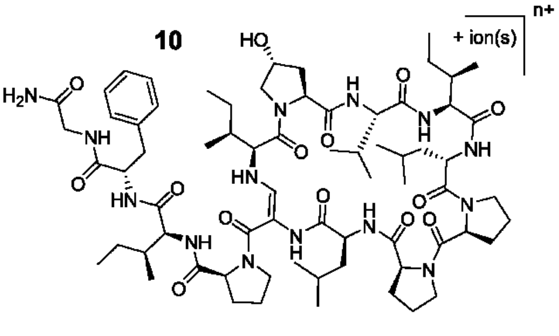

Ion(s)	Chemical Formula	m/z
H^+	$C_{69}H_{109}N_{14}O_{14}^{+}$	1357.824
Na^+	$C_{69}H_{108}N_{14}NaO_{14}^{+}$	1379.806
$2H^+$	$C_{69}H_{110}N_{14}O_{14}^{2+}$	679.4158
$H^+ + Na^+$	$C_{69}H_{109}N_{14}NaO_{14}^{2+}$	690.4067
$2Na^+$	$C_{69}H_{108}N_{14}Na_2O_{14}^{2+}$	701.3977

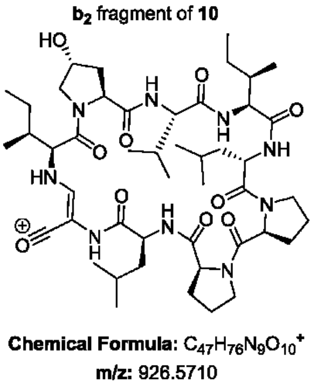

Figure S 50. Mass spectrometry assignments for the peaks in **Figure 2**.

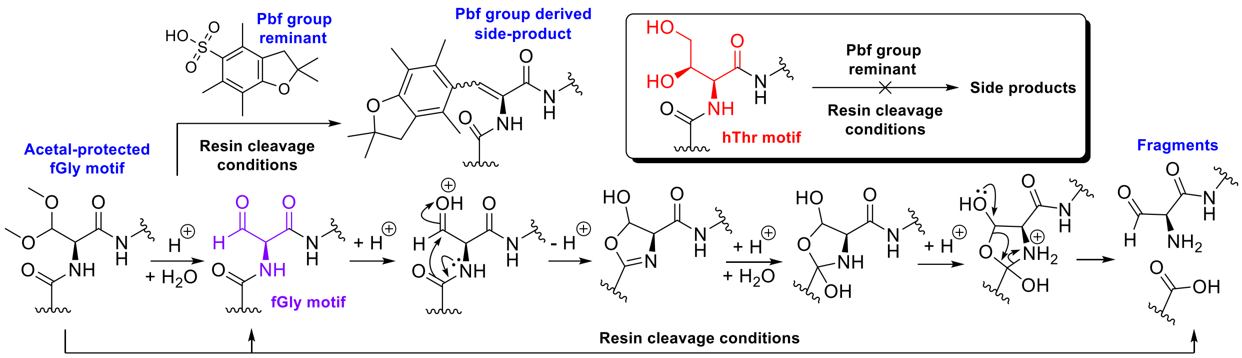

Figure S 51. Use of an hThr motif as an fGly precursor can circumvent complications associated with the acid catalysed fragmentation of/nucleophilic attack on fGly motif during SPPS resin cleavage, as illustrated here for the acetal protected fGly motif incorporated using the previously reported acetal protected fGly building block.[4]

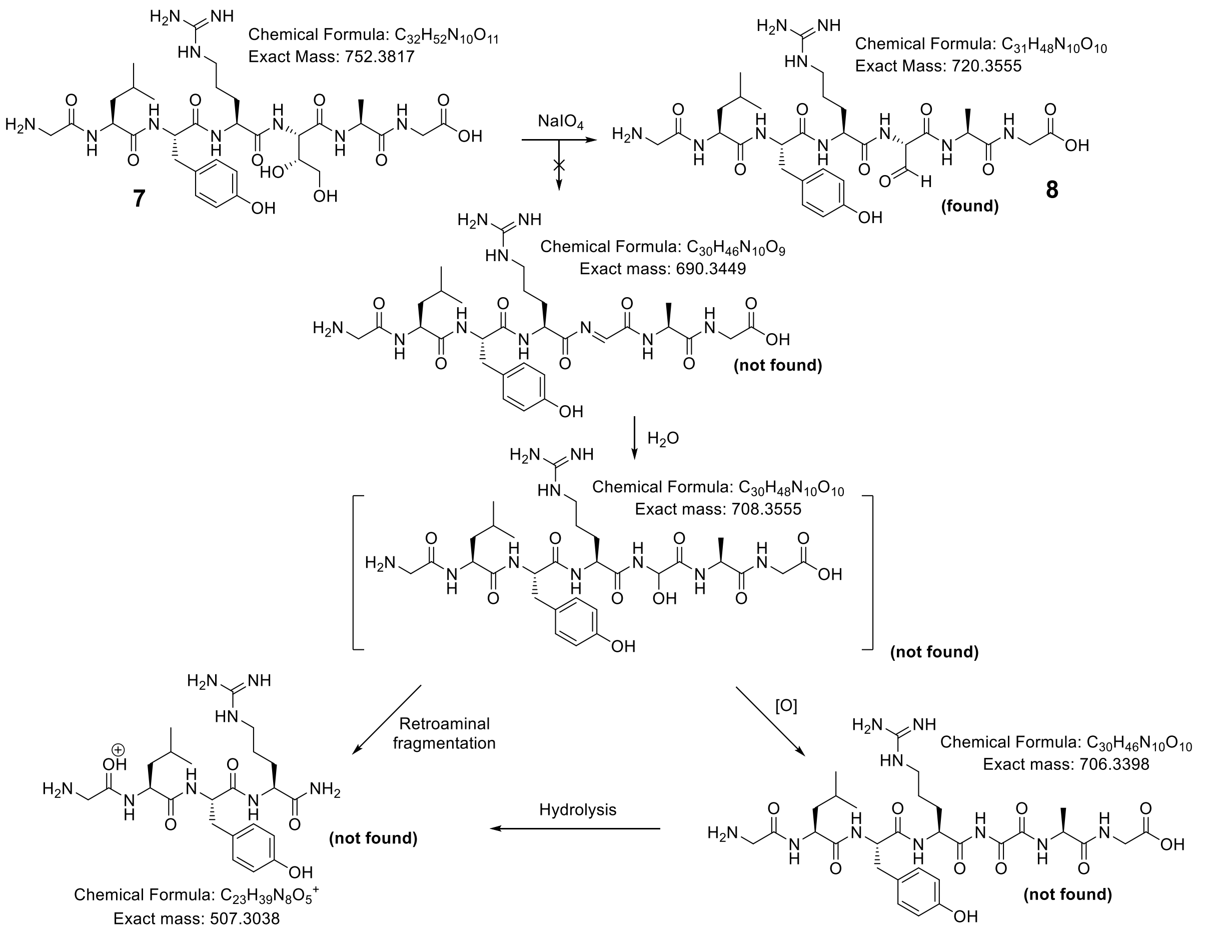

Figure S 52. The oxidation of serine with RuO_4 is known to lead to oxidative scission and retroaminal fragmentation pathways.[5] By contrast, during mass spectrometry analysis of the oxidation of **7** to **8** using $NaIO_4$, fragmentation products attributable to oxidative scission/retroaminal fragmentation were not observed, suggesting that the oxidation of the 4-hydroxythreonine (hThr) motif to fGly using $NaIO_4$ is mild and selective.

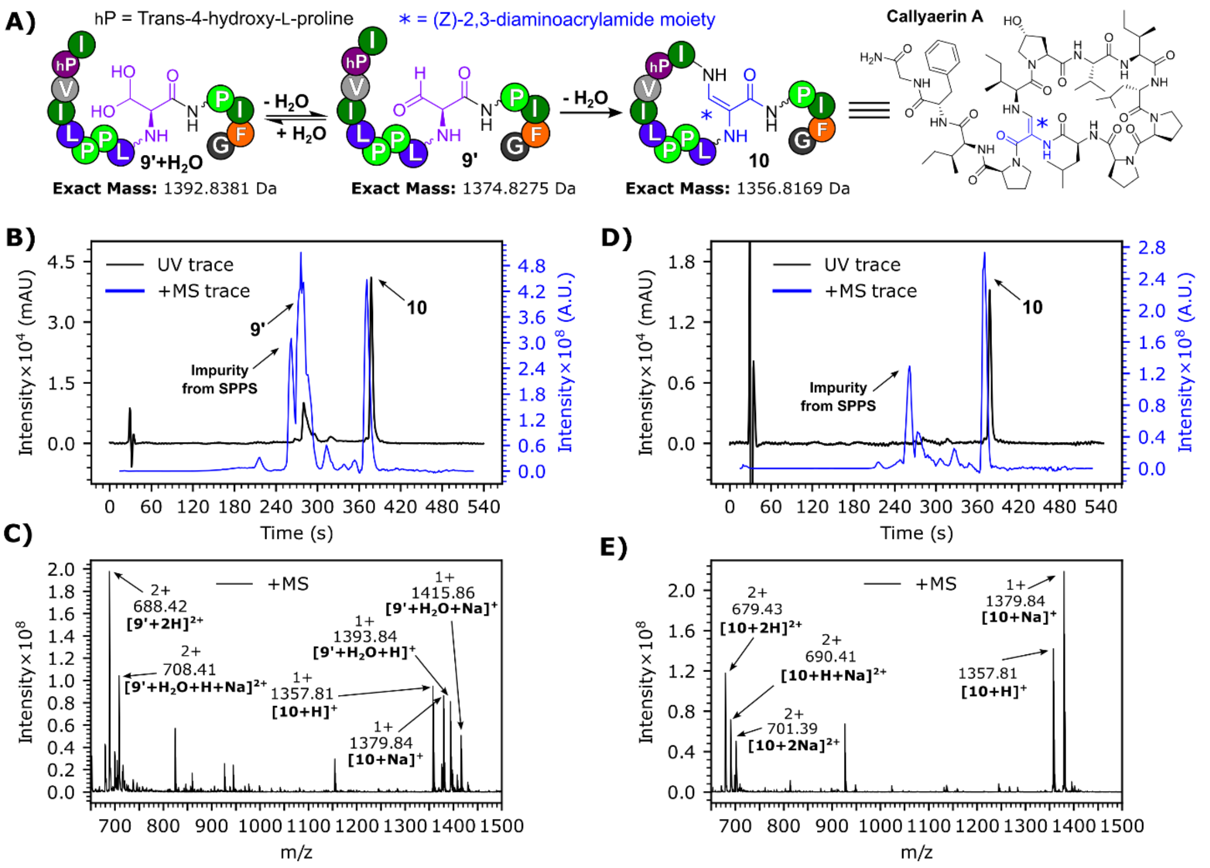

Figure S 53. A) The cyclisation of **9'** to **10**. **B)** LC-MS chromatogram of a mixture of **9'** and **10** freshly prepared via the treatment of **9** with $NaIO_4$. **C)** Mass spectrum of mixture of **9'** and **10** freshly prepared via the treatment of **9** with $NaIO_4$. **D)** LC-MS chromatogram of a sample of **10** prepared via the incubation of a **9'/10** mixture in MeCN + 0.1% formic acid for 1.5 hours. **E)** Mass spectrum of a crude sample of **10** prepared via the incubation of a **9'/10** mixture in MeCN + 0.1% formic acid for 1.5 hours. Note that the peak at m/z 926.57 corresponds to a b_2 fragment ion of **10**. The impurity from SPPS is present in **9** and has been carried through into the samples of **9'/10** presented here. While this species ionises efficiently, the UV traces indicate that it is an abundant species in the above samples. This impurity is unaffected by the treatment with $NaIO_4$. LC-MS chromatograms show the BPC+All MS trace and the UV chromatogram shows the absorbance for wavelengths 210-400 nm. LC-MS was conducted using LC gradient B (see **Figure S2**).

References

[1] M. E. Jung, T. J. Shaw, *J. Am. Chem. Soc.* **1980**, *102*, 6304-6311.

[2] K. Danielmeier, E. Steckhan, *Tetrahedron: Asymmetry* **1995**, *6*, 1181-1190.

[3] (a) S. Zhang, L. M. De Leon Rodriguez, I. K. H. Leung, G. M. Cook, P. W. R. Harris, M. A. Brimble, *Angew. Chem., Int. Ed.* **2018**, *57*, 3631-3635; (b) S. R. M. Ibrahim, C. C. Min, F. Teuscher, R. Ebel, C. Kakoschke, W. Lin, V. Wray, R. Edrada-Ebel, P. Proksch, *Biorg. Med. Chem.* **2010**, *18*, 4947-4956.

[4] J. Rush, C. R. Bertozzi, *Org. Lett.* **2006**, *8*, 131-134.

[5] D. Ranganathan, N. K. Vaish, K. Shah, *J. Am. Chem. Soc.* **1994**, *116*, 6545-6557.
